# Wolbachia Endosymbionts Modify Drosophila Ovary Protein Levels in a Context-Dependent Manner

**DOI:** 10.1128/AEM.01255-16

**Published:** 2016-08-15

**Authors:** Steen Christensen, Ricardo Pérez Dulzaides, Victoria E. Hedrick, A. J. M. Zehadee Momtaz, Ernesto S. Nakayasu, Lake N. Paul, Laura R. Serbus

**Affiliations:** a Department of Biological Sciences, Biomolecular Sciences Institute, Florida International University, Miami, Florida, USA; b Bindley Bioscience Center, Purdue Proteomics Facility, Purdue University, West Lafayette, Indiana, USA; University of Bayreuth

## Abstract

Endosymbiosis is a unique form of interaction between organisms, with one organism dwelling inside the other. One of the most widespread endosymbionts is Wolbachia pipientis, a maternally transmitted bacterium carried by insects, crustaceans, mites, and filarial nematodes. Although candidate proteins that contribute to maternal transmission have been identified, the molecular basis for maternal Wolbachia transmission remains largely unknown. To investigate transmission-related processes in response to Wolbachia infection, ovarian proteomes were analyzed from Wolbachia-infected Drosophila melanogaster and D. simulans. Endogenous and variant host-strain combinations were investigated. Significant and differentially abundant ovarian proteins were detected, indicating substantial regulatory changes in response to Wolbachia. Variant Wolbachia strains were associated with a broader impact on the ovary proteome than endogenous Wolbachia strains. The D. melanogaster ovarian environment also exhibited a higher level of diversity of proteomic responses to Wolbachia than D. simulans. Overall, many Wolbachia-responsive ovarian proteins detected in this study were consistent with expectations from the experimental literature. This suggests that context-specific changes in protein abundance contribute to Wolbachia manipulation of transmission-related mechanisms in oogenesis.

**IMPORTANCE** Millions of insect species naturally carry bacterial endosymbionts called Wolbachia. Wolbachia bacteria are transmitted by females to their offspring through a robust egg-loading mechanism. The molecular basis for Wolbachia transmission remains poorly understood at this time, however. This proteomic study identified specific fruit fly ovarian proteins as being upregulated or downregulated in response to Wolbachia infection. The majority of these protein responses correlated specifically with the type of host and Wolbachia strain involved. This work corroborates previously identified factors and mechanisms while also framing the broader context of ovarian manipulation by Wolbachia.

## INTRODUCTION

Symbiotic interactions between organisms, ranging from lethal parasitism to indispensable mutualism, frame the foundation of life. Endosymbionts face the same challenges as other microbes, which must replicate well and spread efficiently to be successful. However, the molecular mechanisms that contribute to endosymbiont transmission are not yet well understood. Endosymbiotic Wolbachia bacteria provide an excellent system to address this knowledge gap. These alphaproteobacteria of the Rickettsiales order are highly successful in nature, infecting filarial nematodes, crustaceans, mites, and over 40% of all insect species, including the well-established model organism Drosophila melanogaster (1–4). The presence of Wolbachia among this wide range of hosts is due to effective maternal transmission, analogous to mitochondria (1, 5–7). The ovary produces egg chambers, composed of germ line and somatic cells, that mature over 3 to 4 days into completed eggs (8). Wolbachia bacteria are loaded into egg chambers through vertical and horizontal transmission (9–14), intracellular replication (15, 16), and achievement of transmission-enhancing localization patterns (17–20). The actin cytoskeleton also contributes to maternal Wolbachia transmission by facilitating germ line colonization through an unknown mechanism (21).

With the success of Wolbachia being reliant upon maternal germ line cells, it is in the interest of Wolbachia to enhance host fecundity (22–24). Wolbachia bacteria are thought to achieve this in part by increasing the frequency of germ line stem cell division (11). Other studies indicate that Wolbachia bacteria support ovary productivity by enabling proactive management of toxic iron (25–28), suppressing Sex-lethal (*Sxl*) mutations (29), and preventing generalized apoptosis in the germ line (11, 30). The specific factors involved in executing these Wolbachia impacts on the host germ line are not yet clear. Studies have used a variety of approaches to investigate expression-related host responses to Wolbachia (31–42). These analyses of Wolbachia-infected cultured cell lines, invertebrate body tissues, and intact host organisms to date have yielded a wealth of information. When considering how Wolbachia bacteria interact with and manipulate host germ line cells, the implications of this diverse set of findings are unclear, however. This study examines the hypothesis that consensus molecular interactions between Wolbachia and the host contribute to maternal Wolbachia transmission. The objective of this study was to assess the conservation of Wolbachia-host interaction mechanisms through analysis of the ovarian proteome.

## MATERIALS AND METHODS

### Wolbachia strain genotyping.

Wolbachia genotyping was performed according to a diagnostic assay based on variable-number tandem-repeat (VNTR) and IS*5* markers (43). The profile of fragment sizes matched each sample with a known strain type. These same fragments were amplified from Drosophila simulans wRi as a negative control. To further distinguish *w*Mel^CS^ from the highly similar strain *w*Mel^Pop^, we used previously outlined diagnostic approaches (44). Sequencing was performed to identify a potential single nucleotide polymorphism (SNP) substitution at position 943,443 in the *w*Mel^CS^ genome. Samples were analyzed on an ABI 3100 genetic analyzer with sequencing analysis and GeneScan software (Applied Biosystems, CA). Octomom copy numbers were determined by quantitative real-time PCR (qRT-PCR) as previously reported (44). PCRs were carried out with Maxima SYBR green/ROX quantitative PCR (qPCR) master mix, using a CFX Connect real-time PCR detection system (Bio-Rad). Data were analyzed with CFX Manager V.3.1. Relative Octomom copy numbers for each Wolbachia-infected host combination were calculated by methods reported previously (45).

### Fly strains and rearing.

The D. melanogaster genetic background used for all proteomic analyses was an uninfected *w*;*Sp/Cyo*;*Sb/TM6B* strain. *w*Mel and *w*Mel^CS^ strains described in previous studies were crossed into this line to ensure a uniform genetic background for all experiments (17, 44). The strain of D. simulans used as a control was a *w*^*−*^ stock that was cured of Wolbachia with tetracycline 10 years ago. The *w*Ri Wolbachia strain endogenous to D. simulans and the *w*Mel Wolbachia strain transfected into D. simulans (46) were backcrossed into the cured fly stock for six generations to standardize the D. simulans genetic background used in this study.

All Drosophila populations used in this study were maintained at 25°C on a 12-h light/dark cycle. The flies were housed in bottles containing food that was generated in-house, based upon a modified Bloomington Stock Center recipe for Drosophila medium (47, 48). The fly food was prepared in batches consisting of 20 liters water, 337 g yeast, 190 g soy flour, 1,325 g yellow corn meal, 96 g agar, 1.5 liters Karo light corn syrup, and 94 ml propionic acid.

### Sample collection for proteomic analysis.

Flies were collected from equally aged generations during the first 3 days of eclosion only. Food bottles were cleared, and newly emerged flies were collected after 24 h to ensure equal age among parallel experimental cohorts. Flies were then aged for 2 days in vials and subsequently transferred onto new food for 3 more days. Comparability between samples was maximized by running all procedures in parallel for the flies used in each biological replicate. Samples were prepared from a minimum of 25 flies for each biological replicate for all six Wolbachia host-strain combinations. Ovaries were dissected in 0.8 to 1.0 ml of ice-cold lysis buffer (50 mM NH_4_HCO_3_, 1 mM EDTA, 2 mM sodium vanadate). The uninfected and cured flies were always dissected first, and all dissection equipment (i.e., dissection dish and tweezers) was cleaned with 70% ethanol and rinsed with lysis buffer between dissections for each sample. Immediately after dissection, ovarian samples were imaged on a Leica MZ6 stereomicroscope at a ×10 magnification with a 1.6× zoom. Ovary size and staging were also assessed, and replicates presenting heterogeneous morphology between sample types were discarded. Tissue for which follow-up processing was performed was transferred into 1.5-ml centrifuge tubes, all excess solution was removed, and tissue was flash-frozen. Four biological replicates of each sample type were shipped overnight to the Purdue Proteomics Facility (Bindley Bioscience Center, Discovery Park, West Lafayette, IN) to be analyzed by a shotgun approach referred to as “discovery-based proteomics” (49–51).

### Sample digestion.

Gel bands were cut into 1-mm pieces and washed to remove the stain with 50:50 acetonitrile (ACN)–25 mM ammonium bicarbonate (ABC) (vol/vol). After washing, the samples were reduced and alkylated. Sequence-grade Lys-C–trypsin (Promega) was used to enzymatically digest the samples. All digestions were carried out with a Barocycler NEP2320 instrument (Pressure BioSciences) at 50°C under 20 kilopounds per square inch for 2 h. Peptides were recovered from gel samples by using 60% ACN–5% trifluoroacetic acid (TFA)–35% purified H_2_O with sonication in an ice bath. The supernatant was removed from the gels, and a vacuum centrifuge was used to dry samples. The resulting pellet was resuspended in 10 μl of 97% purified H_2_O–3% ACN–0.1% formic acid (FA). A 5-μl volume was used for nanoscale liquid chromatography-tandem mass spectrometry (NanoLC-MS/MS) analysis.

### LC-MS/MS analysis.

The samples were analyzed on a Nano Eksigent 425 high-performance liquid chromatography (HPLC) system coupled to a Triple TOF 5600 Plus instrument (ABsciex, Framingham, MA) (52). The gradient was 120 min at 300 nl/min over the cHiPLC-nanoflex system. The trap column was a Nano cHiPLC 200-μm by 0.5-mm ChromXP C_18_-CL 3-μm 120-Å column, followed by the analytical column, a Nano cHiPLC 75-μm by 15-cm ChromXP C_18_-CL 5-μm 120-Å column. The sample was injected into the Triple TOF 5600 Plus column through the Nanospray III source. Data acquisition was performed for 50 precursors at 50 ms/scan. Three technical replicates of this analysis were performed for each sample.

### Proteomic data analysis.

Initial data analysis was performed by using PeakView (ABsciex) and Mascot (Matrix Science) for database searches. D. melanogaster and D. simulans peptide information was compared to information in the respective databases for each host and assigned UniProt identifiers accordingly. All isoform information corresponding to each protein was grouped together for classification as a single protein. To facilitate comparisons of D. melanogaster to D. simulans proteins, each D. simulans protein was assigned the name of its nearest D. melanogaster homolog. Intensity-based absolute quantification (iBAQ) of the protein amount (53) was used as a measure of initial protein detection for each sample type. Label-free quantification (LFQ) was performed by using MaxQuant (54) to identify proteins that satisfied a quality scoring function, enabling comparisons of protein quantity between infection conditions. Both iBAQ and LFQ data were recorded from 4 biological and 3 technical replicates for a combined total of 12 replicates per experimental condition. Proteins designated “reliable” were required to have been detected in 2 out of 3 technical replicates and 3 out of 4 biological replicates according to the LFQ data in order to be included in further data analyses. A coefficient of variation (CV) was also calculated for each significant protein by using the average of LFQ scores from all biological replicates. Only proteins exhibiting a CV below 50% were included in the final list of reliable hits. The reliable proteins were analyzed by using a one-way analysis of variance (ANOVA) approach to identify statistically significant proteins, based upon the LFQ scores of each biological replicate.

Differential protein abundance between sample types was determined by creating pairwise ratios of the average protein LFQ scores for each sample type. For D. melanogaster, differential abundance comparisons were made between the Dmel *w*Mel/Dmel Uninf, Dmel *w*Mel^CS^/Dmel Uninf, and Dmel *w*Mel/Dmel *w*Mel^CS^ strains. For D. simulans, differential abundance comparisons were made between the Dsim *w*Ri/Dsim Cured, Dsim *w*Mel/Dsim Cured, and Dsim *w*Mel/Dsim *w*Ri strains. Proteins that showed an abundance change of >0.58 (log_2_)-fold (equivalent to a 1.5-fold change) were considered to represent differentially abundant proteins. In terms of regular numbers, these thresholds are indicated by a <0.67-fold change or a >1.5-fold change (38).

To assign the significant and differentially abundant proteins to functional classes, we first retrieved sequence information for each UniProt identification (55). An eggNOG v4.5 sequence search was then performed (56) to assign each protein to 1 of 20 possible orthologous groups. The first orthologous group assigned by eggNOG was selected as the initial functional classification for each of the proteins, followed by refinement of certain classifications in consultation with FlyBase and the scientific literature.

### DNA extraction for qPCR.

Wolbachia titers were assessed by qPCR analysis of six biological replicates from each host-strain combination. All flies were prepared as described above, and all sample types were run in parallel for each replicate. In running each replicate, ovary pairs were dissected from 5 females of each sample type. These pairs were homogenized in 200 μl of 0.1 M Tris HCl, 0.1 M EDTA, and 1% SDS (pH 9.0) and incubated for 30 min at 70°C. Twenty microliters of 3 M sodium acetate was added, and samples were mixed by shaking. After incubation for 30 min on ice, the samples were centrifuged at 14,000 rpm for 15 min at 4°C. Two hundred microliters of the supernatant containing DNA was collected, and DNA was precipitated to a final volume of 50 μl by ethanol precipitation. Briefly, 500 μl of absolute ethanol was added to 200 μl of the supernatant. The sample was gently mixed and kept at −20°C for 1 h. After centrifugation of the sample at 14,000 rpm for 15 min at 4°C, the supernatant was removed carefully, and 1 ml of 70% ethanol was added to the pellet. After 1 min, samples were centrifuged again at 14,000 rpm for 15 min at 4°C. After the supernatant was discarded, the DNA pellet was air dried and resuspended in 50 μl of water. These DNA samples were diluted 1:10 for use in qPCR.

### Real-time quantitative PCR analysis.

Real-time PCRs were carried out with a CFX96 real-time PCR detection system (Bio-Rad). Each reaction was performed with a 20-μl final volume containing 10 μl of Maxima SYBR green-fluorescein qPCR master mix (Thermo Scientific), 0.5 μl of 5 mM each primer, and 2 μl of diluted DNA. Primers for the Wolbachia-specific protein (Wsp) gene were used (44). Wsp plasmid standards ranging from 10^2^ to 10^8^ copy numbers were used to generate a standard curve for absolute quantification. The thermal cycling protocol for Wsp amplification involved a 50°C incubation for 2 min and then denaturation for 10 min at 95°C, followed by 40 cycles of 95°C for 30s, 57°C for 1 min, and 72°C for 30 s. Melting curves were examined to confirm the specificity of the amplified product. Data were analyzed by using Bio-Rad CFX manager3.1 with default threshold settings. Absolute Wolbachia copy numbers were obtained by comparing threshold cycle (*C_T_*) values with a standard curve generated from the plasmid standard.

## RESULTS

### Each host-strain combination had ovarian proteins that were reliable in abundance.

To investigate the impact of Wolbachia on maternal transmission, this study focused on analyzing D. melanogaster and D. simulans ovaries of various infection statuses. D. melanogaster stocks that carried the native *w*Mel strain (Dmel *w*Mel) or the virulent *w*Mel^CS^ strain (Dmel *w*Mel^CS^) (44) were derived from the same genetic background as uninfected control flies (Dmel Uninf). D. simulans stocks that carried the native *w*Ri strain (Dsim *w*Ri) or the artificially introduced *w*Mel strain (Dsim *w*Mel) were also generated (46) in the same genetic background as control flies cured with tetracycline (Dsim Cured). The identity of all Wolbachia strains was confirmed with diagnostic PCR assays, sequencing, and quantitative real-time PCR as described previously (43, 44, 57). The use of strain-specific markers confirmed that the D. melanogaster and D. simulans hosts infected with *w*Mel carried the same *w*Mel^*1*^ strain type (Tables 1 and 2). The other infected D. melanogaster line was verified to carry the *w*Mel^CS^ strain and not *w*Mel^CS2^ or *w*Mel^Pop^ variant types (Table 1), based upon the abundance of tandem repeats, the absence of additional Octomom repeats, and the absence of a specific G→A transition found in the *w*Mel^Pop^ strain (44, 58). From this point forward, the confirmed Dmel *w*Mel and Dsim *w*Ri host-strain combinations are collectively referred to as “endogenous,” and the Dmel *w*Mel^CS^ and Dsim *w*Mel combinations are referred to as “variant.”

**TABLE 1:** Genotyping of host-specific Wolbachia variants used in this study^*a*^

Strain	Size of PCR product (kb) (no. of copies)^*b*^				Fragment size of WD0983 (bp)	Presence of G or A at position 943443	Octomom copy no.
	VNTR-105	VNTR-141	IS*5*-WD0516/7	IS*5*-WD1310			
Published variants							
*w*Mel	1.35 (5)	1.33 (7)	2.49 (+)	0.75 (−)	550	G	1
*w*Mel2	1.35 (5)	1.19 (6)	2.49 (+)	0.75 (−)	550	G	1
*w*Mel3	1.35 (5)	1.33 (7)	1.57 (−)	0.75 (−)	550	G	1
*w*Mel^CS^	1.25 (4)	1.19 (6)	1.57 (−)	1.67 (+)	550	G	1
*w*Mel^CS2^	1.35 (5)	1.19 (6)	1.57 (−)	1.67 (+)	550	G	1
*w*Mel^Pop^	1.25 (4)	1.19 (6)	1.57 (−)	1.67 (+)	550	A	Varies (1–15)
Laboratory strains							
Dmel *w*Mel	1.35	1.33	2.49	0.75	550	G	1
Dmel *w*Mel^CS^	1.25	1.19	1.57	1.67	550	G	1
Dsim *w*Mel	1.35	1.33	2.49	0.75	550	G	1
Dsim *w*Ri	ND	ND	ND	ND	ND	ND	0

a Diagnostic VNTR and insertion sequence element (IS*5*) regions were analyzed as described previously (43). The expected product size for a given variant as well as those determined for laboratory strains are listed. Distinguishing criteria for *w*Mel^CS^ and *w*Mel^Pop^, including the G-to-A transition at position 943443 and the Octomom copy number, are also shown (44).

b Presence or absence is represented by + or −, respectively. ND, not detected.

**TABLE 2:** Quantitative PCR of host-specific Wolbachia variants

Gene	Slope	PCR efficiency	Dilution	*C_T_* for indicated host-strain combination			
				Dmel *w*Mel	Dmel *w*Mel^CS^	Dsim *w*Mel	Dsim *w*Ri
Reference *wsp* gene	−3.108	2.0977	1:10	19.15	21.71	21.46	21.55
	−3.108	2.0977	1:100	22.29	25.05	24.48	24.86
Target WD0513 gene	−3.085	2.1094	1:10	19.21	21.97	21.8	32.97
	−3.085	2.1094	1:100	22.26	24.49	24.53	35.25
Fold change relative to control			1:10	1.00	0.85	0.80	0.00
			1:100	1.00	1.46	0.93	0.00
Mean (SE)				1.00 (±0.0)	1.16 (±0.43)	0.87 (±0.09)	0.00 (±0.00)

**FIG 1:**
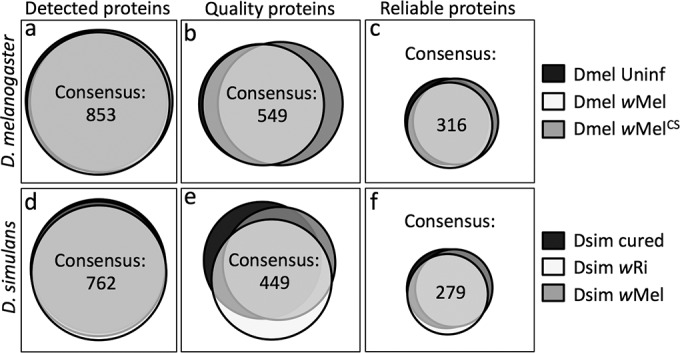
Systematic identification of reliable ovarian proteins shared within each host type. Venn diagrams represent the total number of proteins and the extent of content overlap between each sample type. (a to c) These data are indicated for D. melanogaster at the level of detection (a), quality peptide identification (b), and reliability (c). (d to f) The D. simulans samples had slightly fewer proteins represented overall in the categories of detection (d), quality peptide identification (e), and reliability (f). Proteins detected in Wolbachia-free samples are shown in black. Endogenous Wolbachia-host combinations are shown in white. Variant Wolbachia-host combinations are shown in gray.

To assess the impact of Wolbachia on the Drosophila ovary proteome, ovaries were dissected from all host-strain combinations and analyzed by label-free LC-MS/MS. Four biological replicates were collected for each sample type, and 3 technical replicates were analyzed per sample, for a total of 12 replicates per sample type. This resulted in the initial identification of 927 proteins from the D. melanogaster ovarian samples (see Table S1 in the supplemental material). A total of 853 of these proteins were shared among all host-strain combinations (Fig. 1a). Further analysis determined that 549 of the shared proteins were based upon quality peptides in all D. melanogaster sample types (Fig. 1b; see also Table S1 in the supplemental material). In D. simulans, 834 total proteins were initially identified (see Table S2 in the supplemental material). A total of 762 of these proteins were shared among all D. simulans ovary proteomes analyzed (Fig. 1d). A total of 449 of these shared D. simulans protein identifications were based upon quality peptide information (Fig. 1e; see also Table S2 in the supplemental material). Taken together, these data indicate that 54 to 59% of the protein identifications initially associated with D. simulans and D. melanogaster ovarian proteomes were assigned with high confidence. This set of consensus quality proteins is pursued further in the analyses described below.

The next phase of analysis focused on identifying which consensus quality proteins were reliably detected during oogenesis for each host-strain combination. This required protein detection in at least 2 out of 3 technical replicates per biological sample and at least 3 out of 4 biological samples of each sample type. The coefficient of variation (Gini coefficient) was also calculated for each quality protein hit, and proteins with a CV below 50% were selected as described previously (59). These rigorous criteria defined 316 proteins as being “reliable” among all the D. melanogaster ovarian proteomes analyzed (Fig. 1c; see also Table S1 in the supplemental material). A total of 279 proteins were reliably detected within all ovarian proteomes of D. simulans (Fig. 1f; see also Table S2 in the supplemental material). All quantitative analyses of the ovarian proteomes described below focus on these reliable proteins.

### Most Wolbachia-associated proteomic changes are restricted to a given host type.

To identify ovarian proteins that exhibit significant abundance changes in Wolbachia-infected tissue, comparisons between sample types were performed by using ANOVA. For D. melanogaster, this analysis revealed 61 host proteins whose abundance changed significantly under one or more of the Wolbachia-infected conditions (see Table S3 in the supplemental material) (*P* < 0.05). The Wolbachia surface protein, Wsp, was also identified in Dmel *w*Mel and Dmel *w*Mel^CS^ samples only. For D. simulans, ANOVA identified 49 host proteins that exhibited significantly altered abundance in one or both Wolbachia-infected samples (see Table S4 in the supplemental material) (*P* < 0.05). These ovarian proteins are referred to here as “significant proteins.”

**FIG 2:**
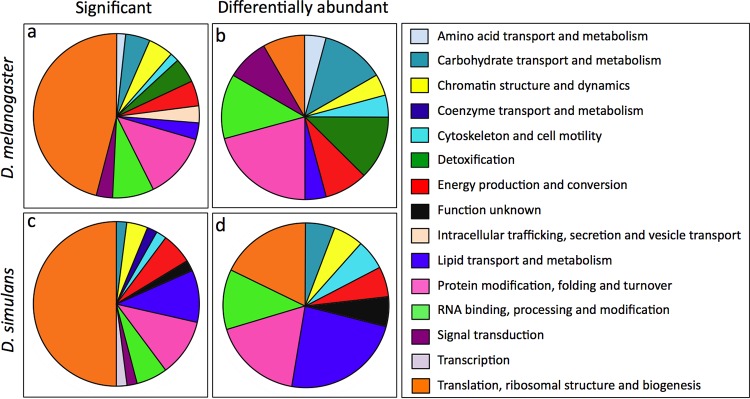
Functional classification of significant and differentially abundant ovarian proteins. The proportional representation of each class is shown for 62 significant D. melanogaster proteins (a), 25 differentially abundant D. melanogaster proteins (b), 49 significant D. simulans proteins (c), and 17 differentially abundant D. simulans proteins (d). Each class is distinguished by a different color, as indicated by the key on the right.

To address the overall functional implications of the group of significant proteins, each protein was assigned to a functional class, based on information from the eggNOG v4.5 program and the Drosophila literature. This analysis grouped the significant proteins into 15 functional classes (Fig. 2a and c). Six functional classes were specific to either D. melanogaster or D. simulans and represented ≤10% of the total proteins. The remaining 9 functional classes were shared between host types. Translation-related proteins were highly represented, comprising up to half of the significant proteins overall. The other shared functional classes were carbohydrate transport and metabolism; chromatin structure and dynamics; cytoskeleton and cell motility; energy conversion; lipid transport and metabolism; protein modification, folding, and turnover; RNA binding, processing, and modification; and signal transduction (Fig. 2a and c). This implicates a diverse subset of ovarian cellular processes as being responsive to Wolbachia.

The similarity of D. melanogaster and D. simulans ovarian responses to Wolbachia was further assessed in terms of overlap between consensus significant proteins. As D. simulans annotation is less extensive than that of D. melanogaster, all D. simulans proteins were named as per the closest D. melanogaster homologs to facilitate this comparison. Out of 95 total significant proteins, this analysis identified 15 significant proteins as being shared between D. melanogaster and D. simulans ovarian proteomes (see Tables S3 and S4 in the supplemental material). These proteins were glycogen phosphorylase, the ATP synthase delta subunit, retinoid- and fatty acid-binding glycoprotein, heat shock proteins 26 and 27, the hnRNP protein Squid, and 9 different ribosomal proteins (see Tables S3 and S4 in the supplemental material). Thus, a limited redundancy of individual proteins was evident among the significant ovarian proteins of D. melanogaster and D. simulans.

To assess the putative functional impact of Wolbachia-responsive significant proteins, the magnitude of protein abundance changes was examined. As in previous proteomics studies, differential abundance on the order of a ≥1.5-fold change is predicted to indicate functional upregulation. Conversely, a ≤0.667-fold change is predicted to indicate functional downregulation (38, 60–62). Comparisons of the significant protein data yielded 25 differentially abundant proteins in D. melanogaster, representing 11 functional classes (Fig. 2b and Table 3). Seventeen differentially abundant proteins were detected in D. simulans, comprised of 9 functional classes (Fig. 2d and Table 4). The 8 classes of differentially abundant proteins shared between host types were carbohydrate transport and metabolism; chromatin structure and dynamics; cytoskeleton and cell motility; energy production and conversion; lipid transport and metabolism; protein modification, folding, and turnover; RNA binding, processing, and modification; and translation, ribosomal structure, and biogenesis (Fig. 2b and d). This suggests that the differentially abundant proteins represent a distinct subset of significant proteins. The differential abundance data also indicated that the composition of each shared functional class is largely organism specific. The few differentially abundant proteins that were shared between hosts were glycogen phosphorylase, the ATP synthase delta subunit, and heat shock proteins 26 and 27.

**TABLE 3:** Differentially abundant proteins identified through comparison of D. melanogaster ovarian proteomes^*a*^

Functional classification	Protein	Relative abundance		
		*w*Mel/Uninf	*w*Mel^CS^/Uninf	*w*Mel^CS^/wMel
Amino acid transport and metabolism	Eip55E	1.271	**1.587**	1.248
Carbohydrate transport and metabolism	Aldolase	0.919	**0.630**	0.685
	Glycogen phosphorylase	0.839	1.447	**1.724**
	Succinyl coenzyme A synthetase α subunit	NA	NA	**0.591**
Chromatin structure and dynamics	Vig2	0.868	1.330	**1.533**
Cytoskeleton and cell motility	Ciboulot	0.858	1.380	**1.608**
Detoxification	Glutathione *S*-transferase D1	1.028	**1.626**	**1.581**
	Peroxinectin-like	0.889	**1.704**	**1.917**
	Transferrin 1	**2.000**	NA	NA
Energy production and conversion	ATP synthase, δ subunit	0.888	1.367	**1.540**
	Isocitrate dehydrogenase	0.776	1.314	**1.693**
Lipid transport and metabolism	Jabba	0.801	1.298	**1.621**
Protein modification, folding, and turnover	Cysteine proteinase 1	1.042	**1.603**	**1.539**
	Heat shock protein 26	0.951	**1.558**	**1.639**
	Heat shock protein 27	1.087	**1.525**	1.403
	Hsc/Hsp70-interacting protein related	0.828	1.266	**1.529**
	Regulatory particle non-ATPase 6	1.098	**1.578**	1.437
RNA binding, processing, and modification	Hoi-polloi	1.214	0.795	**0.655**
	Modulo	1.035	0.687	**0.663**
	Rm62	0.999	**0.644**	**0.644**
Signal transduction	14-3-3ζ	**0.611**	**0.555**	0.909
	Terribly reduced optic lobes	1.154	**0.647**	**0.561**
Translation, ribosomal structure, and biogenesis	Ribosomal protein S27	0.989	**0.645**	**0.652**
	Seryl-tRNA synthetase	**0.618**	NA	NA
Wolbachia protein	Wolbachia surface protein	NA	NA	**0.632**

a Relative abundance represents a ratio of average LFQ scores for each sample type: Dmel *w*Mel/Dmel Uninf, Dmel *w*Mel^CS^/Dmel Uninf, and Dmel *w*Mel^CS^/Dmel *w*Mel. Ratios indicating protein up- or downregulation are shown in boldface type. NA, not applicable.

**TABLE 4:** Differentially abundant proteins identified through comparison of D. simulans ovarian proteomes^*a*^

Functional classification	Protein	Relative abundance		
		*w*Ri/Cured	*w*Mel/Cured	*w*Mel/*w*Ri
Carbohydrate transport and metabolism	Glycogen phosphorylase	1.097	**1.884**	**1.717**
Chromatin structure and dynamics	Histone H4	0.913	**0.645**	0.706
Cytoskeleton and cell motility	Tropomyosin 2	1.081	**1.701**	**1.573**
Energy production and conversion	ATP synthase, δ subunit	**1.687**	1.120	**0.664**
Function unknown	Female-specific independent of transformer	0.785	**3.359**	**4.277**
Lipid transport and metabolism	CG3902 (acyl-CoA dehydrogenase activity)	**1.651**	**1.664**	0.974
	Retinoid- and fatty acid-binding glycoprotein	**2.175**	**2.385**	1.008
	Yolk protein 1	1.407	**1.990**	1.414
	Yolk protein 2	1.400	**1.693**	1.209
Protein modification, folding, and turnover	Heat shock protein 26	1.193	**1.753**	1.469
	Heat shock protein 27	**0.631**	1.299	**2.058**
	Tripeptidyl-peptidase II	0.701	1.230	**1.755**
RNA binding, processing, and modification	Fibrillarin	1.089	**0.488**	**0.448**
	Squid	**0.569**	0.677	1.191
Translation, ribosomal structure, and biogenesis	Ribosomal protein L32	0.957	**0.651**	**0.654**
	Ribosomal protein L34b	**0.654**	**0.593**	0.906
	Ribosomal protein S25	0.962	**0.629**	0.680

a Relative abundance represents a ratio of average LFQ scores for each sample type: Dsim *w*Ri/Dsim Cured, Dsim *w*Mel/Dsim Cured, and Dsim *w*Mel/Dsim *w*Ri. Ratios that indicate up- or downregulation are indicated in boldface type. CoA, coenzyme A.

### Differential protein abundance patterns associated with host and Wolbachia types.

To further define the impact of specific Wolbachia strains on the host ovary proteome, host-strain combinations were examined in terms of the commonalities that they share. One issue was to determine the extent of overlap between proteomic responses to endogenous and variant Wolbachia infections. In D. melanogaster, comparison of Dmel *w*Mel to Dmel Uninf yielded 3 differentially abundant proteins, whereas comparison of Dmel *w*Mel^CS^ to Dmel Uninf yielded 12 (Table 3). In D. simulans, comparison of Dsim *w*Ri and Dsim Cured revealed 6 differentially abundant proteins, while comparison of Dsim *w*Mel to Dmel Cured identified 13 (Table 4). This suggests that infections by variant Wolbachia strains had a more robust impact than infections by endogenous Wolbachia strains on ovarian proteomic responses at the level of differential abundance.

Another issue to address was the extent of bacterial versus host influence on the ovarian proteomic responses to Wolbachia. To assess the consistency of responses associated with a single Wolbachia strain, ovarian responses to *w*Mel were tracked across host types. This analysis indicated that distinctive proteomic responses were evident in the natural D. melanogaster host compared to the ectopic D. simulans host (Tables 3 and 4). The similarity of host responses to multiple Wolbachia strains was also investigated. Direct comparison of Dmel *w*Mel^CS^ to Dmel *w*Mel identified 11 additional differentially abundant proteins, including the Wolbachia surface protein (Wsp) (Table 3). Most of these hits were due to oppositely directed protein abundance shifts under each Wolbachia infection condition. Direct comparison of Dsim *w*Mel to Dmel *w*Ri identified 8 differentially abundant proteins as well. However, nearly all these shifts were redundant with shifts already identified in comparisons between infected and uninfected D. simulans ovaries (Table 4). This suggests that ovarian proteomic responses to different Wolbachia strains were milder and more diversified in D. melanogaster than in D. simulans, where all-or-nothing responses were predominant.

Previous studies showed that high-titer Wolbachia infections exert the most extensive impact on host physiological processes (44, 63–65). This precedent raises questions about the role of Wolbachia titer in specifying Wolbachia-associated changes in the ovary proteome. Real-time quantitative PCR was performed to assess ovarian Wolbachia abundance. The data indicated that Dmel *w*Mel^CS^ ovaries carried only 51% of the Wolbachia titer detected in Dmel *w*Mel ovaries (*P* = 0.047) (*n* = 60 ovaries per condition) (see Fig. S1 in the supplemental material). Ovarian Wolbachia titers detected in Dsim *w*Ri and Dsim *w*Mel ovaries were not significantly different from each other or from those in Dmel *w*Mel ovaries (see Fig. S1 in the supplemental material). This does not support a role for elevated Wolbachia titers as a determinant of ovarian proteomic responses but alternatively favors consideration of molecular and cellular mechanisms intrinsic to each scenario.

## DISCUSSION

In applying a proteomic approach to ovarian responses to Wolbachia, a central consideration is whether the data set substantiates current knowledge of infection. Based upon previous work, one expectation is that variant host-strain combinations should exhibit stress indicators (36, 40, 66, 67). Notably, the variant Dmel *w*Mel^CS^ and Dsim *w*Mel combinations in this study exhibited depletion of dozens of ribosomal constituents, consistent with overall downregulation (68–70). Upregulation of heat shock and detoxification proteins was also seen, consistent with a stress response (71–73). Ovaries from the Dsim *w*Mel combination have also been shown to exhibit extensive chromatin structuring defects in nurse cells, analogous to *squid* mutant organisms (16, 74). The downregulation of the Squid protein observed here informs the basis for this response.

Another expectation is that Wolbachia should strategically enhance ovarian survival and proliferation mechanisms to maximize transmission. The findings of this study corroborate the involvement of known factors while also identifying new candidate contributors. The upregulation of the iron-sequestering protein transferrin 1 is in agreement with previous reports that Wolbachia bacteria protect the germ line from iron-associated toxicity (25–28). An increased abundance of the retinoid- and fatty acid-binding protein, indicated to have heme-binding activity, may help to protect the germ line from oxidative stress as well (75). Upregulation of the Sxl effector protein, Female-specific independent of transformer, opens a speculative route for Wolbachia modulation of *Sxl*-induced germ line lethality (29, 76, 77). The downregulation of the cell division suppressor 14-3-3 zeta is also consistent with enhanced germ line stem cell division rates observed for Wolbachia-infected organisms (11, 78).

It is further expected that Wolbachia bacteria drive modifications of the ovarian environment that support Wolbachia persistence. Some evidence from this study supports that prediction. From a nutritional standpoint, an elevated abundance of proteases and proteasome subunits is consistent with the possibility of increased amino acid availability for Wolbachia (79). The upregulation of glycogen phosphorylase complements recent work using Brugia malayi nematodes, which indicated that Wolbachia bacteria induce the upregulation of glycolytic enzymes (80). An increased local availability of pyruvate is hypothesized to benefit Wolbachia (81). It was also recently shown that filamentous actin is important for stabilizing Wolbachia colonization of the host germ line (21). Wolbachia-associated upregulation of tropomyosin, a microfilament-stabilizing protein, is consistent with that model (82). Taken together, these data support the study outcomes as being representative while also associating the potential use of these transmission-enhancing mechanisms with new host-strain combinations.

It is notable that very few proteins were detected as being significant or differentially abundant across all sample types analyzed in this study. Data sets from previous Wolbachia-omics studies exhibit a wide range of Wolbachia-responsive host expression changes, indicating that contextual influences are substantial (31–42) (see Table S5 in the supplemental material). Analagous to those prior studies, our study provides substantial evidence of context-dependent responses to Wolbachia infection. Infections with endogenous Wolbachia strains had little effect on the host proteome compared to infections with variant Wolbachia strains, in agreement with data from previous work on heterologous symbiont infections of cnidarians (83). Ovarian proteomic responses to Wolbachia also correlated poorly with Wolbachia titers, paralleling results from a previous fecundity study (84). This argues against the conservation of Wolbachia-ovary interactions in terms of specific protein abundance shifts. A combination of effects may contribute to this outcome, including technical limitations of the assay (51) as well as Wolbachia adaptation (85), modification of Wolbachia population structure (85), and/or selection (86). Regardless, the finite physical constraints of transmission inherently favor Wolbachia manipulation of the most functionally advantageous processes. Context-specific regulation of consensus ovarian mechanisms may contribute substantially to the achievement of this goal.

## Supplementary Material

Supplemental material

## References

[1] Werren JH 1997 Biology of Wolbachia. Annu Rev Entomol 42:587–609. doi:10.1146/annurev.ento.42.1.587. 15012323

[2] Bouchon D, Rigaud T, Juchault P 1998 Evidence for widespread Wolbachia infection in isopod crustaceans: molecular identification and host feminization. Proc Biol Sci 265:1081–1090. doi:10.1098/rspb.1998.0402. 9684374 PMC1689171

[3] Casiraghi M, Anderson T, Bandi C, Bazzocchi C, Genchi C 2001 A phylogenetic analysis of filarial nematodes: comparison with the phylogeny of Wolbachia endosymbionts. Parasitology 122:93–103. doi:10.1017/S0031182000007149. 11197770

[4] Zug R, Hammerstein P 2012 Still a host of hosts for Wolbachia: analysis of recent data suggests that 40% of terrestrial arthropod species are infected. PLoS One 7:e38544. doi:10.1371/journal.pone.0038544. 22685581 PMC3369835

[5] O'Neill SL, Hoffmann AA, Werren JH 1997 Influential passengers: inherited microorganisms and arthropod reproduction. Oxford University Press, Oxford, United Kingdom.

[6] Stouthamer R, Breeuwer JA, Hurst GD 1999 Wolbachia pipientis: microbial manipulator of arthropod reproduction. Annu Rev Microbiol 53:71–102. doi:10.1146/annurev.micro.53.1.71. 10547686

[7] Dobson SL, Bourtzis K, Braig HR, Jones BF, Zhou W, Rousset F, O'Neill SL 1999 Wolbachia infections are distributed throughout insect somatic and germ line tissues. Insect Biochem Mol Biol 29:153–160. doi:10.1016/S0965-1748(98)00119-2. 10196738

[8] King RC 1970 Ovarian development in Drosophila melanogaster. Academic Press, New York, NY.

[9] Frydman HM, Li JM, Robson DN, Wieschaus E 2006 Somatic stem cell niche tropism in Wolbachia. Nature 441:509–512. doi:10.1038/nature04756. 16724067

[10] Serbus LR, Casper-Lindley C, Landmann F, Sullivan W 2008 The genetics and cell biology of Wolbachia-host interactions. Annu Rev Genet 42:683–707. doi:10.1146/annurev.genet.41.110306.130354. 18713031

[11] Fast EM, Toomey ME, Panaram K, Desjardins D, Kolaczyk ED, Frydman HM 2011 Wolbachia enhance Drosophila stem cell proliferation and target the germline stem cell niche. Science 334:990–992. doi:10.1126/science.1209609. 22021671 PMC4030408

[12] Genty LM, Bouchon D, Raimond M, Bertaux J 2014 Wolbachia infect ovaries in the course of their maturation: last minute passengers and priority travellers? PLoS One 9:e94577. doi:10.1371/journal.pone.0094577. 24722673 PMC3983217

[13] Toomey ME, Panaram K, Fast EM, Beatty C, Frydman HM 2013 Evolutionarily conserved Wolbachia-encoded factors control pattern of stem-cell niche tropism in Drosophila ovaries and favor infection. Proc Natl Acad Sci U S A 110:10788–10793. doi:10.1073/pnas.1301524110. 23744038 PMC3696799

[14] Casper-Lindley C, Kimura S, Saxton DS, Essaw Y, Simpson I, Tan V, Sullivan W 2011 Rapid fluorescence-based screening for Wolbachia endosymbionts in Drosophila germ line and somatic tissues. Appl Environ Microbiol 77:4788–4794. doi:10.1128/AEM.00215-11. 21622788 PMC3147364

[15] McGraw EA, Merritt DJ, Droller JN, O'Neill SL 2002 Wolbachia density and virulence attenuation after transfer into a novel host. Proc Natl Acad Sci U S A 99:2918–2923. doi:10.1073/pnas.052466499. 11880639 PMC122448

[16] Serbus L, Ferreccio A, Zhukova M, McMorris C, Kiseleva E, Sullivan W 2011 A feedback loop between Wolbachia and the Drosophila gurken mRNP complex influences Wolbachia titer. J Cell Sci 124:4299–4308. doi:10.1242/jcs.092510. 22193955 PMC3258112

[17] Serbus LR, Sullivan W 2007 A cellular basis for Wolbachia recruitment to the host germline. PLoS Pathog 3:e190. doi:10.1371/journal.ppat.0030190. 18085821 PMC2134955

[18] Veneti Z, Clark ME, Karr TL, Savakis C, Bourtzis K 2004 Heads or tails: host-parasite interactions in the Drosophila-Wolbachia system. Appl Environ Microbiol 70:5366–5372. doi:10.1128/AEM.70.9.5366-5372.2004. 15345422 PMC520876

[19] Hadfield SJ, Axton JM 1999 Germ cells colonized by endosymbiotic bacteria. Nature 402:482. doi:10.1038/45002. 10591206

[20] Kose H, Karr TL 1995 Organization of Wolbachia pipientis in the Drosophila fertilized egg and embryo revealed by an anti-Wolbachia monoclonal antibody. Mech Dev 51:275–288. doi:10.1016/0925-4773(95)00372-X. 7547474

[21] Newton IL, Savytskyy O, Sheehan KB 2015 Wolbachia utilize host actin for efficient maternal transmission in Drosophila melanogaster. PLoS Pathog 11:e1004798. doi:10.1371/journal.ppat.1004798. 25906062 PMC4408098

[22] Kriesner P, Hoffmann AA, Lee SF, Turelli M, Weeks AR 2013 Rapid sequential spread of two Wolbachia variants in Drosophila simulans. PLoS Pathog 9:e1003607. doi:10.1371/journal.ppat.1003607. 24068927 PMC3771877

[23] Weeks AR, Turelli M, Harcombe WR, Reynolds KT, Hoffmann AA 2007 From parasite to mutualist: rapid evolution of Wolbachia in natural populations of Drosophila. PLoS Biol 5:e114. doi:10.1371/journal.pbio.0050114. 17439303 PMC1852586

[24] Ikeya T, Broughton S, Alic N, Grandison R, Partridge L 2009 The endosymbiont Wolbachia increases insulin/IGF-like signalling in Drosophila. Proc Biol Sci 276:3799–3807. doi:10.1098/rspb.2009.0778. 19692410 PMC2817276

[25] Kremer N, Voronin D, Charif D, Mavingui P, Mollereau B, Vavre F 2009 Wolbachia interferes with ferritin expression and iron metabolism in insects. PLoS Pathog 5:e1000630. doi:10.1371/journal.ppat.1000630. 19851452 PMC2759286

[26] Dedeine F, Bouletreau M, Vavre F 2005 Wolbachia requirement for oogenesis: occurrence within the genus Asobara (Hymenoptera, Braconidae) and evidence for intraspecific variation in A. tabida. Heredity 95:394–400. doi:10.1038/sj.hdy.6800739. 16118660

[27] Brownlie JC, Cass BN, Riegler M, Witsenburg JJ, Iturbe-Ormaetxe I, McGraw EA, O'Neill SL 2009 Evidence for metabolic provisioning by a common invertebrate endosymbiont, Wolbachia pipientis, during periods of nutritional stress. PLoS Pathog 5:e1000368. doi:10.1371/journal.ppat.1000368. 19343208 PMC2657209

[28] Pannebakker BA, Loppin B, Elemans CP, Humblot L, Vavre F 2007 Parasitic inhibition of cell death facilitates symbiosis. Proc Natl Acad Sci U S A 104:213–215. doi:10.1073/pnas.0607845104. 17190825 PMC1765438

[29] Starr DJ, Cline TW 2002 A host parasite interaction rescues Drosophila oogenesis defects. Nature 418:76–79. doi:10.1038/nature00843. 12097909

[30] Landmann F, Voronin D, Sullivan W, Taylor MJ 2011 Anti-filarial activity of antibiotic therapy is due to extensive apoptosis after Wolbachia depletion from filarial nematodes. PLoS Pathog 7:e1002351. doi:10.1371/journal.ppat.1002351. 22072969 PMC3207916

[31] Kremer N, Charif D, Henri H, Gavory F, Wincker P, Mavingui P, Vavre F 2012 Influence of Wolbachia on host gene expression in an obligatory symbiosis. BMC Microbiol 12:1. doi:10.1186/1471-2180-12-1. 22376153 PMC3287518

[32] Zheng Y, Wang J-L, Liu C, Wang C-P, Walker T, Wang Y-F 2011 Differentially expressed profiles in the larval testes of Wolbachia infected and uninfected Drosophila. BMC Genomics 12:1. doi:10.1186/1471-2164-12-1. PMC3261232 22145623

[33] Chevalier F, Herbiniere-Gaboreau J, Charif D, Mitta G, Gavory F, Wincker P, Greve P, Braquart-Varnier C, Bouchon D 2012 Feminizing Wolbachia: a transcriptomics approach with insights on the immune response genes in Armadillidium vulgare. BMC Microbiol 12(Suppl 1):S1. doi:10.1186/1471-2180-12-S1-S1. 22375708 PMC3287506

[34] Hughes GL, Ren X, Ramirez JL, Sakamoto JM, Bailey JA, Jedlicka AE, Rasgon JL 2011 Wolbachia infections in Anopheles gambiae cells: transcriptomic characterization of a novel host-symbiont interaction. PLoS Pathog 7:e1001296. doi:10.1371/journal.ppat.1001296. 21379333 PMC3040664

[35] Rao RU, Huang Y, Abubucker S, Heinz M, Crosby SD, Mitreva M, Weil GJ 2012 Effects of doxycycline on gene expression in Wolbachia and Brugia malayi adult female worms in vivo. J Biomed Sci 19:21. doi:10.1186/1423-0127-19-21. 22321609 PMC3352068

[36] Pan X, Zhou G, Wu J, Bian G, Lu P, Raikhel AS, Xi Z 2012 Wolbachia induces reactive oxygen species (ROS)-dependent activation of the Toll pathway to control dengue virus in the mosquito Aedes aegypti. Proc Natl Acad Sci U S A 109:E23–E31. doi:10.1073/pnas.1116932108. 22123956 PMC3252928

[37] Xi Z, Gavotte L, Xie Y, Dobson SL 2008 Genome-wide analysis of the interaction between the endosymbiotic bacterium Wolbachia and its Drosophila host. BMC Genomics 9:1. doi:10.1186/1471-2164-9-1. 18171476 PMC2253531

[38] Yuan LL, Chen X, Zong Q, Zhao T, Wang JL, Zheng Y, Zhang M, Wang Z, Brownlie JC, Yang F, Wang YF 2015 Quantitative proteomic analyses of molecular mechanisms associated with cytoplasmic incompatibility in Drosophila melanogaster induced by Wolbachia. J Proteome Res 14:3835–3847. doi:10.1021/acs.jproteome.5b00191. 26220534

[39] Zhang YK, Ding XL, Rong X, Hong XY 2015 How do hosts react to endosymbionts. A new insight into the molecular mechanisms underlying the Wolbachia-host association. Insect Mol Biol 24:1–12. doi:10.1111/imb.12128. 25224730

[40] Brennan LJ, Keddie BA, Braig HR, Harris HL 2008 The endosymbiont Wolbachia pipientis induces the expression of host antioxidant proteins in an Aedes albopictus cell line. PLoS One 3:e2083. doi:10.1371/journal.pone.0002083. 18461124 PMC2324199

[41] Dangi A, Vedi S, Nag JK, Paithankar S, Singh MP, Kar SK, Dube A, Misra-Bhattacharya S 2009 Tetracycline treatment targeting Wolbachia affects expression of an array of proteins in Brugia malayi parasite. Proteomics 9:4192–4208. doi:10.1002/pmic.200800324. 19722191

[42] Sun S, Cline TW 2009 Effects of Wolbachia infection and ovarian tumor mutations on sex-lethal germline functioning in Drosophila. Genetics 181:1291–1301. doi:10.1534/genetics.108.099374. 19171941 PMC2666500

[43] Riegler M, Sidhu M, Miller WJ, O'Neill SL 2005 Evidence for a global Wolbachia replacement in Drosophila melanogaster. Curr Biol 15:1428–1433. doi:10.1016/j.cub.2005.06.069. 16085497

[44] Chrostek E, Marialva MSP, Esteves SS, Weinert LA, Martinez J, Jiggins FM, Teixeira L 2013 Wolbachia variants induce differential protection to viruses in Drosophila melanogaster: a phenotypic and phylogenomic analysis. PLoS Genet 9:e1003896. doi:10.1371/journal.pgen.1003896. 24348259 PMC3861217

[45] Pfaffl MW 2001 A new mathematical model for relative quantification in real-time RT-PCR. Nucleic Acids Res 29:e45. doi:10.1093/nar/29.9.e45. 11328886 PMC55695

[46] Poinsot D, Bourtzis K, Markakis G, Savakis C, Mercot H 1998 Wolbachia transfer from Drosophila melanogaster into D. simulans: host effect and cytoplasmic incompatibility relationships. Genetics 150:227–237. 9725842 10.1093/genetics/150.1.227 PMC1460311

[47] Bloomington Drosophila Stock Center. 2007 Standard fly medium recipe. Bloomington Drosophila Stock Center, Bloomington, IN http://flystocks.bio.indiana.edu/Fly_Work/media-recipes/bloomfood.htm Accessed 9 March 2016.

[48] Serbus LR, White PM, Silva JP, Rabe A, Teixeira L, Albertson R, Sullivan W 2015 The impact of host diet on Wolbachia titer in Drosophila. PLoS Pathog 11:e1004777. doi:10.1371/journal.ppat.1004777. 25826386 PMC4380406

[49] Doerr A 29 9 2014 Mass spectrometry-based proteomics. Methagora. http://blogs.nature.com/methagora/2014/09/mass-spectrometry-based-proteomics-at-nature-methods.html.

[50] Bantscheff M, Lemeer S, Savitski MM, Kuster B 2012 Quantitative mass spectrometry in proteomics: critical review update from 2007 to the present. Anal Bioanal Chem 404:939–965. doi:10.1007/s00216-012-6203-4. 22772140

[51] Patterson SD 2004 How much of the proteome do we see with discovery-based proteomics methods and how much do we need to see? Curr Proteomics 1:3–12. doi:10.2174/1570164043488306.

[52] Andrews GL, Simons BL, Young JB, Hawkridge AM, Muddiman DC 2011 Performance characteristics of a new hybrid quadrupole time-of-flight tandem mass spectrometer (TripleTOF 5600). Anal Chem 83:5442–5446. doi:10.1021/ac200812d. 21619048 PMC3138073

[53] Schwanhäusser B, Busse D, Li N, Dittmar G, Schuchhardt J, Wolf J, Chen W, Selbach M 2011 Global quantification of mammalian gene expression control. Nature 473:337–342. doi:10.1038/nature10098. 21593866

[54] Cox J, Mann M 2008 MaxQuant enables high peptide identification rates, individualized ppb-range mass accuracies and proteome-wide protein quantification. Nat Biotechnol 26:1367–1372. doi:10.1038/nbt.1511. 19029910

[55] Pundir S, Magrane M, Martin MJ, O'Donovan C, UniProt Consortium 2015 Searching and navigating UniProt databases. Curr Protoc Bioinformatics 50:1.27.1–1.27.10. doi:10.1002/0471250953.bi0127s50. 26088053 PMC4522465

[56] Huerta-Cepas J, Szklarczyk D, Forslund K, Cook H, Heller D, Walter MC, Rattei T, Mende DR, Sunagawa S, Kuhn M, Jensen LJ, von Mering C, Bork P 2016 eggNOG 4.5: a hierarchical orthology framework with improved functional annotations for eukaryotic, prokaryotic and viral sequences. Nucleic Acids Res 44:D286–D293. doi:10.1093/nar/gkv1248. 26582926 PMC4702882

[57] Chrostek E, Teixeira L 2015 Mutualism breakdown by amplification of Wolbachia genes. PLoS Biol 13:e1002065. doi:10.1371/journal.pbio.1002065. 25668031 PMC4323108

[58] Woolfit M, Iturbe-Ormaetxe I, Brownlie JC, Walker T, Riegler M, Seleznev A, Popovici J, Rancès E, Wee BA, Pavlides J 2013 Genomic evolution of the pathogenic Wolbachia strain, *w*MelPop. Genome Biol Evol 5:2189–2204. doi:10.1093/gbe/evt169. 24190075 PMC3845649

[59] Maes E, Landuyt B, Mertens I, Schoofs L 2013 Interindividual variation in the proteome of human peripheral blood mononuclear cells. PLoS One 8:e61933. doi:10.1371/journal.pone.0061933. 23613975 PMC3629925

[60] Blagoev B, Ong S-E, Kratchmarova I, Mann M 2004 Temporal analysis of phosphotyrosine-dependent signaling networks by quantitative proteomics. Nat Biotechnol 22:1139–1145. doi:10.1038/nbt1005. 15314609

[61] Grønborg M, Kristiansen TZ, Iwahori A, Chang R, Reddy R, Sato N, Molina H, Jensen ON, Hruban RH, Goggins MG 2006 Biomarker discovery from pancreatic cancer secretome using a differential proteomic approach. Mol Cell Proteomics 5:157–171. 16215274 10.1074/mcp.M500178-MCP200

[62] Lewis TS, Hunt JB, Aveline LD, Jonscher KR, Louie DF, Yeh JM, Nahreini TS, Resing KA, Ahn NG 2000 Identification of novel MAP kinase pathway signaling targets by functional proteomics and mass spectrometry. Mol Cell 6:1343–1354. doi:10.1016/S1097-2765(00)00132-5. 11163208

[63] Unckless RL, Boelio LM, Herren JK, Jaenike J 2009 Wolbachia as populations within individual insects: causes and consequences of density variation in natural populations. Proc Biol Sci 276:2805–2811. doi:10.1098/rspb.2009.0287. 19419989 PMC2839946

[64] Clark ME, Veneti Z, Bourtzis K, Karr TL 2003 Wolbachia distribution and cytoplasmic incompatibility during sperm development: the cyst as the basic cellular unit of CI expression. Mech Dev 120:185–198. doi:10.1016/S0925-4773(02)00424-0. 12559491

[65] Osborne SE, Iturbe-Ormaetxe I, Brownlie JC, O'Neill SL, Johnson KN 2012 Antiviral protection and the importance of Wolbachia density and tissue tropism in Drosophila simulans. Appl Environ Microbiol 78:6922–6929. doi:10.1128/AEM.01727-12. 22843518 PMC3457512

[66] Zug R, Hammerstein P 2015 Wolbachia and the insect immune system: what reactive oxygen species can tell us about the mechanisms of Wolbachia-host interactions. Front Microbiol 6:1201. doi:10.3389/fmicb.2015.01201. 26579107 PMC4621438

[67] Wong ZS, Brownlie JC, Johnson KN 2015 Oxidative stress correlates with Wolbachia-mediated antiviral protection in Wolbachia-Drosophila associations. Appl Environ Microbiol 81:3001–3005. doi:10.1128/AEM.03847-14. 25710364 PMC4393424

[68] Lackner DH, Schmidt MW, Wu S, Wolf DA, Bahler J 2012 Regulation of transcriptome, translation, and proteome in response to environmental stress in fission yeast. Genome Biol 13:R25. doi:10.1186/gb-2012-13-4-r25. 22512868 PMC3446299

[69] Hayashi Y, Kuroda T, Kishimoto H, Wang C, Iwama A, Kimura K 2014 Downregulation of rRNA transcription triggers cell differentiation. PLoS One 9:e98586. doi:10.1371/journal.pone.0098586. 24879416 PMC4039485

[70] Boulon S, Westman BJ, Hutten S, Boisvert F-M, Lamond AI 2010 The nucleolus under stress. Mol Cell 40:216–227. doi:10.1016/j.molcel.2010.09.024. 20965417 PMC2987465

[71] Ye ZW, Zhang J, Townsend DM, Tew KD 2015 Oxidative stress, redox regulation and diseases of cellular differentiation. Biochim Biophys Acta 1850:1607–1621. doi:10.1016/j.bbagen.2014.11.010. 25445706 PMC4433447

[72] Wang HD, Kazemi-Esfarjani P, Benzer S 2004 Multiple-stress analysis for isolation of Drosophila longevity genes. Proc Natl Acad Sci U S A 101:12610–12615. doi:10.1073/pnas.0404648101. 15308776 PMC515105

[73] Johansson MW, Holmblad T, Thornqvist PO, Cammarata M, Parrinello N, Soderhall K 1999 A cell-surface superoxide dismutase is a binding protein for peroxinectin, a cell-adhesive peroxidase in crayfish. J Cell Sci 112(Part 6):917–925. 10036241 10.1242/jcs.112.6.917

[74] Goodrich JS, Clouse KN, Schupbach T 2004 Hrb27C, Sqd and Otu cooperatively regulate gurken RNA localization and mediate nurse cell chromosome dispersion in Drosophila oogenesis. Development 131:1949–1958. doi:10.1242/dev.01078. 15056611

[75] Duncan T, Osawa Y, Kutty RK, Kutty G, Wiggert B 1999 Heme-binding by Drosophila retinoid- and fatty acid-binding glycoprotein (RFABG), a member of the proapolipophorin gene family. J Lipid Res 40:1222–1228. 10393207

[76] Evans DS, Cline TW 2013 Drosophila switch gene Sex-lethal can bypass its switch-gene target transformer to regulate aspects of female behavior. Proc Natl Acad Sci U S A 110:E4474–E4481. doi:10.1073/pnas.1319063110. 24191002 PMC3839706

[77] Fujii S, Amrein H 2002 Genes expressed in the Drosophila head reveal a role for fat cells in sex-specific physiology. EMBO J 21:5353–5363. doi:10.1093/emboj/cdf556. 12374736 PMC129088

[78] Su TT, Parry DH, Donahoe B, Chien CT, O'Farrell PH, Purdy A 2001 Cell cycle roles for two 14-3-3 proteins during Drosophila development. J Cell Sci 114:3445–3454. 11682604 10.1242/jcs.114.19.3445 PMC2754241

[79] Fallon AM, Witthuhn BA 2009 Proteasome activity in a naive mosquito cell line infected with Wolbachia pipientis wAlbB. In Vitro Cell Dev Biol Anim 45:460–466. doi:10.1007/s11626-009-9193-6. 19296184 PMC2732765

[80] Voronin D, Bachu S, Shlossman M, Unnasch TR, Ghedin E, Lustigman S 2016 Glucose and glycogen metabolism in Brugia malayi is associated with Wolbachia symbiont fitness. PLoS One 11:e0153812. doi:10.1371/journal.pone.0153812. 27078260 PMC4831766

[81] Melnikow E, Xu S, Liu J, Bell AJ, Ghedin E, Unnasch TR, Lustigman S 2013 A potential role for the interaction of Wolbachia surface proteins with the Brugia malayi glycolytic enzymes and cytoskeleton in maintenance of endosymbiosis. PLoS Negl Trop Dis 7:e2151. doi:10.1371/journal.pntd.0002151. 23593519 PMC3617236

[82] Cooper JA 2002 Actin dynamics: tropomyosin provides stability. Curr Biol 12:R523–R525. doi:10.1016/S0960-9822(02)01028-X. 12176375

[83] Meyer E, Weis VM 2012 Study of cnidarian-algal symbiosis in the “omics” age. Biol Bull 223:44–65. 22983032 10.1086/BBLv223n1p44

[84] Hamm CA, Begun DJ, Vo A, Smith CC, Saelao P, Shaver AO, Jaenike J, Turelli M 2014 Wolbachia do not live by reproductive manipulation alone: infection polymorphism in Drosophila suzukii and D. subpulchrella. Mol Ecol 23:4871–4885. doi:10.1111/mec.12901. 25156506 PMC4180775

[85] Gutzwiller F, Carmo CR, Miller DE, Rice DW, Newton IL, Hawley RS, Teixeira L, Bergman CM 2015 Dynamics of Wolbachia pipientis gene expression across the Drosophila melanogaster life cycle. G3 (Bethesda) 5:2843–2856. doi:10.1534/g3.115.021931. 26497146 PMC4683655

[86] Bordenstein SR, Theis KR 2015 Host biology in light of the microbiome: ten principles of holobionts and hologenomes. PLoS Biol 13:e1002226. doi:10.1371/journal.pbio.1002226. 26284777 PMC4540581

