# Supplementary material for: Wolbachia Endosymbionts Modify Drosophila Ovary Protein Levels in a Context-Dependent Manner
Source: Appl Environ Microbiol. 2016 Aug 15;82(17):5354–63. doi: 10.1128/AEM.01255-16 (PMC4988175; doi:10.1128/AEM.01255-16)
Supplement: Supplemental material [file supp_82_17_5354__index.html]

Supplemental material 

# Wolbachia Endosymbionts Modify Drosophila Ovary Protein Levels in a Context-Dependent Manner

## Supplemental material

- Supplemental file 1 -

  Assessment of ovarian *Wolbachia* titers by real-time qPCR (Fig. S1), initial detection and analysis of ovarian proteins from *D. melanogaster* (Table S1) and *D. simulans* (Table S2), significant proteins identified in *D. melanogaster* (Table S3) and *D. simulans* (Table S4), and comparison of *Wolbachia* impact on the *Drosophila* ovary proteome to results from prior studies (Table S5).

  PDF, 1.0M
