# Supplementary material for: Wolbachia Endosymbionts Modify Drosophila Ovary Protein Levels in a Context-Dependent Manner
Source: Appl Environ Microbiol. 2016 Aug 15;82(17):5354–63. doi: 10.1128/AEM.01255-16 (PMC4988175; doi:10.1128/AEM.01255-16)
Supplement: Supplemental material [file AEM.01255-16_zam999117381so1.pdf]

SUPPLEMENTAL LEGENDS

Figure S1. Assessment of ovarian *Wolbachia* titers by real-time qPCR. Absolute quantification of Wsp copy number per ovary is shown. The Y-axis displays *Wolbachia* titer in each condition relative to the amount carried by a single ovary equivalent from Dmel *w*Mel. 60 ovaries were assessed per host-strain combination. Asterisk indicates significant change in Wsp copy number according to ANOVA ($p < 0.05$).

Table S1. Initial detection and analysis of ovarian proteins from *D. melanogaster.* The columns report on the initial detection, the availability of quality peptides and reliability of detection for each protein. Each row represents a different protein, with all UniProt IDs pertaining to each protein displayed together.

Table S2. Initial detection and analysis of *D. simulans* ovarian proteins. Each row of the table reports on the initial detection of each protein, the availability of quality peptides for that protein and reliability of protein detection between biological and technical replicates. For proteins represented by multiple UniProt IDs, all corresponding ID information is grouped together in the table.

Table S3. Significant proteins detected in *D. melanogaster* ovarian tissues. These proteins are significantly different in abundance in one or more *Wolbachia*-infected samples, according to ANOVA ($p < 0.05$). Average LFQ intensity values report the abundance of each protein. The relative abundance ratios compare these average LFQ scores for each sample type: Dmel wMel/Dmel Uninf; Dmel wMel^{CS}/Dmel Uninf; and Dmel wMel^{CS}/Dmel wMel.

Table S4. Significant proteins identified in *D. simulans* ovarian tissues. These proteins exhibit a significant change in abundance in one or more *Wolbachia*-infected samples, according to ANOVA ($p < 0.05$). Average LFQ intensity values display the abundance of each protein. The Relative abundance ratios compare average LFQ values for each sample type: Dsim wRi/Dsim Cured; Dsim wMel/Dsim Cured; and Dsim wMel/Dsim wRi.

Table S5. Comparing *Wolbachia* impact on the *Drosophila* ovary proteome to prior studies. All data are standardized to display abundance changes in terms of Infected/Uninfected. Red text indicates up-regulation of the gene product. Blue text represents down-regulation. Purple text indicates that the directionality of abundance shifts was inconsistent. Absence of text indicates that either no change was observed or no data was available.

Figure S1

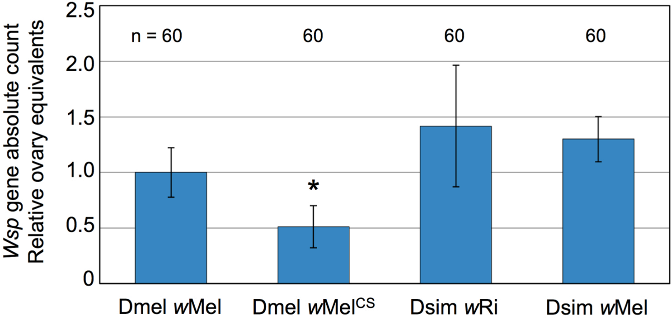

2.5
2.0
1.5
1.0
0.5
0
Wsp gene absolute count
Relative ovary equivalents
n = 60
60
60
60
*
Dmel wMel
Dmel wMelCS
Dsim wRi
Dsim wMel

Table S1

Protein IDs	Protein detection, according to absolute quantification (iBAQ)				Protein quality, according to relative quantification (LFQ)				Protein reliablity, according to assessment criteria			
	Dmel Uninf	Dmel *w*Mel	Dmel *w*MelCS	detected in all?	Dmel Uninf	Dmel *w*Mel	Dmel *w*MelCS	high quality data in all?	Dmel Uninf	Dmel *w*Mel	Dmel *w*MelCS	reliable overall?
A0A077HCQ5;A0A077HCY9;Q1ECB3	+	+	+	yes	+	+	+	yes	+	+	+	yes
A0APA5;Q8MSC4;A0APB1;A0APB0;A0APA8;A0APA7;A0APA6	+	+	+	yes	+	+	+	yes	-	-	-	no
A1Z6X6;Q8MYU8	+	+	+	yes	+	+	+	yes	-	-	-	no
A1Z784;A8DY67;Q7JV23;D0IQ86	+	+	+	yes	-	-	+	no	-	-	-	no
A1Z8Q2;A8DYB0;A2VEG7	+	+	+	yes	+	+	-	no	-	-	-	no
A1Z8U4;C8VV32;Q7KKI0	+	+	+	yes	+	+	+	yes	+	+	+	yes
A1Z909	-	+	-	no	-	+	-	no	-	-	-	no
A1Z992;U3NIM6;Q8T0H4;B5RJS1	-	+	+	no	-	+	-	no	-	-	-	no
A1ZA05;Q9GSP5;Q0E965;G2J5W6;Q95SW3;Q6NP56;C4JC89;C5WLP3	-	+	-	no	-	+	-	no	-	-	-	no
A1ZAB5;Q8MQR3	+	+	+	yes	+	+	+	yes	-	-	-	no
A1ZAX1	+	+	+	yes	+	+	+	yes	+	+	+	yes
A1ZB71;Q7JZM3	+	+	+	yes	-	-	-	no	-	-	-	no
A1ZB77;Q95RC4;A1ZB80;E1JGL5;A1ZB83;A1ZB78;Q7JRI6;Q8IGG3;A1ZB79;A1ZB82	+	+	+	yes	+	+	+	yes	+	+	+	yes
A1ZBE9	+	+	+	yes	+	+	+	yes	-	-	-	no
A1ZBJ2;C9QPI7	+	+	+	yes	+	+	+	yes	-	+	-	no
A4V2S3;O46036;E8NH65	-	+	+	no	-	+	-	no	-	-	-	no
A4V4J0;M9PJN9;E1JJA5;P27619;A4V4I8;X2JE15;E1JJA4;M9PHQ0;F6J732;E8NH48	+	+	+	yes	-	-	-	no	-	-	-	no
A4VA49;A4VA48;A4VA47;A1ZBU8;Q8IGD1;B5RJ56	+	+	+	yes	-	-	-	no	-	-	-	no
A5XCL5;Q9VSW2;E8NH18;E1JI91;Q9VSW1	+	+	-	no	-	-	-	no	-	-	-	no
A8DY82;E2QCN4;A8DY80;A8DY81;C0PUX1;Q8IH39	+	+	+	yes	-	-	-	no	-	-	-	no
A8DYH2	+	+	+	yes	-	-	-	no	-	-	-	no
A8JRB8;A8JRC1;Q8MT18;A8JRC2	+	+	+	yes	+	+	+	yes	-	-	-	no
A8JUV4;Q38HW4	+	+	+	yes	+	+	+	yes	-	-	-	no
A9YGE4	+	+	+	yes	-	-	-	no	-	-	-	no
B3LF78;Q9NFU0;B3G0T0;B3G0S8;B3G0S3	+	+	+	yes	+	+	+	yes	-	+	+	yes
B6UXR5;B6UXN9;P52304	-	-	+	no	-	-	+	no	-	-	-	no
B7YZK6;B7YZK4;B7YZK5	+	+	+	yes	+	+	+	yes	+	+	+	yes
B7YZQ7;O77460;I0DHK6;B9EQY3	+	+	+	yes	+	+	+	yes	+	+	+	yes
B7Z0X1;Q9W457;F6J3U3;A9YJ11;A9YJ07;A9YJ06	+	+	+	yes	+	+	+	yes	+	+	+	yes
C0PDD8;A1ZA83	+	-	+	no	-	-	-	no	-	-	-	no
C0PUW8;Q9V9S0;Q8SZP0	+	+	+	yes	-	-	+	no	-	-	-	no
C6SV30;A4V364;A8JR54;Q8IN47;Q8IN48;Q86NT3;Q9VDI8;Q7KSA0	+	+	+	yes	-	-	-	no	-	-	-	no
C7LAA5;A8E6M1;A1ZBB4;E1JGL8	+	+	+	yes	-	-	+	no	-	-	-	no
C8AXZ8;C8AXZ6;Q7KB18;Q8SZM7;Q7JRC3;C8AXY8;C8AXY5;C8AXY2;C8AXY1	+	+	+	yes	+	+	+	yes	-	-	-	no
C8VV30;P36872	+	+	+	yes	+	+	+	yes	-	-	-	no
C8VV67;A4V3J6;P07909;A4V3J5;E6PBW8;E8NH29	+	+	+	yes	-	-	-	no	-	-	-	no
D0Z768;E1JH02;Q86S05	+	+	+	yes	-	-	+	no	-	-	-	no
D2A6L8	-	-	+	no	-	-	+	no	-	-	-	no
D2NUG1;A1ZAV4	+	+	+	yes	-	-	+	no	-	-	-	no
D3DMY7	-	+	+	no	-	-	+	no	-	-	-	no
D3DMZ9;Q9V429	+	+	+	yes	+	+	+	yes	-	-	-	no
D5SHM9;E3CTS3;Q8IGH3;A1Z6Z3;Q7JR61;F0JAM9;A4UZ69;A1Z6Z4	+	+	+	yes	-	-	-	no	-	-	-	no
D5SHQ7;C8VV92;P40320;A4UZW2	+	+	+	yes	+	+	+	yes	-	-	-	no
D6W4L2;E6PBY9;P06742	+	+	+	yes	+	+	+	yes	-	-	-	no
D7F9H3;D7F9H2;D7F9H1;D7F9H0;D7F9G9;D7F9G8;D7F9G7;D7F9G6;D7F9G5;D7F9G4;D7F9G3;Q9VDM1	-	+	-	no	-	+	-	no	-	-	-	no
E1JGP0;P53501;C8VV69;C6TP14;Q3ZAJ5;P45891;F0JAT3	+	+	+	yes	+	+	+	yes	+	+	-	no
E1JHJ4;M9ND95;M9NEP1;E1JHJ3;E1JHJ5;B8A423;M9NF46;M9NCU7;P05661;Q26433;Q24410;Q24411	+	+	+	yes	+	+	+	yes	+	+	+	yes
E1JHR5;P15007	+	+	+	yes	+	+	+	yes	+	+	+	yes
E1JHT6;Q7KTP4;Q6NP20;Q9VMV9	+	+	+	yes	+	+	-	no	-	-	-	no
E1JIR0;Q9VDL0	+	+	+	yes	-	-	-	no	-	-	-	no
E1JJ68;C7LAE4;P19109;I0B1P2	+	+	+	yes	+	+	+	yes	+	+	+	yes
E1JJD6;Q9W4M7;Q95RH4;Q7K765;Q7K764;A8WHF5;Q8MSV7	+	+	+	yes	-	-	-	no	-	-	-	no
E2QCF1;Q7KN85;Q6AWP8;Q7JNC6	+	+	+	yes	+	+	-	no	-	-	-	no
E6PBW0;P13607;H9ZJM5;B5RIT8;I7J4E4;E1JIR4;C8VUY7;A4IJ54;Q9U458;A8QI34	+	+	+	yes	+	+	+	yes	+	+	+	yes
F0JAN1;Q9I7S8;A8QI10	+	+	+	yes	+	+	-	no	-	-	-	no

F0JAP1;Q8STG9	+	+	+	yes	+	+	+	yes	+	-	-	no
F0JAP3;Q9Y0Y2	+	+	-	no	-	-	-	no	-	-	-	no
F3YDH6;Q9V470	+	+	+	yes	-	-	-	no	-	-	-	no
F8VAB5;P84345;C7DZL0	+	+	+	yes	-	-	-	no	-	-	-	no
G4LU22;Q9VJ59;Q8SXD0;Q5KU10	+	+	+	yes	-	-	-	no	-	-	-	no
G4LU37;Q8I0G5	+	+	+	yes	-	-	+	no	-	-	-	no
H0RNI8;D2NUK5;Q8MMC4;H9XQA9	+	+	+	yes	-	-	+	no	-	-	-	no
H1UUB1;P18489	+	+	+	yes	-	-	+	no	-	-	-	no
H1UUD2;Q7KMQ5;Q7KRU8;B9EQV2;B8A405;Q8I0T0	+	+	+	yes	+	+	+	yes	+	+	+	yes
H5V8F3;Q9VN93;D5AEL2	-	-	+	no	-	-	+	no	-	-	-	no
J7K3P9;A1Z8L8;Q95TS2;D3DMU9	+	+	+	yes	+	+	+	yes	-	-	-	no
K7WSB4	+	+	+	yes	-	-	-	no	-	-	-	no
L0CRF8;L0CRF4;L0CR49;L0CR44;L0CQR2;L0CQQ8;L0CPM0;L0CPL5;L0CPK9;L0CPE3;L0CPD9;Q0E9B6;A1Z8U9;C4JD65	+	+	+	yes	+	+	+	yes	+	+	+	yes
L7X6D0;R4TCQ6;Q9VZP5;Q9NJH7;Q494K9;M9WSU4;M9NFL1;M9ND37	+	+	+	yes	-	-	+	no	-	-	-	no
M9MS06;F0JAG6;M9PGA7;P18091	+	+	+	yes	+	+	-	no	-	-	-	no
M9MS45;Q9VNX8;B7Z098	+	-	-	no	-	-	-	no	-	-	-	no
M9MSM4;Q7KTV5;M9PFZ1;Q9VNX6;Q7Z2C9	+	+	+	yes	+	+	+	yes	-	-	-	no
M9NEV7;Q9VII1;Q8ST39	+	+	+	yes	+	+	+	yes	-	-	-	no
M9NFR5;Q95RE4;O46173	+	+	+	yes	+	+	+	yes	+	+	+	yes
M9NG39;P41073;D5AEL0;C0PTV1	+	+	+	yes	+	+	+	yes	+	+	-	yes
M9NGK3;X2JDK9;M9NDM1;Q8IRV8;M9NET2;M9NGL3;Q8IRV9;Q9W4Y4;M9NE61;X2JCE8;M9NFS1;E1JJC0;M9NDL5;M9NES6;Q9W4Y3;M9NFR6;X2JE09;A0A023GRW4;Q8IRV7;X2JAC7;M9NE56;Q8MPN3;Q9NEF9;Q9NEG0;Q8MSR5;Q9NEF8;Q9NEG1;Q9NFS9;D5AEP5	+	+	+	yes	+	+	+	yes	+	+	+	yes
M9PCE5;Q9VU94;Q9U5D0	+	+	+	yes	-	-	+	no	-	-	-	no
M9PCG1;Q9VM14;Q7KTK9;M9PCA2;M9PCU4;Q1WWF8;B1PH55;B1PH40;B1PH37;B1PH25;B1PH16;B1PH15;B1PGZ9;B1PH17;B1PH28;B1PH23	+	+	+	yes	+	+	+	yes	-	-	-	no
M9PD27;Q9VPH7;D1Z373;M9PFR9	+	+	+	yes	+	+	+	yes	-	-	-	no
M9PD65;Q9VIW3;M9PBD9;C0LA70;C0LA69;C0LA78;C0LA68	+	+	+	yes	+	+	+	yes	+	-	+	yes
M9PDE7;Q1WWG2;C6SUY0;A8DZ00;Q8IP76;A8DYZ9;E1JHH0;M9ND82;Q9VK08	+	+	+	yes	-	-	-	no	-	-	-	no
M9PDL8;A8DZ24;Q9V9N7;M9PBG0;R9PY70;M9PDE6	+	+	+	yes	+	+	-	no	-	-	-	no
M9PDZ9;M9PDS3;M9PBJ0;M9PGY4;Q9I7U4;M9PDZ6;M9PEA0;M9PBI9;M9PEA5;M9PDS8;M9PEA9;M9PGZ0	+	+	+	yes	+	+	+	yes	-	-	-	no
M9PE40;P35122;M9MRD0	+	+	+	yes	+	+	+	yes	-	-	-	no
M9PET0;M9PC43;B5RIM9;P13706;D5AEK2	+	+	-	no	+	+	-	no	-	-	-	no
M9PF14;M9PF20;M9PFF9;M9PFG3	+	+	+	yes	+	+	+	yes	+	+	+	yes
M9PFG7;P46824;M9PF24	+	+	+	yes	-	-	+	no	-	-	-	no
M9PGB6;Q9VP29	+	+	+	yes	-	-	-	no	-	-	-	no
M9PGX2;Q9VYV3	+	+	+	yes	+	+	+	yes	-	-	-	no
M9PHG4;Q00963;M9PF16;X4YME1	-	+	-	no	-	+	-	no	-	-	-	no
M9PHR2;X2JBP9;E1JJP0;M9PJM6;M9NH07;M9PHJ0;P19351;B9EQV5;E6PBY8	+	+	+	yes	+	+	+	yes	-	-	-	no
M9PJQ5;M9NH88;E4NKM1;M9NFC0;E4NKJ4;A4V4Q6;E4NKJ3;P36188	+	+	+	yes	+	+	+	yes	-	-	-	no
M9WKL1;M9PIA6;O97159;E1JI46;Q59E34;B1PGL6;B1PGM0;B1PGN0;B1PGM9;B1PGM7;B1PGM4;B1PGM1;B1PGL5;B1PGK6;B1PGK4	-	-	+	no	-	-	+	no	-	-	-	no
O01666	+	+	+	yes	+	+	+	yes	+	+	+	yes
O02195	+	+	+	yes	+	+	+	yes	-	-	+	no
O02649;C7LA94;Q9VMN5	+	+	+	yes	+	+	+	yes	+	+	+	yes
O16043	+	+	+	yes	+	+	+	yes	+	+	+	yes
O16797;C6SUV6;C7LA69;E0R984	+	+	+	yes	+	+	+	yes	+	+	+	yes
O17445;A8Y560	+	+	+	yes	+	+	+	yes	+	+	+	yes
O18332;Q8MRA4;O15971;O18338	+	+	+	yes	+	+	+	yes	+	+	+	yes
O18335;B8A3X3;Q8IGH2	+	+	+	yes	+	+	+	yes	-	-	-	no
O18373	+	+	+	yes	+	+	+	yes	-	-	-	no
O18388;M9NDC3	+	+	+	yes	+	+	+	yes	-	+	+	yes
O18404;D5A7S2	+	+	+	yes	+	+	+	yes	+	+	+	yes
O18413;Q9VA54	+	+	+	yes	+	+	+	yes	+	+	+	yes

O18640;M9PCC1;M9PB67;B4YX01;B4YX00;B4YWZ6;F6J206;F6J1Z8;A9YGT1;A9YGT0;A9YGS8;A9YGS7;F6J9X0;F6J9W7;F6J9W5;Q9VYQ9;REV__M9PBG8;REV__Q9W0S2	+	+	+	yes	+	+	+	yes	+	+	+	yes
O44081;E1JGV6;H5V894;C0P8M5	+	+	+	yes	+	+	+	yes	+	+	+	yes
O44437	+	+	+	yes	+	+	+	yes	-	-	-	no
O46067	+	+	+	yes	+	+	+	yes	-	-	-	no
O52076;Q09TN6;Q7BVI0;O52077;B0ZSE1;B7TX60;B7TX59;U6SUJ5;Q73G97;A0EZP4;Q5EFK7;Q5EFK9;Q5EFK8	+	+	+	yes	+	+	+	yes	-	+	+	yes
O61231;M9PIM0	+	+	+	yes	+	+	+	yes	+	+	-	yes
O61345	-	+	-	no	-	+	-	no	-	-	-	no
O61380;A8DZ29;Q7YU50	+	+	+	yes	+	+	+	yes	+	+	+	yes
O61613	+	+	+	yes	+	+	+	yes	-	-	-	no
O61650;Q9VPN5;Q867V1;Q867Y1	+	+	+	yes	+	+	+	yes	+	+	+	yes
O62619	+	+	+	yes	+	+	+	yes	+	+	+	yes
O62621	+	+	+	yes	-	-	+	no	-	-	-	no
O76454	+	-	-	no	-	-	-	no	-	-	-	no
O76742	+	+	+	yes	-	-	+	no	-	-	-	no
O76894;M9MS86	+	+	+	yes	-	-	+	no	-	-	-	no
O76922;Q95PE5	+	+	+	yes	+	+	-	no	-	-	-	no
O76927;M9PEA6;E4NKJ9;A2VEP1	+	+	+	yes	+	+	+	yes	-	-	-	no
O77410;Q8MR88	+	+	+	yes	+	+	-	no	-	-	-	no
O96827	+	+	+	yes	+	+	+	yes	+	+	+	yes
O97066;Q3YMT4	+	+	+	yes	+	+	+	yes	-	-	-	no
O97102	+	+	+	yes	+	+	+	yes	-	+	-	no
O97118;A1A6X4;Q9W0A8;Q0ZHI2;Q8SX05	+	+	+	yes	+	+	+	yes	+	+	+	yes
O97125;Q9VG58;Q9BIS2;Q9BIR7;Q8INI8;F3YDH1;C7LA76;P82910;P02825;F3YDI6	+	+	+	yes	+	+	+	yes	+	+	+	yes
O97418;Q8SYB3	+	+	+	yes	+	+	-	no	-	-	-	no
O97428;D5AEL7;Q8IRS7	+	+	+	yes	+	+	+	yes	+	+	+	yes
O97477	+	+	+	yes	-	-	+	no	-	-	-	no
P00334	+	+	+	yes	-	-	-	no	-	-	-	no
P00408;J9TIE4;J9THZ9;C6KI54;C8CB16	+	+	+	yes	+	+	+	yes	+	-	-	no
P02283	+	+	+	yes	+	+	+	yes	+	+	+	yes
P02828;M9PBL3	+	+	+	yes	+	+	+	yes	+	+	+	yes
P04359;G8E4R5	+	+	+	yes	+	+	+	yes	+	+	+	yes
P05205	+	+	+	yes	-	-	+	no	-	-	-	no
P05389;B7FNN6	+	+	+	yes	+	+	+	yes	+	+	+	yes
P06605;P06603;A4V2J2;K7XI17;K7X561;K7WSB1;K7WQ63;K7WQ56;K7WKV5;K7WKV0;K7XI11;P06604	+	+	+	yes	+	+	+	yes	+	+	+	yes
P06606;Q6NP92	+	+	+	yes	+	+	+	yes	+	+	+	yes
P06754;Q8IGY1	+	+	+	yes	+	+	+	yes	+	+	+	yes
P07182;D1Z363	+	+	+	yes	+	+	+	yes	-	+	+	no
P07183;E1JJF7;B3DNA9;B3DNM9;B3DNC0	+	+	+	yes	+	+	+	yes	-	+	+	no
P07184;C0PVD4	+	+	+	yes	-	-	-	no	-	-	-	no
P07185	+	+	+	yes	+	+	+	yes	-	-	+	yes
P07186	+	+	+	yes	+	+	+	yes	-	-	+	no
P07486	+	+	+	yes	+	+	+	yes	-	-	-	no
P07487;M9PJN8	+	+	+	yes	+	+	+	yes	+	+	+	yes
P07764;C8VV14;F3YDE2;F3YDB5;A4V3G1;F3YDA0;Q86NZ4	+	+	+	yes	+	+	+	yes	+	+	+	yes
P08120;B7FNQ3	+	+	+	yes	+	+	+	yes	-	-	-	no
P08181;A4V2B8	+	+	+	yes	+	+	+	yes	-	-	-	no
P08570;M9PBK5	+	+	+	yes	+	+	+	yes	+	+	+	yes
P08645;M9MRT6	+	+	+	yes	-	-	+	no	-	-	-	no
P08736;C6TP87;Q8T3U3;P05303;A4V3Q6	+	+	+	yes	+	+	+	yes	+	+	+	yes
P08879;A8E6J4;Q8IH27	+	+	+	yes	+	+	+	yes	+	+	+	yes
P08928;M9NE89	+	+	+	yes	+	+	+	yes	+	+	+	yes
P08985	+	+	+	yes	+	+	+	yes	+	+	+	yes
P09052;M9PBB5	+	+	+	yes	+	+	+	yes	-	+	-	no
P09180;Q1EBX5	+	+	+	yes	+	+	+	yes	+	+	+	yes
P09491;H1UUJ7	+	+	+	yes	+	+	+	yes	+	+	+	yes
P10981;P83967;B6IDW7;P02574;M9PFZ6	+	+	+	yes	-	-	-	no	-	-	-	no
P11046	+	+	+	yes	+	+	-	no	-	-	-	no
P11147;C7LA75;Q8I0E9	+	+	+	yes	+	+	+	yes	+	+	+	yes
P12613;A4V391	+	+	+	yes	+	+	+	yes	+	+	+	yes
P12881;I0DHK3	+	+	+	yes	+	+	+	yes	+	+	+	yes
P12982;Q05547;C0MKX3;B4F4X2;Q95V52;Q95V51;Q5K375;Q5LJN2;A8QI94;P48461	+	+	+	yes	+	+	+	yes	-	-	-	no
P13008;M9PG47	+	+	+	yes	+	+	+	yes	+	+	-	yes
P13060	+	+	+	yes	+	+	+	yes	+	+	+	yes
P13395;M9PDQ0;M9PBI5;M9PGV6	+	+	+	yes	+	+	+	yes	-	-	-	no
P13469;Q8IGX8	+	+	+	yes	+	+	+	yes	+	+	+	yes
P17210;Q6NNT8	+	+	+	yes	+	+	+	yes	-	-	-	no
P17336	+	+	+	yes	-	-	+	no	-	-	-	no
P17704	+	+	+	yes	+	+	+	yes	+	+	+	yes
P17917;Q868I9	+	+	+	yes	+	+	+	yes	-	+	-	no
P18053;Q9VA12	+	+	+	yes	+	+	+	yes	+	+	+	yes

P18169;M9NEA7;P18171;D3PFE0;P18170;M9NEY9;M9NG31	+	+	+	yes	+	+	+	yes	+	+	+	yes
P19889;M9PG76	+	+	+	yes	+	+	+	yes	+	+	+	yes
P20240	+	+	+	yes	+	+	+	yes	-	-	-	no
P20432	+	+	+	yes	+	+	+	yes	+	+	+	yes
P20477;E1JHQ1	+	+	+	yes	+	+	+	yes	-	-	-	no
P21187	+	+	+	yes	+	+	+	yes	+	+	+	yes
P21914;Q9VWN3;Q8SXL9	+	+	+	yes	+	+	-	no	-	-	-	no
P22058;A4V2K7	+	+	+	yes	+	+	+	yes	-	-	-	no
P22464	+	+	+	yes	+	+	+	yes	-	-	-	no
P22700;E8NHA8	+	+	+	yes	+	+	+	yes	+	+	+	yes
P22769	+	+	+	yes	+	+	+	yes	+	+	-	yes
P22977;B3DNC3	+	+	+	yes	+	+	+	yes	-	-	-	no
P23128	+	+	+	yes	+	+	+	yes	+	+	+	yes
P23572;C0MJ66	-	+	-	no	-	+	-	no	-	-	-	no
P23779	+	+	+	yes	-	-	-	no	-	-	-	no
P24156;A0A023GQA5;V9H1B9	+	+	+	yes	+	+	+	yes	+	+	+	yes
P25007;A8E774;F6J7B3;Q7K2I4;Q5U0U2	+	+	+	yes	+	+	+	yes	+	+	+	yes
P25161;M9PG62;B1Q010	+	+	+	yes	+	+	+	yes	+	+	+	yes
P25439;M9PFM5;M9PFS6	+	+	+	yes	-	-	-	no	-	-	-	no
P25823	+	+	+	yes	-	-	+	no	-	-	-	no
P25843;M9NEU5	+	+	+	yes	+	+	+	yes	-	-	-	no
P25867	+	+	+	yes	-	-	-	no	-	-	-	no
P26270	+	+	+	yes	+	+	+	yes	-	-	-	no
P26686	+	+	+	yes	+	+	+	yes	+	+	+	yes
P28668;H8F4R5	+	+	+	yes	+	+	+	yes	+	+	+	yes
P28750;E1JGN9	+	+	+	yes	+	+	+	yes	-	-	+	yes
P29310;Q6LEH5	+	+	+	yes	+	+	+	yes	+	+	+	yes
P29327;H1ZYF1;D2NUH9;A8E700;Q95TP9	+	+	+	yes	+	+	+	yes	+	+	+	yes
P29613;K7Z7Z8	+	+	+	yes	+	+	+	yes	+	+	+	yes
P29843;F3YDH5;Q8IH88;Q7JQR9	+	+	+	yes	+	+	-	no	-	-	-	no
P29844;F3YDH0;F6J5W4;Q95NM1	+	+	+	yes	+	+	+	yes	+	+	+	yes
P29845	+	+	+	yes	+	+	+	yes	+	+	+	yes
P31009;M9PB84	+	+	+	yes	+	+	+	yes	+	+	+	yes
P31409;E1JIJ5	+	+	+	yes	+	+	+	yes	+	+	+	yes
P32234	+	+	+	yes	+	+	+	yes	-	-	-	no
P32392	+	+	+	yes	+	+	-	no	-	-	-	no
P33438;M9NCS8	+	+	+	yes	+	+	+	yes	-	-	-	no
P35128;E1JJN1;Q9VJS5	+	+	+	yes	+	+	+	yes	-	-	-	no
P35381;U6SW43;Q73HB2	+	+	+	yes	+	+	+	yes	+	+	+	yes
P35415;A4V1N8;P35416;A4V1N9	+	+	+	yes	+	+	-	no	-	-	-	no
P36179;M9MQH9;M9NDI6;H5V8B8;Q8MT47	+	+	+	yes	+	+	+	yes	+	+	+	yes
P36241	+	+	+	yes	+	+	+	yes	+	+	+	yes
P37276;M9PE73;M9PEC8;M9PBQ0;M9PEN4;M9PHG8;M9PBQ3;Q1EBX0	+	+	+	yes	+	+	+	yes	-	-	-	no
P39018;E2QD65	+	+	+	yes	+	+	+	yes	+	+	+	yes
P40301	+	+	+	yes	+	+	+	yes	-	+	+	yes
P40304;O77288	+	+	+	yes	+	+	+	yes	-	-	+	no
P40423;F6J1D0	+	+	+	yes	+	+	+	yes	-	-	-	no
P41043;A4UZL5	+	+	+	yes	-	-	-	no	-	-	-	no
P41092;M9MRC9	+	+	+	yes	+	+	+	yes	+	+	+	yes
P41093;Q4V4E7	+	+	+	yes	+	+	+	yes	+	+	+	yes
P41094;B6IDT0;Q4V607	+	+	+	yes	+	+	+	yes	+	+	+	yes
P41126;M9PFF0	+	+	+	yes	+	+	+	yes	+	+	+	yes
P41375;F3YDB2	+	+	+	yes	+	+	+	yes	-	+	+	yes
P41572;M9PIS3;K7ZI00	+	+	+	yes	-	-	+	no	-	-	-	no
P42271;M9PDN9	-	+	-	no	-	+	-	no	-	-	-	no
P45437	+	+	+	yes	+	+	+	yes	-	-	-	no
P45594	+	+	+	yes	+	+	+	yes	+	+	+	yes
P45889	+	+	+	yes	+	+	-	no	-	-	-	no
P46150;M9NG50;M9PHG2;C7LAH9;C9QP54	+	+	+	yes	+	+	+	yes	+	+	+	yes
P46222;Q4V5R3	+	+	+	yes	+	+	+	yes	+	+	+	yes
P46415	+	+	+	yes	+	+	+	yes	-	-	+	no
P48149;E1JJM9;Q7KR04	+	+	+	yes	+	+	+	yes	+	+	+	yes
P48159;A8E792	+	+	+	yes	+	+	+	yes	+	+	+	yes
P48375;H0RN81	+	+	+	yes	+	+	+	yes	+	+	-	no
P48588	+	+	+	yes	+	+	+	yes	+	+	+	yes
P48591	+	+	+	yes	-	-	+	no	-	-	+	no
P48592	-	-	+	no	-	-	+	no	-	-	-	no
P48598;M9PBZ9;Q683T0	+	+	+	yes	+	+	+	yes	+	+	+	yes
P48601	+	+	+	yes	+	+	+	yes	-	+	+	yes
P48605;A4V303	+	+	+	yes	+	+	+	yes	+	+	+	yes
P48610;A8JNP1;A8JNP2	+	+	+	yes	+	+	+	yes	+	+	+	yes
P48809;L0CPX5;E1JHA4;L0CQ56;L0CRN5;L0CRY8;L0CRY2;L0CQ61;L0CRN0;L0CR94	+	+	+	yes	+	+	+	yes	+	+	+	yes
P48810;E1JIK0	+	+	+	yes	+	+	+	yes	+	-	-	no
P49735	+	+	+	yes	-	-	+	no	-	-	-	no
P50887	+	+	+	yes	+	+	+	yes	+	+	+	yes
P52029	+	+	+	yes	+	+	+	yes	-	-	-	no
P52034;H5V888;C8VV04;C7LA80	+	-	+	no	-	-	+	no	-	-	-	no
P52295	+	+	+	yes	+	+	+	yes	+	+	+	yes
P53997	+	+	+	yes	-	-	-	no	-	-	-	no
P54350;M9PB57	+	+	+	yes	-	-	-	no	-	-	-	no
P54357;M9NEW1	+	+	+	yes	+	+	+	yes	+	+	+	yes
P54385;Q8IMY1	+	+	+	yes	+	+	+	yes	+	+	+	yes

P54397	+	+	+	yes	+	+	+	yes	+	+	+	yes
P54611	+	+	+	yes	+	+	+	yes	+	+	+	yes
P54622	+	+	+	yes	+	+	+	yes	-	-	+	no
P55035;M9PIG8	+	+	+	yes	+	+	+	yes	-	-	+	no
P55828	+	+	+	yes	+	+	+	yes	+	+	+	yes
P55830	+	+	+	yes	+	+	+	yes	+	+	+	yes
P55935;C6SUW3;Q95RG1	+	+	+	yes	+	+	+	yes	+	+	+	yes
P56538	+	+	+	yes	-	-	-	no	-	-	-	no
P61209;M9PG22	+	+	+	yes	+	+	+	yes	-	+	-	no
P61851;M9PF91	+	+	+	yes	+	+	+	yes	+	+	+	yes
P61857;Q7M3K0;Q7M3K1;Q7M3J9	+	-	+	no	-	-	+	no	-	-	-	no
P62152;C6SUZ2	+	+	+	yes	+	+	+	yes	+	+	+	yes
P80455	+	+	+	yes	+	+	+	yes	-	-	-	no
P84040	+	+	+	yes	+	+	+	yes	+	+	+	yes
P84249;P02299;E2QCP0;Q6TXQ1	+	+	+	yes	+	+	+	yes	+	+	+	yes
P91926;E8NH12	+	+	-	no	+	+	-	no	-	-	-	no
P91928	+	+	+	yes	+	+	+	yes	-	-	-	no
P91929;A4V383	+	+	+	yes	+	+	+	yes	-	-	-	no
P91938;Q9VNT5	+	+	+	yes	+	+	+	yes	+	+	+	yes
P92177;C6TP70;D3DMY5	+	+	+	yes	+	+	+	yes	+	+	+	yes
P98159	+	+	+	yes	-	-	+	no	-	-	-	no
P98163	+	+	+	yes	+	+	+	yes	+	+	+	yes
Q00174;B3DN78	+	+	+	yes	+	+	+	yes	+	+	-	yes
Q00637	+	+	+	yes	-	-	-	no	-	-	-	no
Q01604;M9PCE0;U6SZR0;P62422	+	+	+	yes	+	+	+	yes	+	+	+	yes
Q01637	+	+	+	yes	-	-	+	no	-	-	-	no
Q02427;Q9VYD8;D3DMZ8;M9MS48	+	+	+	yes	-	-	+	no	-	-	-	no
Q02748;D3DMH8;C9QP42;C8VV33	+	+	+	yes	+	+	+	yes	+	+	+	yes
Q03427	+	+	+	yes	+	+	-	no	-	-	-	no
Q05783;C0MJA0	+	+	+	yes	+	+	+	yes	-	-	-	no
Q05856	+	+	+	yes	-	-	-	no	-	-	-	no
Q06521	+	+	+	yes	-	-	-	no	-	-	-	no
Q06559;G3M3A2;Q29QY2	+	+	+	yes	+	+	+	yes	+	+	+	yes
Q06943	+	+	+	yes	-	-	-	no	-	-	-	no
Q07152;A4V488;A9YKX5;F6J5F7	+	+	+	yes	+	+	+	yes	-	-	-	no
Q08473	+	+	+	yes	+	+	+	yes	+	+	+	yes
Q09332	-	+	-	no	-	+	-	no	-	-	-	no
Q0E8P6	+	+	+	yes	+	+	-	no	-	-	-	no
Q0E8V7	+	+	+	yes	-	-	-	no	-	-	-	no
Q0E924;A8DYI7;A8DYI6	+	+	+	yes	+	+	+	yes	+	+	+	yes
Q0E940;E2QCG7	+	+	+	yes	+	+	+	yes	+	+	+	yes
Q0KHZ6;Q8MR58	+	+	+	yes	+	+	+	yes	+	+	+	yes
Q1EC13;Q9VWI0	+	+	+	yes	-	-	-	no	-	-	-	no
Q23997	+	+	+	yes	-	-	+	no	-	-	-	no
Q24009;E2QCR4	+	+	+	yes	+	+	+	yes	-	-	-	no
Q24050	+	+	+	yes	-	-	+	no	-	-	-	no
Q24208;Q53XD3;P45975;I0DHL3	+	+	+	yes	+	+	+	yes	+	+	+	yes
Q24251	+	+	+	yes	+	+	+	yes	+	+	+	yes
Q24253;Q960F2	+	+	+	yes	+	+	+	yes	-	-	-	no
Q24319	+	+	+	yes	+	+	+	yes	-	-	-	no
Q24407;D1Z3A2	+	+	+	yes	+	+	+	yes	+	+	+	yes
Q24439;Q8INE6	+	+	+	yes	+	+	+	yes	+	+	+	yes
Q24492	+	+	+	yes	+	+	+	yes	-	+	+	yes
Q24507;Q24506;Q9VJ43;Q9VJ44	+	+	+	yes	-	-	+	no	-	-	-	no
Q24572;C0MJE4;E1JIL4	+	+	+	yes	+	+	+	yes	-	-	-	no
Q24576;Q24577;Q7JYK1;P18101;Q8IGR0;P15357;Q8MT02;Q9W418;P0CG69;A4V1F9;Q8MSM5;Q7JPZ3;Q7JPZ2;Q27927;H9BVD7;H9BVD5;H9BVD4;H9BVD3;H9BVD2;R9PY16;Q24575	+	+	+	yes	+	+	+	yes	+	+	+	yes
Q26454;Q5BI95	+	+	+	yes	-	-	-	no	-	-	-	no
Q27268;M9PC90;H1UUC9	+	+	+	yes	+	+	+	yes	+	+	+	yes
Q27331;A4V0N4;Q8T464;Q9VK47	+	+	+	yes	+	+	+	yes	+	+	+	yes
Q27415	+	+	+	yes	+	+	+	yes	+	+	+	yes
Q27889;M9NE01;Q9VXF1;M9NEL1;P48456	+	+	+	yes	+	+	-	no	-	-	-	no
Q2XYH0;Q9VSR5	+	+	+	yes	+	+	+	yes	+	-	-	no
Q3YMU0;Q9TWZ1;Q95T75	+	+	+	yes	+	+	+	yes	+	+	+	yes
Q4ABD8;Q4AB54;P02255;Q4ABE3;Q4AB94;Q5BI49;C4IY08	+	+	+	yes	+	+	+	yes	+	+	-	yes
Q4QPQ7;Q9V3W2	+	+	+	yes	+	+	-	no	-	-	-	no
Q4QQC4;Q0KIB3	+	+	+	yes	-	-	+	no	-	-	-	no
Q4V3L7;Q4V3F7;Q9Y112;Q4V403	+	+	+	yes	+	+	+	yes	-	-	-	no
Q500Y7	+	+	+	yes	-	-	-	no	-	-	-	no
Q541C9;O76511	+	+	+	yes	-	-	-	no	-	-	-	no
Q58SN2;Q58SN1;Q58SM9;Q58SN4;Q58SN3;Q58SM8;Q9VB46;Q8MT56;Q24160;D2NUG9;Q58SN0	+	+	+	yes	+	+	-	no	-	-	-	no
Q59E58;Q59E59;J7JVR0;C9QPB4;D0IQ99;C9QPE3;Q1WWE4	+	+	+	yes	+	+	+	yes	+	+	+	yes
Q5BI73	+	+	+	yes	-	-	+	no	-	-	-	no
Q5U0W4	+	+	+	yes	+	+	+	yes	+	+	+	yes
Q6IDF5	+	+	+	yes	+	+	-	no	-	-	-	no
Q6IGW6	+	+	+	yes	-	-	+	no	-	-	-	no

Q6NN28	+	+	+	yes	+	+	+	yes	-	-	-	no
Q6NP15;Q7KV89;Q7M3J7;Q7KV88;Q7KV90;M9PBJ7;M9PH19;Q9W002	+	+	+	yes	-	-	+	no	-	-	-	no
Q6NP22;Q3ZAN3;A1Z843;B9EQR0;Q95SU3	+	+	+	yes	+	+	+	yes	-	-	-	no
Q6NP72	+	+	+	yes	-	-	+	no	-	-	-	no
Q6NR40;P15348;M9PDQ1	+	+	+	yes	+	+	+	yes	-	-	-	no
Q76NQ0;R9S0M5;Q8IH64;Q6NP35	+	+	+	yes	+	+	+	yes	+	+	+	yes
Q7JQH9;Q9V3K9	-	+	+	no	-	-	+	no	-	-	-	no
Q7JR58	+	+	+	yes	+	+	+	yes	+	+	+	yes
Q7JR71;Q95T42;D8FT33;Q0E9C3;Q5U195	+	+	+	yes	-	-	-	no	-	-	-	no
Q7JUF8;M9PG98;O46091	+	+	+	yes	+	+	-	no	-	-	-	no
Q7JVI3	+	+	+	yes	+	+	+	yes	+	+	+	yes
Q7JVY0	+	+	+	yes	-	-	+	no	-	-	-	no
Q7JWF1	+	+	+	yes	-	-	-	no	-	-	-	no
Q7JWR9	+	+	+	yes	+	+	+	yes	-	-	-	no
Q7JXC4;A0APE4;A0APE6	+	+	+	yes	+	+	+	yes	+	+	+	yes
Q7JXZ2;O97121	+	+	+	yes	+	+	+	yes	+	+	+	yes
Q7JYN2;Q9VX36	+	+	+	yes	-	-	+	no	-	-	-	no
Q7JZN0	+	+	+	yes	+	+	+	yes	-	-	-	no
Q7JZW2;A1ZAH8	+	+	+	yes	+	+	+	yes	+	+	+	yes
Q7K0E6;Q9XYM1	+	+	+	yes	+	+	+	yes	-	-	-	no
Q7K110	+	+	+	yes	+	+	+	yes	-	-	-	no
Q7K2G1	+	+	+	yes	+	+	+	yes	-	-	+	no
Q7K2L7	+	+	+	yes	-	-	-	no	-	-	-	no
Q7K2N0	+	+	+	yes	+	+	-	no	-	-	-	no
Q7K2P3;A1Z7Z4;Q8IGD2;C9QP80	+	+	+	yes	+	+	-	no	-	-	-	no
Q7K3B7	+	+	+	yes	+	+	+	yes	-	-	-	no
Q7K3D4	+	+	+	yes	+	+	-	no	-	-	-	no
Q7K3J0	+	+	+	yes	+	+	+	yes	+	+	+	yes
Q7K3Z3	+	+	+	yes	-	-	+	no	-	-	-	no
Q7K485	+	+	+	yes	+	+	+	yes	-	-	-	no
Q7K4H4;E2QD64	+	+	+	yes	+	+	+	yes	-	-	-	no
Q7K4L8	+	+	-	no	-	-	-	no	-	-	-	no
Q7K550	+	+	+	yes	+	+	+	yes	-	-	-	no
Q7K581	+	+	+	yes	-	-	+	no	-	-	-	no
Q7K5K3;Q8IGJ4;Q8IML6	+	+	+	yes	+	+	+	yes	-	-	-	no
Q7K7G0;Q9W555;Q9U8A9	+	+	+	yes	+	+	-	no	-	-	-	no
Q7KAK2;Q7KN61	-	-	+	no	-	-	+	no	-	-	-	no
Q7KJ37;Q9VEX6;Q95TQ1	+	+	+	yes	-	-	-	no	-	-	-	no
Q7KLV9	+	+	+	yes	+	+	+	yes	+	+	+	yes
Q7KLW9	+	+	-	no	+	+	-	no	-	-	-	no
Q7KLX3	+	+	+	yes	+	+	+	yes	-	-	+	yes
Q7KM15;Q9U9Q7	+	+	+	yes	+	+	+	yes	+	+	+	yes
Q7KMM4	+	+	+	yes	+	+	-	no	-	-	-	no
Q7KMP8;Q29QY1	+	+	+	yes	+	+	+	yes	+	+	+	yes
Q7KMQ0;Q9VQN8;M9PC18	+	+	+	yes	+	+	+	yes	+	+	+	yes
Q7KN62;D0IQG4;Q8IGZ2	+	+	+	yes	+	+	+	yes	+	+	+	yes
Q7KN75;Q9U982;Q7KN84;Q95T04	+	+	+	yes	+	+	+	yes	+	+	+	yes
Q7KN94;Q7KLW5	+	+	+	yes	+	+	+	yes	+	+	+	yes
Q7KN97;Q0E9E2;Q86NV1;Q6NKL9	+	+	+	yes	-	-	+	no	-	-	-	no
Q7KNF3;A1Z6L9;Q8IHC2;Q8MSU6	+	+	+	yes	-	-	-	no	-	-	-	no
Q7KSQ0	+	+	+	yes	+	+	+	yes	-	-	-	no
Q7KSU6;Q9VHN7;Q95S19;Q9VVP4;Q8MZ62	+	+	+	yes	+	+	+	yes	+	+	+	yes
Q7KT36;B3DNB7;B3DNA8	+	+	+	yes	+	+	+	yes	-	-	+	no
Q7KUB0;Q8IQA7;Q9VSI6;Q7KUB1;B7Z0E0;C8VV61	+	+	+	yes	+	+	+	yes	+	+	+	yes
Q7KVQ0;A8E6K0	+	+	+	yes	+	+	+	yes	-	-	-	no
Q7KVU4;O96676;M9PHB8	+	+	+	yes	-	-	+	no	-	-	-	no
Q7KVX5;Q9W4N8;A9YI35	+	+	+	yes	+	+	+	yes	+	-	-	no
Q7KW39;I0B1N8;A9UN30	+	+	+	yes	+	+	+	yes	-	+	-	no
Q7KYI0;Q7JWD6	+	+	-	no	-	-	-	no	-	-	-	no
Q7PLL1;Q8SYL3;L7EEF1;Q7PLL3	+	+	+	yes	+	+	+	yes	-	-	-	no
Q7YWB4;Q7K221;A1ZAA5;Q8IHB4	+	+	+	yes	+	+	+	yes	-	-	-	no
Q868Z9;F0JAG3;D0IQI7	+	+	+	yes	+	+	+	yes	-	+	-	no
Q86BL8;Q8MRT9;Q86BL9	+	+	+	yes	-	-	-	no	-	-	-	no
Q86DS1;E2QD63;C4NYP8;Q8MSG4	+	+	+	yes	+	+	+	yes	+	+	+	yes
Q86NM8;Q24276;C4IY07	+	+	+	yes	-	-	+	no	-	-	-	no
Q86NN5;A0A024E3A5;Q9VPE2;Q059D4	+	+	+	yes	-	-	+	no	-	-	-	no
Q86NQ0;Q9VN44;Q960E2	+	+	+	yes	+	+	+	yes	+	+	+	yes
Q86NR8;Q0E996;Q5KTT4	+	+	+	yes	+	+	-	no	-	-	-	no
Q86NS4;A1Z7H3;Q8T3L1;A1Z7H2;Q960I0;H1UUA9	+	+	+	yes	-	-	+	no	-	-	-	no
Q86NS6;A1Z9E3;Q95TV3;U6SZG4;U6SW27;Q73IX6;Q73H85	+	+	+	yes	+	+	+	yes	+	+	+	yes
Q8I937;Q5U108;Q8MR24	+	+	+	yes	+	+	-	no	-	-	-	no
Q8IA36;Q9W253	+	+	+	yes	+	+	+	yes	-	-	-	no
Q8IA62;Q9VJ11	+	+	+	yes	-	-	+	no	-	-	-	no
Q8IGE9;Q8IPM3;Q9VN21	+	+	+	yes	+	+	+	yes	+	+	+	yes
Q8IMB7;Q9XZE0;Q9V3W9	+	+	-	no	+	+	-	no	-	-	-	no
Q8IMF5;P23226	+	+	+	yes	+	+	+	yes	-	-	-	no

Q8IMG1;Q8IGY5;Q9V9U3;Q7KLU9;Q9U4R5;Q9U4R4;Q9U4R3;Q9U4R0;Q9U4Q8;Q9U4Q6;Q9TW87;Q9TVG2;Q7KLV0;O16040;Q8I0J1;Q86PC7;Q9U4Q7;Q9U4R2;Q9U4Q9	+	+	+	yes	+	+	+	yes	+	-	-	no
Q8IMH4;Q9Y125;Q9U9P3	+	+	+	yes	+	+	+	yes	-	-	-	no
Q8IMI8;Q6NNE3;P18432	+	+	+	yes	+	+	+	yes	-	-	-	no
Q8IN02	-	-	+	no	-	-	+	no	-	-	-	no
Q8INC0;Q5BID0	+	+	-	no	-	-	-	no	-	-	-	no
Q8INH5;D3PFE8;Q8INH6;C7LAE2;Q8IGR1;Q9VFW7;Q86P55;B5RJT1;Q95S62;Q9VFW8;Q8IHC5;Q9VFW9;A9UNG9	+	+	+	yes	-	-	+	no	-	-	-	no
Q8INM3;Q9VGZ8	+	+	+	yes	-	-	+	no	-	-	-	no
Q8IP94;Q8IGC5;Q9VKB0;Q8MRL7	+	+	+	yes	+	+	+	yes	+	+	+	yes
Q8IPB1;Q9V3I1	+	+	+	yes	+	+	+	yes	+	+	+	yes
Q8IPE8;Q9V397	+	+	+	yes	+	+	+	yes	+	+	+	yes
Q8IPG9;M9PCW5;Q961I4;Q7KTJ7	+	+	+	yes	+	+	-	no	-	-	-	no
Q8IPT1;A8E6M8;Q8IPT0;Q6AWP4;Q29QP3	+	+	+	yes	-	-	-	no	-	-	-	no
Q8IPX7	-	+	+	no	-	-	+	no	-	-	-	no
Q8IQP2;Q9VV89;D2NUJ2	+	+	+	yes	-	-	+	no	-	-	-	no
Q8IQW2;Q9VWD1;C1C592	+	+	+	yes	-	-	-	no	-	-	-	no
Q8IR24;Q9VXN2	+	+	+	yes	-	-	-	no	-	-	-	no
Q8IR93;Q9VZ20	-	+	+	no	-	+	-	no	-	-	-	no
Q8IRD0	+	+	+	yes	-	-	-	no	-	-	-	no
Q8IRG6	+	+	+	yes	-	-	-	no	-	-	-	no
Q8IRQ5;Q9W3X6;Q8SXM1;Q9VTI5;Q9W3X5	+	+	+	yes	+	+	+	yes	-	+	-	no
Q8MKK1;Q8MTU4	-	+	+	no	-	-	+	no	-	-	-	no
Q8MLS0;Q0E8X8;Q8SYU1	+	+	+	yes	+	+	+	yes	-	-	-	no
Q8MLY8	+	+	+	yes	+	+	+	yes	+	+	+	yes
Q8MQI1;M9PE74;Q9W2U7	+	+	+	yes	-	-	-	no	-	-	-	no
Q8MRW1	+	+	+	yes	-	-	-	no	-	-	-	no
Q8MSI7;Q9VQD7	-	+	+	no	-	-	+	no	-	-	-	no
Q8MSW0	+	+	+	yes	+	+	+	yes	+	+	+	yes
Q8MT06	+	+	+	yes	+	+	-	no	-	-	-	no
Q8MT58;E8NHA0	+	+	+	yes	+	+	+	yes	-	-	-	no
Q8MYR7;Q9W4X4;M9PDM4;Q9U1K8	+	+	+	yes	-	-	-	no	-	-	-	no
Q8MZI3;Q7KU78;Q8T0I3	+	+	+	yes	+	+	-	no	-	-	-	no
Q8SWR8	+	+	+	yes	+	+	-	no	-	-	-	no
Q8SWU7;E6PBV6;C9QP30	+	+	+	yes	+	+	+	yes	-	-	-	no
Q8SX57	+	+	+	yes	-	-	-	no	-	-	-	no
Q8SXM8;Q9W327;G7H7Z0	+	+	+	yes	+	+	+	yes	-	+	-	no
Q8SXT0;Q9VMI3;Q5U0T0;A1ZAW7;C6SUV8	+	+	+	yes	-	-	+	no	-	-	-	no
Q8SXW1;Q24154;B7FNL1	+	+	+	yes	+	+	+	yes	-	-	-	no
Q8SY19;O97471;D3DMY1;D2NUI8;E4NKK2;Q8SY70	+	+	+	yes	+	+	+	yes	-	-	-	no
Q8SY77;M9NEX3;Q8IRR0;D2NUJ7	+	+	+	yes	-	-	+	no	-	-	-	no
Q8SYJ2	+	+	+	yes	+	+	+	yes	-	-	-	no
Q8SYN6;Q9VSC5	+	+	+	yes	-	-	-	no	-	-	-	no
Q8T060;Q6NKM4;Q9VUR3;E4NKL6	+	+	+	yes	+	+	-	no	-	-	-	no
Q8T0J5;Q9VE80;D0IQ98	+	+	+	yes	+	+	-	no	-	-	-	no
Q8T0L3;O46111	+	+	+	yes	+	+	+	yes	+	+	+	yes
Q8T0M9;Q9VQ61;Q8IPY3;H5V866;E4NKM3	+	+	+	yes	+	+	+	yes	+	+	+	yes
Q8T0Q4	+	+	+	yes	+	+	+	yes	-	-	-	no
Q8T3U2	+	+	+	yes	+	+	+	yes	+	+	+	yes
Q8T4G5;Q8IQQ9	+	+	+	yes	+	+	-	no	-	-	-	no
Q8T6I0;Q8IGN0;Q8T8W3;Q94919	+	+	+	yes	+	+	-	no	-	-	-	no
Q8T8V5;P40796;M9ZVK8	+	+	+	yes	+	+	+	yes	-	-	-	no
Q8T9B4;Q9VLN6;B3DN70	+	+	+	yes	+	+	-	no	-	-	-	no
Q8T9B6	+	+	+	yes	-	-	+	no	-	-	-	no
Q8T9D5;Q7KV27;Q9VYD9	+	+	+	yes	-	-	+	no	-	-	-	no
Q8T9L0;Q9VQE0;Q8IHG0	+	+	-	no	+	+	-	no	-	-	-	no
Q94511;A4V449;Q6NP42;Q8MM97	+	+	+	yes	+	+	+	yes	-	+	-	no
Q94514	+	+	+	yes	+	+	+	yes	+	-	-	no
Q94516	+	+	+	yes	+	+	+	yes	+	+	+	yes
Q94518	+	+	+	yes	+	+	+	yes	+	+	+	yes
Q94522;M9MRQ9	+	+	+	yes	+	+	+	yes	-	+	+	yes
Q94523	+	+	+	yes	+	+	+	yes	-	-	-	no
Q94885	+	+	-	no	+	+	-	no	-	-	-	no
Q94920;M9PD75	+	+	+	yes	+	+	+	yes	+	+	+	yes
Q95029;C6SV44	+	+	+	yes	+	+	+	yes	+	+	+	yes
Q95083	+	+	+	yes	+	+	+	yes	+	+	+	yes
Q95RA9	+	+	+	yes	-	-	+	no	-	-	-	no
Q95S79;Q7JWQ7	+	+	+	yes	+	+	+	yes	-	-	-	no
Q95SA6;Q8SYQ2;A0AQH0	+	+	+	yes	+	+	+	yes	-	-	-	no
Q95WY3;Q8IGT5;Q961E2;Q95WY4	+	+	+	yes	+	+	+	yes	+	+	-	yes
Q960B0;A1ZAY8	+	+	+	yes	-	-	+	no	-	-	-	no
Q960D3;Q9VBX3;B5RIY3;Q6NLK4	+	+	+	yes	+	+	+	yes	+	+	+	yes
Q960K8;Q86BP1	+	+	+	yes	-	-	+	no	-	-	-	no
Q960M4	+	+	+	yes	+	+	+	yes	+	-	-	no
Q960Z0;A4V4A1	+	+	+	yes	-	-	+	no	-	-	-	no
Q961D7;Q058T6;Q9VC64	+	+	+	yes	-	-	-	no	-	-	-	no
Q961R8;Q9VUK8;C9QP25	+	+	+	yes	+	+	+	yes	+	+	+	yes

Q99323	+	+	+	yes	-	-	+	no	-	-	-	no
Q9GPN8;Q5LJP0	+	+	+	yes	+	+	-	no	-	-	-	no
Q9GQR4;Q9GQR6;Q9GQR5;Q9VMH3;Q9GQR3;Q8IPK0;D1Z391;M9NEA1	+	+	+	yes	-	-	+	no	-	-	-	no
Q9GU68	+	+	+	yes	+	+	+	yes	+	+	+	yes
Q9GU73;Q9VAN7;A5XD88;A5XD87;A5XD89;A5XD86;A5XD83	+	+	+	yes	+	+	+	yes	+	+	+	yes
Q9I7D3;E4NKG1	+	+	+	yes	+	+	+	yes	+	+	+	yes
Q9I7K0;B5RJ67	+	+	+	yes	-	-	-	no	-	-	-	no
Q9I7K6	+	+	+	yes	+	+	-	no	-	-	-	no
Q9I7M3;Q8INW5;Q9VIW5;C6TP94	-	+	+	no	-	-	+	no	-	-	-	no
Q9I7R0;C0PDF4;M9PEW1;C0PV64	+	+	+	yes	+	+	-	no	-	-	-	no
Q9I7T5;Q9VZL1	+	+	+	yes	-	-	+	no	-	-	-	no
Q9NH01	-	+	-	no	-	+	-	no	-	-	-	no
Q9NHD5	+	+	+	yes	-	-	-	no	-	-	-	no
Q9NHX7;Q7K148	+	+	+	yes	+	+	+	yes	-	-	-	no
Q9NJH0	+	+	+	yes	+	+	+	yes	+	+	+	yes
Q9NK57	+	+	-	no	-	-	-	no	-	-	-	no
Q9TVM2;Q8IH79	+	+	+	yes	+	+	-	no	-	-	-	no
Q9TW62;Q9VBI2;Q8IGD0;Q29QY5	+	+	+	yes	+	+	+	yes	-	-	-	no
Q9U3Z7;D2NUK9	+	+	+	yes	+	+	+	yes	+	+	+	yes
Q9U4L6;M9PGL7;H0RNC6;A1Z6L1	+	+	+	yes	+	+	+	yes	-	-	+	no
Q9U5D5;O18333	+	-	+	no	-	-	+	no	-	-	-	no
Q9U5L1;B5RIS4	+	+	+	yes	+	+	-	no	-	-	-	no
Q9U5W4;A0ANZ0;A0ANY9;Q9VM69	+	+	+	yes	+	+	+	yes	+	+	-	yes
Q9U7E6;Q8IPF5;Q9VLL3;Q9U7E7;Q86BM5;Q8SY32	+	+	+	yes	+	+	+	yes	-	+	-	no
Q9U915	+	+	+	yes	+	+	+	yes	-	-	-	no
Q9U916;Q53YH3;P29413	+	+	+	yes	+	+	+	yes	+	-	+	yes
Q9U9Q4	+	+	+	yes	+	+	+	yes	-	-	+	no
Q9V3A8	+	+	+	yes	+	+	-	no	-	-	-	no
Q9V3E7	+	+	+	yes	+	+	-	no	-	-	-	no
Q9V3G1;Q4V4W5	+	+	+	yes	+	+	+	yes	+	+	+	yes
Q9V3G7	+	+	+	yes	+	+	+	yes	+	+	+	yes
Q9V3H2	+	+	+	yes	+	+	+	yes	-	-	-	no
Q9V3I2	+	+	+	yes	+	+	+	yes	-	-	-	no
Q9V3J1;M9PDK5	+	+	+	yes	-	-	-	no	-	-	-	no
Q9V3J4;Q7KLW8	+	+	+	yes	+	+	-	no	-	-	-	no
Q9V3K3;M9PFN1	+	+	+	yes	+	+	+	yes	-	-	-	no
Q9V3P3	+	+	+	yes	+	+	+	yes	-	-	-	no
Q9V3P6	+	+	+	yes	+	+	+	yes	+	+	+	yes
Q9V3U2;M9PCI2	+	+	+	yes	-	-	-	no	-	-	-	no
Q9V3U6	+	+	+	yes	+	+	+	yes	+	+	+	yes
Q9V3V6;Q9XZC3	+	+	+	yes	+	+	+	yes	+	-	+	yes
Q9V3W0	+	+	+	yes	+	+	-	no	-	-	-	no
Q9V3W7	+	+	+	yes	+	+	+	yes	-	-	-	no
Q9V3Y4	+	+	-	no	+	+	-	no	-	-	-	no
Q9V3Z4	+	+	+	yes	+	+	+	yes	+	+	+	yes
Q9V405;E1UIA5;Q9VH79	+	+	+	yes	+	+	+	yes	-	-	-	no
Q9V411;C5WLT8;A0AVV0	+	+	-	no	-	-	-	no	-	-	-	no
Q9V427;E1JJF3	+	+	+	yes	+	+	-	no	-	-	-	no
Q9V434	+	+	+	yes	+	+	+	yes	-	-	-	no
Q9V436	+	+	+	yes	+	+	+	yes	-	-	+	no
Q9V438	+	+	+	yes	+	+	+	yes	-	-	+	no
Q9V455;Q9UB78;O76522	+	+	+	yes	+	+	+	yes	-	-	-	no
Q9V461;C6SUY3	-	+	+	no	-	+	-	no	-	-	-	no
Q9V496;L0MPS3;C7LAA3;Q1RKX2	+	+	+	yes	+	+	+	yes	+	+	+	yes
Q9V4E0;Q9VBR4;Q29QT0	+	+	+	yes	+	+	+	yes	-	-	-	no
Q9V4N3;E9P245;A8VEL0	+	+	+	yes	+	+	+	yes	-	-	-	no
Q9V597	+	+	+	yes	+	+	+	yes	+	+	+	yes
Q9V5C6	+	+	+	yes	+	+	+	yes	+	+	+	yes
Q9V5P6	+	+	+	yes	+	+	-	no	-	-	-	no
Q9V6K1	+	+	+	yes	+	+	+	yes	+	+	+	yes
Q9V7D2;X2JE14;Q9NEF6	+	+	+	yes	+	+	+	yes	-	-	-	no
Q9V7N5;B7YZI0	+	+	+	yes	+	+	-	no	-	-	-	no
Q9V895;B7YZR7;E1JGK5	+	+	+	yes	+	+	+	yes	-	-	-	no
Q9V8M5;E4NKM4	+	+	+	yes	+	+	-	no	+	-	-	no
Q9V948;E1JGN7;Q7KA80;A1ZBW1;A1ZBW0;Q7KHL0;D3DMD1;A1ZBW3;Q8SZZ6;Q8IGP8;A1ZBW4	+	+	+	yes	+	+	+	yes	-	-	-	no
Q9V9M7	+	+	+	yes	+	+	+	yes	+	+	+	yes
Q9V9S8	+	+	+	yes	+	+	-	no	-	-	-	no
Q9V9W2;Q9V9W3	+	+	+	yes	+	+	+	yes	+	+	+	yes
Q9VA18;Q24269	+	+	+	yes	-	-	-	no	-	-	-	no
Q9VA37	+	+	+	yes	-	-	-	no	-	-	-	no
Q9VA73;D0IQH2;Q8SY57	+	+	+	yes	+	+	+	yes	-	-	-	no
Q9VA83;Q9U7A3;Q9U4U2	+	+	+	yes	+	+	+	yes	+	+	+	yes
Q9VA91;Q8IMI7	+	+	+	yes	+	+	+	yes	+	+	+	yes
Q9VAC1	+	+	+	yes	+	+	+	yes	+	+	+	yes
Q9VAC4	+	+	+	yes	+	+	+	yes	+	-	+	yes
Q9VAM6	+	+	+	yes	+	+	+	yes	-	+	-	no
Q9VAN0	+	+	+	yes	+	+	+	yes	-	-	-	no
Q9VAP0;Q7KRW4;Q8SYT3	+	+	+	yes	-	-	-	no	-	-	-	no
Q9VAW5;F9W345;F9W325;E1NZB5	+	+	+	yes	+	+	+	yes	-	+	-	no
Q9VAY2;Q1RKX0	+	+	+	yes	+	+	+	yes	+	+	+	yes
Q9VB10	+	+	+	yes	-	-	+	no	-	-	-	no
Q9VB96;A9UNE9	+	+	+	yes	-	-	+	no	-	-	-	no
Q9VBN5	+	+	+	yes	+	+	+	yes	+	+	+	yes

Q9VBQ5	+	-	-	no	-	-	-	no	-	-	-	no
Q9VBU7;D3DMF2;Q8SWV7	+	+	+	yes	+	+	+	yes	-	+	+	yes
Q9VBU9	+	+	+	yes	+	+	+	yes	+	+	+	yes
Q9VC18;Q95RT1	+	+	+	yes	+	+	+	yes	-	+	-	no
Q9VC48	+	+	+	yes	+	+	-	no	-	-	-	no
Q9VC94	+	+	+	yes	-	-	+	no	-	-	-	no
Q9VCA5	+	+	+	yes	-	-	-	no	-	-	-	no
Q9VCA9	+	+	-	no	-	-	-	no	-	-	-	no
Q9VCK0;Q8MRJ8;Q9VGC7	+	+	+	yes	+	+	+	yes	-	-	-	no
Q9VCZ8;E1JIT4	+	+	+	yes	-	-	+	no	-	-	-	no
Q9VD29;Q95SY7;Q9NFN8	+	+	+	yes	+	+	+	yes	+	-	-	no
Q9VD51	+	+	+	yes	+	+	-	no	-	-	-	no
Q9VD58	+	+	+	yes	+	+	+	yes	-	-	-	no
Q9VD66	+	+	+	yes	+	+	-	no	-	-	-	no
Q9VDH8;Q7JYY0	+	+	+	yes	+	+	+	yes	+	-	-	no
Q9VDK7;Q5BHY9;Q95SX0	+	+	+	yes	-	-	+	no	-	-	-	no
Q9VDL2;Q86BR8	+	+	+	yes	-	-	-	no	-	-	-	no
Q9VDP9;Q7KSB5;C6SUZ3	+	+	+	yes	-	-	-	no	-	-	-	no
Q9VDT5	+	+	+	yes	-	-	+	no	-	-	-	no
Q9VDY8;Q8T3L2	+	+	+	yes	-	-	+	no	-	-	-	no
Q9VE75	+	+	+	yes	+	+	-	no	-	-	-	no
Q9VE79	+	+	+	yes	+	+	+	yes	+	+	+	yes
Q9VEA1;Q9NJB4	+	+	+	yes	+	+	+	yes	-	-	-	no
Q9VEB1	+	+	+	yes	+	+	+	yes	+	+	+	yes
Q9VEB3;B5RJN7	+	+	+	yes	-	-	-	no	-	-	-	no
Q9VEG6	+	+	+	yes	+	+	+	yes	+	-	+	no
Q9VEH0;Q960Y8;Q95R41	+	+	+	yes	+	+	+	yes	-	+	-	no
Q9VEJ0	+	+	+	yes	+	+	+	yes	+	+	+	yes
Q9VEN1;Q9GQV2;Q7KSF4;Q8T3K7;Q29R34;Q6NN54;C7LAD0;A4V310;C7LAD1;B7Z0L2	+	+	+	yes	+	+	+	yes	-	-	-	no
Q9VEP3;B5RJI8	+	+	+	yes	+	+	+	yes	-	-	-	no
Q9VEP6;Q95SP9	+	+	+	yes	+	+	+	yes	-	-	-	no
Q9VEY5	+	+	+	yes	-	-	-	no	-	-	-	no
Q9VEZ6;B7ZWP8;C1C588	+	+	+	yes	+	+	+	yes	-	-	-	no
Q9VFC8;D5SHR2	+	+	+	yes	-	-	+	no	-	-	-	no
Q9VFE4;X2JKU5;Q24186	+	+	+	yes	+	+	+	yes	-	+	-	no
Q9VFF0	+	+	+	yes	+	+	+	yes	+	+	+	yes
Q9VFQ9	+	+	+	yes	+	+	+	yes	+	+	+	yes
Q9VFT4;Q9NH72	+	+	+	yes	+	+	+	yes	+	+	+	yes
Q9VFV9	+	+	+	yes	+	+	+	yes	-	-	-	no
Q9VG16;Q9V3D8;Q9NHV8;Q8IH82	+	+	+	yes	+	+	+	yes	+	-	+	yes
Q9VG32;Q9NIW1;Q9VG31;Q9NIW2;Q9NIW0;Q2QBM1;H7BWU6;H7BWS7;H7BWS6;H7BWS3;H7BWS2;Q9U1J1;H7BWR6;H7BWU8;H7BWR4	+	+	+	yes	-	-	+	no	-	-	-	no
Q9VG69	+	+	+	yes	-	-	+	no	-	-	-	no
Q9VG70;Q7KSM5;Q7KJV5;A0ANW1;A0ANV9;A0ANV7;A0ANV4	+	+	+	yes	+	+	+	yes	-	-	-	no
Q9VG76	+	+	+	yes	+	+	-	no	-	-	-	no
Q9VG96	+	+	+	yes	+	+	+	yes	-	-	-	no
Q9VGA0;E4NKK7	+	+	+	yes	-	-	-	no	-	-	-	no
Q9VGH5;Q8IG99;Q8INJ6	+	+	+	yes	-	-	-	no	-	-	-	no
Q9VGM2;Q8IGA2;Q8INK3;Q8INK2	+	+	+	yes	+	+	+	yes	+	+	+	yes
Q9VGP6	+	+	+	yes	-	-	-	no	-	-	-	no
Q9VGQ1;U6SXS5;Q73HL2	+	+	+	yes	+	+	+	yes	-	-	+	no
Q9VGS2;S5PU30	+	+	+	yes	+	+	+	yes	+	+	+	yes
Q9VGW6	+	+	+	yes	-	-	-	no	-	-	-	no
Q9VGW7;C0MJ37;A0ANP8	+	+	+	yes	-	-	+	no	-	-	-	no
Q9VH07	+	+	+	yes	+	+	+	yes	-	-	-	no
Q9VH64	+	+	+	yes	+	+	+	yes	+	-	+	yes
Q9VH69;Q8IHD2;Q8IH91;I7CP40	+	+	+	yes	+	+	+	yes	+	+	+	yes
Q9VH95;E8NH46	+	+	+	yes	+	+	+	yes	+	+	+	yes
Q9VHA2;Q9VHA1;Q86P04	+	+	+	yes	+	+	+	yes	-	-	-	no
Q9VHC7;Q8MSI9	+	+	+	yes	+	+	-	no	-	-	-	no
Q9VHE5;Q9VBH8;Q8SYG0	+	+	+	yes	+	+	+	yes	+	+	+	yes
Q9VHI8;Q8INP8;Q8INP9;Q8MQQ5	+	+	+	yes	+	+	+	yes	-	-	-	no
Q9VHJ8;Q95U38;D6W4K6	+	+	+	yes	+	+	+	yes	+	+	-	yes
Q9VHL2	+	+	+	yes	+	+	+	yes	+	+	+	yes
Q9VHN6	+	+	+	yes	-	-	+	no	-	-	-	no
Q9VHP0;M9W6J0	+	+	+	yes	+	+	+	yes	+	+	+	yes
Q9VHR8;Q6BCZ0;Q8IGZ7	+	+	+	yes	+	+	+	yes	+	+	+	yes
Q9VHS2;D1Z3A3	+	+	+	yes	+	+	+	yes	-	-	-	no
Q9VHX4;B7Z0V3	+	+	+	yes	+	+	+	yes	-	-	-	no
Q9VHX9;Q8SYR7	+	+	+	yes	+	+	-	no	-	-	-	no
Q9VI10	+	+	+	yes	-	-	+	no	-	-	-	no
Q9VID9;Q8IH15;Q8SXA3	-	+	+	no	-	+	-	no	-	-	-	no
Q9VIE7;Q8WSN4;C4IXY5	-	+	+	no	-	+	-	no	-	-	-	no
Q9VIE8;Q8T4D6	+	+	+	yes	+	+	+	yes	+	+	+	yes
Q9VIH1;Q8SXG3;C0MJ04;A0ANL0;A0ANL2;A0ANL8	+	+	+	yes	+	+	+	yes	-	-	-	no
Q9VIQ8	+	+	+	yes	+	+	+	yes	-	-	-	no
Q9VIV8;A9UNF6;B3DNA4	+	+	+	yes	+	+	+	yes	-	-	+	no
Q9VJ19;Q8MT23;B5RIJ9	+	+	+	yes	+	+	+	yes	+	+	+	yes
Q9VJ31;C0MKB5;C0MKB4;A0APR0;A0APQ2;A0APQ1;A0APQ0;C0MKC0;C0MKB6;C0MKB3;A0APR1	+	+	+	yes	-	-	-	no	-	-	-	no
Q9VJ39;R4GRV7	+	+	+	yes	-	-	+	no	-	-	-	no
Q9VJ86;Q95NR4	+	+	+	yes	+	+	+	yes	-	-	-	no

Q9VJD1	+	+	+	yes	+	+	+	yes	-	-	+	no
Q9VJD4	+	+	+	yes	+	+	+	yes	-	-	-	no
Q9VJG0;O96794	+	+	+	yes	-	-	+	no	-	-	-	no
Q9VJJ0	+	+	+	yes	+	+	+	yes	-	+	+	yes
Q9VJZ4	+	+	+	yes	+	+	-	no	-	-	-	no
Q9VJZ6	+	+	+	yes	+	+	-	no	-	-	-	no
Q9VK60	+	+	+	yes	-	-	+	no	-	-	-	no
Q9VK69	+	+	+	yes	+	+	+	yes	+	+	+	yes
Q9VK85;M9PD08;P91944	+	+	+	yes	+	+	+	yes	-	-	-	no
Q9VKC1;Q8SYU2	+	+	-	no	-	-	-	no	-	-	-	no
Q9VKD3	+	+	+	yes	-	-	-	no	-	-	-	no
Q9VKI8	+	+	+	yes	+	+	+	yes	+	+	+	yes
Q9VKM3	+	+	+	yes	+	+	+	yes	+	+	+	yes
Q9VKM7;Q9VKM8	+	+	+	yes	+	+	+	yes	-	-	-	no
Q9VKV1;Q8IPB7;Q9VLI0	-	+	+	no	-	-	+	no	-	-	-	no
Q9VKW3	+	+	+	yes	-	-	+	no	-	-	-	no
Q9VKW5;Q961N7	+	+	+	yes	+	+	+	yes	-	-	-	no
Q9VKX2;Q8MQS7	+	+	+	yes	+	+	+	yes	+	+	+	yes
Q9VKY2;M9PB90;Q7KMK9	+	+	+	yes	-	-	-	no	-	-	-	no
Q9VKZ8	+	+	+	yes	+	+	+	yes	-	-	-	no
Q9VL16	+	+	+	yes	+	+	+	yes	-	-	-	no
Q9VL18	+	+	+	yes	+	+	+	yes	+	+	+	yes
Q9VL68	+	+	+	yes	+	+	+	yes	-	-	-	no
Q9VL69	+	+	+	yes	-	-	-	no	-	-	-	no
Q9VL70;Q9NIU5	+	+	+	yes	+	+	+	yes	+	+	+	yes
Q9VL89;O61540	+	+	+	yes	+	+	+	yes	+	+	+	yes
Q9VL96	+	+	-	no	+	+	-	no	-	-	-	no
Q9VLB7;Q24349	+	+	+	yes	+	+	+	yes	+	+	+	yes
Q9VLC5;Q4QPQ0;A9J7N9;B0F586;B0F584;B0F5A6;B0F585;B0F5B7;B0F593;B0F597;B0F590;E8NH42;B0F5B2;B0F5B3;B0F5B1;B0F5A7	+	+	+	yes	+	+	+	yes	+	+	+	yes
Q9VLK2;H0RNN8	+	+	+	yes	+	+	+	yes	-	-	-	no
Q9VLM5	+	+	+	yes	+	+	-	no	-	-	-	no
Q9VLT7;C5WLN8;Q29QX9	+	+	+	yes	-	-	-	no	-	-	-	no
Q9VLU0;M9PCH8	+	+	+	yes	+	+	+	yes	-	-	-	no
Q9VLV5	+	+	+	yes	+	+	-	no	-	-	-	no
Q9VMB9;Q86NN8	+	+	+	yes	+	+	+	yes	-	-	-	no
Q9VMI5	+	+	+	yes	+	+	-	no	-	-	-	no
Q9VMK3;Q8IH21	+	+	+	yes	+	+	+	yes	+	+	+	yes
Q9VMQ9	-	+	+	no	-	-	+	no	-	-	-	no
Q9VMS1	+	+	+	yes	+	+	-	no	-	-	-	no
Q9VMU4;M9MRF2	+	+	+	yes	-	-	-	no	-	-	-	no
Q9VMV5;O18407;C1C3H5	+	+	+	yes	+	+	+	yes	+	+	+	yes
Q9VMW4;Q9VMW2;E1JHT9;Q9VMW1;C6TP45	+	+	+	yes	+	+	+	yes	+	+	+	yes
Q9VN25	+	+	+	yes	+	+	+	yes	+	+	+	yes
Q9VN50	+	+	+	yes	+	+	+	yes	+	+	+	yes
Q9VNA5	+	+	+	yes	+	+	+	yes	-	-	+	no
Q9VNB9	+	+	+	yes	-	-	-	no	-	-	-	no
Q9VNE2	+	+	+	yes	+	+	+	yes	-	-	-	no
Q9VNE9	+	+	+	yes	+	+	+	yes	+	+	+	yes
Q9VNF3;Q9VNF4	+	+	+	yes	-	-	+	no	-	-	-	no
Q9VNH5	+	+	+	yes	-	-	-	no	-	-	-	no
Q9VNX4;Q8T3P0	+	+	+	yes	+	+	+	yes	-	-	-	no
Q9VP57	+	+	+	yes	-	-	+	no	-	-	-	no
Q9VPB3;Q8IPU3;H1UUD4;E4NKL2;Q9I7P8	+	+	+	yes	+	+	+	yes	-	-	+	no
Q9VPQ7;A7KX20;A7KX19	+	+	+	yes	-	-	-	no	-	-	-	no
Q9VPX7;Q7KN55;Q9VPX6	+	+	+	yes	+	+	+	yes	+	-	+	yes
Q9VQ29;H9XQA8;Q6NR57	+	+	+	yes	-	-	+	no	-	-	-	no
Q9VQ62	+	+	+	yes	-	-	-	no	-	-	-	no
Q9VQ94;M9PC99	+	+	+	yes	-	-	+	no	-	-	-	no
Q9VQF7	+	+	+	yes	+	+	+	yes	+	+	+	yes
Q9VQG4;Q9VM51;A0AQ00;A0APZ9;A0APZ8	+	+	+	yes	+	+	-	no	-	-	-	no
Q9VQL1;Q95TC0	+	+	+	yes	+	+	+	yes	+	+	-	yes
Q9VQL7;B7Z001;B6IDT3;Q7KML1;Q9VQL6;M9PB21	+	+	+	yes	+	+	+	yes	+	+	+	yes
Q9VQM2	+	+	+	yes	+	+	+	yes	-	-	-	no
Q9VQR2;A0AQ24	+	+	+	yes	+	+	+	yes	-	+	-	no
Q9VQR9;Q8SZL8	-	+	-	no	-	+	-	no	-	-	-	no
Q9VR96;E2DC56;E2DC17;E2DBW2;E2DBU7;E2DBU2;E2DBT6;E2DBT2;E2DBT1	+	+	+	yes	+	+	+	yes	-	-	+	no
Q9VRD6;Q6WAR9;A8JUT4;D0Z726;Q6WAR0;Q9VJ85;Q8I8H2;Q8I0N0;Q8I078	+	+	+	yes	+	+	+	yes	-	-	-	no
Q9VRD9	+	+	+	yes	+	+	+	yes	-	-	-	no
Q9VRJ9;B6IDQ5;M9PED6	+	+	+	yes	-	-	+	no	-	-	-	no
Q9VRL0	+	+	+	yes	+	+	+	yes	+	+	+	yes
Q9VRL1;Q8IGH9;Q8IQ53	+	+	+	yes	+	+	+	yes	-	-	-	no
Q9VRP2;Q5U0Z2	+	+	+	yes	+	+	+	yes	+	+	+	yes
Q9VRP3;Q7KMR7	+	+	+	yes	+	+	+	yes	-	-	-	no
Q9VRY0	-	-	+	no	-	-	+	no	-	-	-	no
Q9VS02;Q8SZM1	+	+	+	yes	+	+	-	no	-	-	-	no
Q9VS34;M9PHM6	+	+	+	yes	+	+	+	yes	+	+	+	yes
Q9VS47	+	+	+	yes	+	+	-	no	-	-	-	no
Q9VS52	+	+	+	yes	+	+	+	yes	-	-	-	no
Q9VSA3;G3KKP7	+	+	+	yes	+	+	+	yes	+	+	+	yes
Q9VSD6;Q86NK4;Q7KIF8;Q95RH0	+	+	+	yes	+	+	+	yes	+	+	+	yes
Q9VSD9;Q7KUA4;Q7KJV6	+	+	+	yes	+	+	-	no	-	-	-	no
Q9VSF3;M9PBW0	-	-	+	no	-	-	+	no	-	-	-	no
Q9VSL4;Q7K206	+	+	+	yes	+	+	-	no	-	-	-	no
Q9VSN9;Q6IDG2	-	+	+	no	-	-	+	no	-	-	-	no

Q9VSS1	+	+	+	yes	+	+	-	no	-	-	-	no
Q9VSS2	+	+	+	yes	+	+	+	yes	-	-	-	no
Q9VSU6	-	+	+	no	-	+	-	no	-	-	-	no
Q9VSY2;Q0E8G6	+	+	+	yes	-	-	-	no	-	-	-	no
Q9VT04;Q9VT03	+	+	+	yes	+	+	+	yes	-	-	-	no
Q9VT32;M9PEY1;M9PEP5;Q8IGE6;M9PHW7;M9PF46;Q9VT33;F0JAG1	+	+	+	yes	+	+	+	yes	-	-	-	no
Q9VT75	+	+	+	yes	+	+	+	yes	-	-	-	no
Q9VTE5;M9PF40	+	+	+	yes	+	+	+	yes	-	-	-	no
Q9VTF9	+	+	+	yes	-	-	+	no	-	-	-	no
Q9VTK9;M9PI17;Q8IQF8;M9PF61	+	+	+	yes	+	+	+	yes	+	+	+	yes
Q9VTP4;Q8IGE1;Q8IGJ3	+	+	+	yes	+	+	+	yes	+	+	+	yes
Q9VTU4	+	+	+	yes	+	+	+	yes	-	-	+	no
Q9VTW6	+	+	+	yes	-	-	-	no	-	-	-	no
Q9VTY2;Q8SXE8	+	+	+	yes	+	+	-	no	-	-	-	no
Q9VU35	+	+	+	yes	+	+	+	yes	+	+	+	yes
Q9VU68;C8VV50	+	+	+	yes	-	-	+	no	-	-	-	no
Q9VUB8	+	-	+	no	-	-	-	no	-	-	-	no
Q9VUC1;M9MSL3;Q9XZT5	+	+	+	yes	+	+	+	yes	+	+	+	yes
Q9VUJ1;O17312;O17311;Q9W470;C0ML85;B4F5B0;B4F5A2;Q86NT6	+	+	+	yes	+	+	+	yes	-	-	-	no
Q9VUQ5;M9PFK7;E5KZ94	+	+	+	yes	-	-	-	no	-	-	-	no
Q9VUY9	+	+	+	yes	+	+	-	no	-	-	-	no
Q9VUZ0	+	+	+	yes	-	-	+	no	-	-	-	no
Q9VUZ8	+	+	+	yes	-	-	+	no	-	-	-	no
Q9VV60	+	+	+	yes	+	+	+	yes	-	-	-	no
Q9VV75	+	+	+	yes	+	+	+	yes	+	+	+	yes
Q9VV82;Q8SWQ9;M9PFR8;B5RIL6;M9PFW8;Q9I7R5;Q8T094;M9WT69	+	+	+	yes	+	+	-	no	-	-	-	no
Q9VVA4;M9PCU8;M9NFH8;Q86PB9;Q7KUQ2	+	+	+	yes	+	+	+	yes	-	-	-	no
Q9VVA6	+	+	+	yes	-	-	+	no	-	-	-	no
Q9VVC5;Q8IQQ0;A8JNU6;Q8IGI6	+	+	+	yes	+	+	+	yes	-	-	-	no
Q9VVH5	-	-	+	no	-	-	+	no	-	-	-	no
Q9VVL7	+	+	+	yes	+	+	+	yes	+	+	-	yes
Q9VVU1;Q95U46	+	+	+	yes	+	+	+	yes	+	+	+	yes
Q9VVU2;Q7JRQ1	+	+	+	yes	+	+	+	yes	+	+	+	yes
Q9VW26	+	+	+	yes	+	+	-	no	-	-	-	no
Q9VW54	+	+	+	yes	+	+	+	yes	+	+	+	yes
Q9VW56;M9PD09	+	+	+	yes	-	-	-	no	-	-	-	no
Q9VW59	+	+	+	yes	-	-	+	no	-	-	-	no
Q9VW68;Q8MYV0;V5RLG1	+	+	+	yes	+	+	+	yes	+	+	+	yes
Q9VWA1;E1JI22	+	+	+	yes	-	-	-	no	-	-	-	no
Q9VWG3;M9NEQ9;D4G7H1;C0H6Z8;B7ZWP7;Q9VB14	+	+	+	yes	+	+	+	yes	+	+	+	yes
Q9VWH4	+	+	+	yes	+	+	+	yes	+	+	-	yes
Q9VWI2	+	+	+	yes	-	-	-	no	-	-	-	no
Q9VWT1;Q8IQX8;M9PHI2	+	+	+	yes	-	-	-	no	-	-	-	no
Q9VWV6;O97355;A9UNH0;F6J873;F6J859;F6J843;F6J836;F6J874;F6J842;F6J831;F6J817	+	+	+	yes	+	+	+	yes	+	+	-	yes
Q9VX77	-	-	+	no	-	-	+	no	-	-	-	no
Q9VX98	+	+	+	yes	-	-	+	no	-	-	-	no
Q9VXE0	+	+	+	yes	+	+	-	no	-	-	-	no
Q9VXF9	+	-	+	no	-	-	-	no	-	-	-	no
Q9VXI3;Q95TL8	+	+	+	yes	-	-	+	no	-	-	-	no
Q9VXI6	+	+	+	yes	-	-	+	no	-	-	-	no
Q9VXK0;M9PJP0	-	+	+	no	-	+	-	no	-	-	-	no
Q9VXK6;A1ZB32	+	+	+	yes	-	-	+	no	-	-	-	no
Q9VXK7;Q9VAK5;Q4V6N9	+	+	+	yes	-	-	-	no	-	-	-	no
Q9VXM4	+	+	+	yes	-	-	-	no	-	-	-	no
Q9VXN4	+	+	+	yes	+	+	-	no	-	-	-	no
Q9VXQ5	+	+	+	yes	+	+	+	yes	+	+	+	yes
Q9VXR5;Q8T9H2;A8E715	+	+	+	yes	+	+	+	yes	-	-	+	no
Q9VXY7;Q0KHS7;Q0KHS6;X2JKC1	+	+	+	yes	+	+	+	yes	+	+	+	yes
Q9VY87	+	+	+	yes	-	-	+	no	-	-	-	no
Q9VY91;B4YX27;B4YX26;F6J2G8;F6J2G6;F6J2G4;A9YHE2;A9YHD3;A9YHD2;F6J2H2	+	+	+	yes	-	-	+	no	-	-	-	no
Q9VYH8	+	+	+	yes	-	-	+	no	-	-	-	no
Q9VYI7;Q6NLI9;T2DHE1	+	+	+	yes	-	-	-	no	-	-	-	no
Q9VYV4;A9YGJ9;A9YGK3;F6J9S0;F6J9Q0	+	+	+	yes	-	-	-	no	-	-	-	no
Q9VYW3	+	+	+	yes	-	-	-	no	-	-	-	no
Q9VYW4;B4YWX9;B4YWX8;B4YWX7;B4YWX2;B4YWW9;B4YWW7;B4YWW6;B4YWW8;F6J1Q4;F6J1Q1;F6J1P5;F6J1P3;A9YGF4;A9YGF2;A9YGF0;A9YGE9;A9YGE8;A9YGE7;F6J9P5;F6J9N4;Q8MQQ7	-	+	+	no	-	+	-	no	-	-	-	no
Q9VYY2;B4F5V1;B4F5V0;B4F5U4	-	+	-	no	-	+	-	no	-	-	-	no
Q9VZ23;A4V4A5	+	+	+	yes	+	+	+	yes	+	+	+	yes
Q9VZ58;Q9VZ56;X2JB52	+	+	+	yes	-	-	-	no	-	-	-	no
Q9VZ64	-	+	+	no	-	+	-	no	-	-	-	no
Q9VZ69;Q8IR99;Q8IGK4;E1JJM0;Q0KHU2;M9NF14	+	+	+	yes	+	+	+	yes	-	+	-	no
Q9VZI1;M9PE30	+	+	+	yes	+	+	+	yes	+	+	+	yes
Q9VZL3;Q95R45;B6IDR5	+	+	+	yes	+	+	-	no	-	-	-	no

Q9VZQ8;Q8IRD4;Q8IRD3;Q86NS7	+	+	+	yes	+	+	+	yes	-	-	-	no
Q9VZS5;D1Z3A1;Q8MZI5	+	+	+	yes	+	+	+	yes	+	+	+	yes
Q9VZU4	+	+	+	yes	+	+	-	no	-	-	-	no
Q9VZU7	+	+	+	yes	-	-	+	no	-	-	-	no
Q9VZY0	+	+	+	yes	-	-	-	no	-	-	-	no
Q9W022	+	+	+	yes	-	-	+	no	-	-	-	no
Q9W028;B3DNA6;B3DNC1;A9UN41	+	+	+	yes	+	+	+	yes	-	-	-	no
Q9W029;D0IQ95	+	+	+	yes	+	+	+	yes	-	-	+	no
Q9W0B8;O77285	+	+	+	yes	+	+	+	yes	-	-	-	no
Q9W0E4;C8VUZ1;Q8IRH0;Q8IRH1;E8NH92;Q9GPG3;Q6AWR8	+	+	+	yes	+	+	+	yes	-	-	-	no
Q9W0H3	-	+	+	no	-	-	+	no	-	-	-	no
Q9W0M1;Q8T3H8	+	+	+	yes	+	+	-	no	-	-	-	no
Q9W0M4	+	+	+	yes	+	+	+	yes	-	-	-	no
Q9W0S7;Q8T0F1	+	+	+	yes	+	+	+	yes	+	+	+	yes
Q9W0Y1;Q2PE12	+	+	-	no	-	-	-	no	-	-	-	no
Q9W141;B5RJ63	+	+	+	yes	+	+	+	yes	+	+	+	yes
Q9W1B9;Q4V5X9	+	+	+	yes	+	+	+	yes	+	+	+	yes
Q9W1F7;Q8T3K9	+	+	+	yes	-	-	+	no	-	-	-	no
Q9W1G0;B3DMP7	+	+	+	yes	+	+	+	yes	-	-	-	no
Q9W1G7;Q24150	+	+	+	yes	+	+	+	yes	+	+	+	yes
Q9W1H8;O77466	+	+	+	yes	+	+	+	yes	+	+	+	yes
Q9W1I7	+	+	+	yes	+	+	+	yes	-	-	-	no
Q9W1N3	+	+	+	yes	-	-	+	no	-	-	-	no
Q9W1V3;E4NKN3	+	+	+	yes	+	+	+	yes	+	+	-	yes
Q9W227;E4NKJ7	+	+	+	yes	+	+	+	yes	+	+	+	yes
Q9W229	+	+	+	yes	+	+	+	yes	-	-	+	no
Q9W237	+	+	+	yes	+	+	+	yes	+	+	+	yes
Q9W2D9	+	+	+	yes	+	+	+	yes	+	-	-	no
Q9W2E8	+	+	+	yes	+	+	-	no	-	-	-	no
Q9W2M4;Q8MR08	+	+	+	yes	+	+	+	yes	-	-	+	no
Q9W2N0	+	+	+	yes	-	-	-	no	-	-	-	no
Q9W2U8	+	+	+	yes	-	-	-	no	-	-	-	no
Q9W2X6	+	+	+	yes	+	+	+	yes	+	+	+	yes
Q9W335	+	+	+	yes	-	-	+	no	-	-	-	no
Q9W392	+	+	+	yes	+	+	+	yes	+	+	+	yes
Q9W3E8	+	+	+	yes	-	-	-	no	-	-	-	no
Q9W3J5	+	+	+	yes	-	-	+	no	-	-	-	no
Q9W3M7	+	+	+	yes	-	-	+	no	-	-	-	no
Q9W3N1	+	+	+	yes	-	-	-	no	-	-	-	no
Q9W3N9	+	+	+	yes	+	+	-	no	-	-	-	no
Q9W3U0	+	+	+	yes	-	-	+	no	-	-	-	no
Q9W3Y3	+	+	+	yes	+	+	+	yes	-	-	-	no
Q9W401;C6TP50;Q8T9J9	+	+	+	yes	+	+	+	yes	+	+	-	yes
Q9W402	+	+	+	yes	-	-	+	no	-	-	-	no
Q9W414;Q8SZ19;C1C3F8;Q9U7A2;Q9VTQ9	+	+	+	yes	+	+	+	yes	+	+	+	yes
Q9W445	+	+	+	yes	-	-	+	no	-	-	-	no
Q9W499;Q8MT59;C6SV02	+	+	+	yes	+	+	+	yes	-	-	-	no
Q9W4H6;Q7KVX1;Q7YU05;F6J1A7	+	+	+	yes	+	+	+	yes	-	-	+	no
Q9W4J4	+	+	+	yes	+	+	+	yes	-	-	-	no
Q9W4K0	+	+	+	yes	+	+	-	no	-	-	-	no
Q9W4M3;Q9GTX9;A8E6S7;A8E6U1;A8E6T3;A8E6S0	+	+	+	yes	+	+	+	yes	-	-	-	no
Q9W4M4	+	+	+	yes	+	+	-	no	+	-	-	no
Q9W4X7	+	+	+	yes	+	+	+	yes	-	-	-	no
Q9W5A7;Q8SYB4;Q9NF34	+	+	+	yes	-	-	+	no	-	-	-	no
Q9W5N2	+	+	+	yes	+	+	+	yes	+	+	+	yes
Q9W5W8	+	+	+	yes	+	+	-	no	-	-	-	no
Q9XTL9;A4UZZ4	+	+	+	yes	+	+	+	yes	+	+	+	yes
Q9XY35;B9EQT5;Q8SY81	+	+	+	yes	-	-	-	no	-	-	-	no
Q9XYN7;C0MJU2	+	+	+	yes	+	+	+	yes	-	-	-	no
Q9XYX0;Q9VZ96;M9NF15;M9PBP8;M9NFN0;M9PE65	+	+	+	yes	+	+	-	no	-	-	-	no
Q9XYZ5	+	+	-	no	-	-	-	no	-	-	-	no
Q9XZ03;M9PFR6	+	+	+	yes	+	+	+	yes	-	-	-	no
Q9XZ57;Q7JZD3;D3DMK3;D6W4V6;Q6NNE2;A1Z6P3;D5SHT9	+	+	+	yes	+	+	+	yes	-	-	-	no
Q9XZ61	+	+	+	yes	+	+	+	yes	-	-	+	no
Q9XZE4;Q0E8E8;Q8SYD4;Q7JUS9	+	+	+	yes	+	+	+	yes	+	+	+	yes
Q9XZH6	+	+	+	yes	-	-	+	no	-	-	-	no
Q9XZJ4;E1JGZ9	+	+	+	yes	+	+	+	yes	+	+	+	yes
Q9XZU1	+	+	+	yes	+	+	-	no	-	-	-	no
Q9Y0Y5	+	+	+	yes	-	-	+	no	-	-	-	no
Q9Y105	+	+	+	yes	+	+	+	yes	-	-	-	no
R9Q794;Q9W5R8;Q6NL81;Q9NIU2;Q6NMY6	+	+	+	yes	+	+	+	yes	+	+	+	yes
T2FFB7;Q9VLM8;Q8T9G8;K7ZI11;K7ZDS0;K7ZI63	+	+	+	yes	+	+	+	yes	+	+	+	yes
T2FFJ2;Q9VQR8;Q8MRF8	+	+	+	yes	+	+	-	no	-	-	-	no
T2GFI6;Q960E6;Q0E993	+	+	+	yes	+	+	+	yes	-	-	-	no
U6SWA5;Q73HC4	+	+	+	yes	-	-	-	no	-	-	-	no
U6SX25;Q73I71;Q2MZ27	+	+	+	yes	+	+	-	no	-	-	-	no
U6SXX3;Q73H52	+	+	+	yes	+	+	-	no	-	-	-	no
U6SZA1	+	+	+	yes	+	+	-	no	-	-	-	no
X2J4W8;Q9V447	+	+	+	yes	+	+	+	yes	-	-	-	no
X2J5E8;Q9VL78	+	+	+	yes	+	+	+	yes	+	+	+	yes
X2J5G6;P32100	+	+	+	yes	+	+	+	yes	+	+	+	yes
X2J5T3;O18334;F6J915;Q9W3Q0;Q8SYS5	+	+	+	yes	-	-	-	no	-	-	-	no
X2J6D4;P84029	+	+	+	yes	+	+	+	yes	+	-	+	yes

X2J7U0;Q9VKM1;A0A075BLT4;A0A075BLT1;A0A075BJU9;A0A075BJU3;A0A075BJD6;A0A075BJD5;A0A075BJ92;A0A075BJ89	+	+	+	yes	-	-	+	no	-	-	-	no
X2J950;Q03334	+	+	+	yes	+	+	+	yes	+	+	+	yes
X2J9F7;Q9VKI3	+	+	+	yes	-	-	-	no	-	-	-	no
X2J9P9;Q9VMA3;Q4V6K0	+	+	+	yes	+	+	+	yes	+	+	+	yes
X2JAB9;O46037;Q24584	-	+	-	no	-	+	-	no	-	-	-	no
X2JAI4;Q9XYU1	+	+	+	yes	-	-	+	no	-	-	-	no
X2JAW6;P15215	+	+	+	yes	+	+	+	yes	-	-	-	no
X2JB24;Q9VU58	+	+	+	yes	+	+	+	yes	-	-	-	yes
X2JB25;P02844;B5RJK7	+	+	+	yes	+	+	+	yes	+	+	+	yes
X2JB48;Q26365;D1Z385;O62526	+	+	+	yes	+	+	+	yes	+	+	+	yes
X2JB87;Q9Y1A3	+	-	+	no	-	-	-	no	-	-	-	no
X2JC31;P29742	+	+	+	yes	+	+	+	yes	+	+	+	yes
X2JC35;P49630	+	+	+	yes	+	+	+	yes	+	+	+	yes
X2JC80;P38979;C6SV50	+	+	+	yes	+	+	+	yes	+	+	+	yes
X2JC82;P02518	+	+	+	yes	+	+	+	yes	+	+	+	yes
X2JC94;O46084	+	+	+	yes	-	-	+	no	-	-	-	no
X2JCE7;Q9VX03;Q1RL06;Q8IQZ1;A3FP35;Q86B44	-	+	-	no	-	+	-	no	-	-	-	no
X2JCI6;P47949;P47948	+	+	+	yes	+	+	+	yes	-	-	-	no
X2JCP8;P10987;P02572	+	+	+	yes	+	+	+	yes	+	+	+	yes
X2JCS6;P46223	+	+	+	yes	+	+	+	yes	+	+	+	yes
X2JCX8;P14130	+	+	+	yes	+	+	+	yes	+	+	+	yes
X2JCY2;Q9VUL1	+	+	+	yes	-	-	-	no	-	-	-	no
X2JD55;P02843;Q29QE3	+	+	+	yes	+	+	+	yes	+	+	+	yes
X2JDI1;P23696	+	+	+	yes	-	-	+	no	-	-	-	no
X2JDP6;Q8T079;M9MRG2	+	+	+	yes	-	-	-	no	-	-	-	no
X2JDR5;P47938	+	+	+	yes	+	+	+	yes	-	+	+	no
X2JDU0;P50882	+	+	+	yes	+	+	+	yes	+	+	+	yes
X2JE06;Q9VJY6;Q8SY98	+	+	+	yes	+	+	+	yes	+	+	+	yes
X2JE34;Q8IR13;Q9VXF8;E8NH88;Q7KUX7;Q29R50;X2JC79;Q26272	+	+	+	yes	+	+	+	yes	+	+	-	yes
X2JEB6;Q9W303;A1A729	+	+	-	no	+	+	-	no	-	-	-	no
X2JEJ0;Q9W3F6;A8JV22;A8JV24	+	+	+	yes	-	-	-	no	-	-	-	no
X2JEM4;Q9W334	+	+	+	yes	+	+	+	yes	+	+	+	yes
X2JEX8;P06607;D3DMQ1;Q4V524;Q4V530;Q4V566;Q4V563;D5SHM4;Q9VPH3;M9NFL7;Q9VX69;Q8SXG0	+	+	+	yes	+	+	+	yes	+	+	+	yes
X2JF40;Q27580;M9PHN8	+	+	+	yes	+	+	+	yes	+	+	+	yes
X2JF59;Q9V3P0;F6J6B9	+	+	+	yes	+	+	+	yes	+	+	+	yes
X2JFG0;P05990;Q1RKZ5;A6YH32	+	+	+	yes	+	+	+	yes	-	-	-	no
X2JFR6;P41374	+	+	+	yes	+	+	+	yes	+	+	+	yes
X2JGG6;P02517	+	+	+	yes	+	+	+	yes	+	+	+	yes
X2JGM9;P41042	+	+	+	yes	+	+	+	yes	+	+	+	yes
X2JGP4;P54399	+	+	+	yes	+	+	+	yes	+	+	+	yes
X2JH42;Q05825;L0MQ04;Q6W4M9;Q6W4K9;Q8IM98;Q8T4C4;U6SYW6;Q73IG3	+	+	+	yes	+	+	+	yes	+	+	+	yes
X2JIQ5;Q9W3W8	+	+	+	yes	+	+	+	yes	+	+	-	yes

Table S2

Protein IDs	Protein detection, according to absolute quantification (iBAQ)				Protein quality, according to relative quantification (LFQ)				Protein reliablity, according to assessment criteria			
	Dsim Cured	Dsim *w*Ri	Dsim *w*Mel	detected in all?	Dsim Cured	Dsim *w*Ri	Dsim *w*Mel	high quality data in all?	Dsim Cured	Dsim *w*Ri	Dsim *w*Mel	reliable overall?
A0A075BJ95;B4Q9X1;A0A075BJV4;A0A075BJD8;A0A075BI48;A0A075BI44;K7WKM7;K7XHU8;K7XHU3;A0A075BLT5;K7WS48;K7WKN1;K7X4Z7;K7XHU0;K7WPZ4;K7WS50;K7X4Z3;K7WPZ7	+	+	+	yes	+	+	+	yes	+	-	-	no
A0ANM2	+	+	+	yes	+	+	+	yes	-	-	-	no
A0ANQ8;B4QUP6	+	+	+	yes	+	-	+	no	-	-	-	no
A0ANU2;B4Q984	+	-	+	no	+	-	-	no	-	-	-	no
A0ANX6	+	+	+	yes	-	+	+	no	-	-	-	no
A0AP03	+	+	+	yes	+	-	-	no	-	-	-	no
A0APF0;B4QBB9	+	+	+	yes	+	+	+	yes	+	+	-	yes
A0API2	+	+	+	yes	+	+	+	yes	-	+	+	yes
A0AQ35;B4Q9K7	+	+	+	yes	-	+	-	no	-	-	-	no
A0AQ71	+	+	+	yes	+	+	-	no	-	-	-	no
A2TDX4	+	+	+	yes	+	+	+	yes	-	-	-	no
A4ULX4;B4QM69	+	+	+	yes	+	+	+	yes	-	-	-	no
A4ULY9	+	+	+	yes	+	+	+	yes	+	+	-	yes
A4UM14	+	+	+	yes	-	+	-	no	-	-	-	no
A4UM25;B4Q4T7	+	+	+	yes	-	+	-	no	-	-	-	no
A4UM48;Q3YMV1;A4ULW4;A4UM46;A4UM45	+	+	+	yes	+	+	+	yes	-	-	-	no
A5XCI5;A5XCH6;A5XCI1;U6SZR0;P62422;C0R4J5	+	+	+	yes	+	+	+	yes	+	-	+	yes
A5XCP5;A5XCP2;A5XCP1;B4QMR8	+	-	+	no	+	-	-	no	-	-	-	no
A5XCU4	+	+	+	yes	+	+	+	yes	-	-	-	no
A5XCX1;B4QES8;B4R5J0	+	+	+	yes	+	+	+	yes	+	+	+	yes
A5XD63;B4Q767	+	+	+	yes	+	+	+	yes	+	+	+	yes
A5XD93;A5XDA3	+	+	+	yes	+	+	+	yes	+	+	+	yes
A9YHJ6;B4Q5K2	+	+	+	yes	+	+	+	yes	-	-	-	no
B2ZI48;B4QC68	+	+	+	yes	+	+	+	yes	-	-	-	no
B2ZIA0	+	+	+	yes	+	+	+	yes	+	+	+	yes
B4F5V6	+	+	+	yes	-	+	-	no	-	-	-	no
B4NRX0	+	+	+	yes	+	+	+	yes	-	-	-	no
B4NRX6	+	+	+	yes	+	-	-	no	-	-	-	no
B4NRX8	+	+	+	yes	+	+	+	yes	+	+	+	yes
B4NS12	+	+	+	yes	-	+	-	no	-	-	-	no
B4NS73	+	+	+	yes	-	-	+	no	-	-	-	no
B4NSB5;B4QG84	+	-	+	no	+	-	-	no	-	-	-	no
B4NSE1	+	+	+	yes	+	-	-	no	-	-	-	no
B4NSF4	+	+	+	yes	+	-	+	no	-	-	-	no
B4NSG8;B4QC90	+	+	+	yes	+	+	+	yes	+	+	+	yes
B4NSL8	+	+	+	yes	+	+	+	yes	+	+	+	yes
B4NT60	+	+	+	yes	+	+	+	yes	-	-	-	no
B4NT61	+	+	-	no	+	-	-	no	-	-	-	no
B4NT68;Q8WP82	+	+	+	yes	+	+	+	yes	-	-	-	no
B4NT86	+	+	+	yes	+	-	-	no	-	-	-	no
B4NTA6;B4R1B4	+	+	+	yes	-	+	-	no	-	-	-	no
B4NTC0	+	+	+	yes	+	+	+	yes	+	+	+	yes
B4NTD4	+	+	+	yes	+	+	+	yes	+	+	+	yes
B4NTI4	+	+	+	yes	+	+	+	yes	+	+	+	yes
B4NTI5	+	+	+	yes	+	-	+	no	-	-	-	no
B4NTL1	+	+	+	yes	-	+	-	no	-	-	-	no
B4NTM4;B4R5J9	+	+	+	yes	-	-	+	no	-	-	-	no
B4NTM6	+	+	+	yes	+	+	-	no	-	-	-	no
B4NTN2	+	+	+	yes	-	+	-	no	-	-	-	no
B4NTR2	+	+	+	yes	+	+	+	yes	+	+	+	yes
B4NTW6	+	+	+	yes	+	-	+	no	-	-	-	no
B4NTY9	+	+	+	yes	+	+	+	yes	-	-	-	no
B4NU29	+	+	+	yes	-	-	+	no	-	-	-	no
B4NU39;B4F5E9	+	+	+	yes	-	+	-	no	-	-	-	no
B4NU45	+	+	+	yes	+	+	+	yes	+	-	-	no
B4NU46	+	+	+	yes	+	+	+	yes	+	+	+	yes
B4NU57	+	-	+	no	-	-	+	no	-	-	-	no
B4NU67	+	+	+	yes	+	+	+	yes	+	+	+	yes
B4NU78	+	+	+	yes	+	+	+	yes	-	-	-	no
B4NU90	+	+	+	yes	+	-	-	no	-	-	-	no
B4NUA4	+	+	+	yes	+	+	+	yes	+	+	+	yes
B4NUD3	+	+	+	yes	-	-	+	no	-	-	-	no
B4NUF0	+	+	+	yes	+	+	+	yes	-	-	-	no
B4NUF5	+	+	+	yes	-	+	-	no	-	-	-	no
B4NUJ8;Q9N6A6;Q9NGF2	+	+	+	yes	-	-	+	no	-	-	-	no
B4NUM4	+	+	+	yes	-	+	-	no	-	-	-	no
B4NV70	+	+	+	yes	-	+	+	no	-	-	-	no
B4NVF9;B4NUY1	+	+	+	yes	+	+	+	yes	+	+	+	yes
B4NVJ4;Q76N01;O02648;B4Q3I4;B4NVS5;B4NVK9	+	+	+	yes	+	+	+	yes	+	+	+	yes
B4NVN2	+	+	+	yes	-	+	-	no	-	-	-	no
B4NVN9	+	+	+	yes	-	-	+	no	-	-	-	no
B4NW16;A0AQI1	+	+	+	yes	+	+	+	yes	-	-	-	no
B4Q354	+	+	+	yes	+	+	+	yes	+	+	+	yes
B4Q366	+	+	+	yes	+	+	+	yes	-	+	-	no
B4Q3C4	+	-	+	no	+	-	-	no	-	-	-	no
B4Q3I2	+	+	+	yes	-	+	-	no	-	-	-	no
B4Q3I6	+	+	+	yes	+	+	+	yes	-	+	-	no
B4Q3M1	+	+	+	yes	+	-	-	no	-	-	-	no
B4Q3P3	+	+	+	yes	+	-	-	no	-	-	-	no
B4Q3Q6	+	+	+	yes	+	+	+	yes	-	-	+	no

B4Q3T5	+	+	+	yes	-	+	-	no	-	-	-	no
B4Q400;B4QUK9	+	+	+	yes	+	+	+	yes	+	+	+	yes
B4Q441;Q8T8L7;O76807	+	-	+	no	+	-	-	no	-	-	-	no
B4Q448	+	+	+	yes	+	+	+	yes	+	-	-	no
B4Q453	+	+	+	yes	+	+	+	yes	+	+	+	yes
B4Q482	+	+	+	yes	+	+	+	yes	+	+	+	yes
B4Q492	+	+	+	yes	+	+	+	yes	-	-	-	no
B4Q4A5;B4Q4A6;Q8MXF6	+	+	+	yes	+	+	+	yes	+	+	+	yes
B4Q4A8	+	-	-	no	+	-	-	no	-	-	-	no
B4Q4F1	+	+	+	yes	+	+	+	yes	+	+	+	yes
B4Q4F6	+	+	+	yes	+	+	+	yes	+	+	+	yes
B4Q4F9	+	+	+	yes	+	+	-	no	-	-	-	no
B4Q4H8	+	+	+	yes	+	+	+	yes	-	-	-	no
B4Q4H9	+	+	+	yes	+	-	-	no	-	-	-	no
B4Q4N5	+	+	+	yes	+	+	+	yes	+	+	-	yes
B4Q4S2	+	+	+	yes	+	-	+	no	-	-	-	no
B4Q4S5	+	-	-	no	+	-	-	no	-	-	-	no
B4Q4S9	+	-	+	no	+	-	-	no	-	-	-	no
B4Q4T3	+	+	+	yes	+	+	+	yes	+	+	+	yes
B4Q4X0	+	+	+	yes	+	+	+	yes	+	+	+	yes
B4Q4Z9	+	+	+	yes	-	+	-	no	-	-	-	no
B4Q501	+	+	+	yes	+	+	+	yes	+	+	+	yes
B4Q520	+	+	+	yes	-	+	-	no	-	-	-	no
B4Q595	+	+	+	yes	+	+	+	yes	+	-	-	no
B4Q5A6	+	+	+	yes	+	+	+	yes	-	+	+	yes
B4Q5L9	+	+	+	yes	+	+	+	yes	-	-	-	no
B4Q5M3	+	+	+	yes	+	-	-	no	-	-	-	no
B4Q5P6	+	+	+	yes	+	-	+	no	-	-	-	no
B4Q5P7	+	+	+	yes	-	+	-	no	-	-	-	no
B4Q5U0	+	+	+	yes	-	-	+	no	-	-	-	no
B4Q5U2;F6JMF8;F6JGS8;<br>A9YGS6;B5LFR9;B5LFR8;<br>B4R3L2;REV__B4QEY7	+	+	+	yes	+	+	+	yes	+	+	+	yes
B4Q5U3	+	+	+	yes	-	+	-	no	-	-	-	no
B4Q5Z8	+	+	+	yes	+	+	+	yes	+	+	-	yes
B4Q623;Q86C07;Q86C06;<br>Q86C05	+	+	+	yes	+	+	+	yes	+	+	+	yes
B4Q635	+	+	+	yes	+	-	-	no	-	-	-	no
B4Q638	+	+	+	yes	+	+	+	yes	+	+	+	yes
B4Q652	+	+	+	yes	-	+	+	no	-	-	-	no
B4Q688	+	+	+	yes	+	-	-	no	-	-	-	no
B4Q696	+	+	+	yes	+	+	-	no	-	-	-	no
B4Q6D2	+	-	-	no	+	-	-	no	-	-	-	no
B4Q6N7	+	+	+	yes	+	+	+	yes	-	-	-	no
B4Q6P8;A0ANQ9	+	+	+	yes	-	+	-	no	-	-	-	no
B4Q6R4	+	-	+	no	+	-	-	no	-	-	-	no
B4Q6R5	+	+	+	yes	+	+	+	yes	+	+	+	yes
B4Q6R7	+	+	+	yes	+	+	+	yes	+	+	+	yes
B4Q6S2	+	+	+	yes	+	+	+	yes	+	-	+	yes
B4Q6S5	+	+	+	yes	+	-	-	no	-	-	-	no
B4Q6T6	+	+	+	yes	-	-	+	no	-	-	-	no
B4Q6U0	+	+	+	yes	+	+	+	yes	-	-	-	no
B4Q6V5	+	+	+	yes	-	+	-	no	-	-	-	no
B4Q6X9	+	+	+	yes	+	+	-	no	-	-	-	no
B4Q713	+	+	+	yes	+	+	+	yes	-	-	-	no
B4Q764	+	+	+	yes	+	+	+	yes	-	-	-	no
B4Q782	-	+	+	no	-	+	-	no	-	-	-	no
B4Q7E9	+	+	+	yes	+	+	+	yes	-	-	-	no
B4Q7F4	+	+	+	yes	+	+	+	yes	+	+	+	yes
B4Q7G2	+	+	+	yes	+	+	+	yes	-	+	+	yes
B4Q7G3	+	+	+	yes	+	+	+	yes	+	+	+	yes
B4Q7H1	+	+	+	yes	-	-	+	no	-	-	-	no
B4Q7L0	+	+	+	yes	+	+	+	yes	+	+	+	yes
B4Q7M5	+	+	+	yes	+	+	+	yes	+	+	+	yes
B4Q7N8	+	+	+	yes	+	+	+	yes	+	+	+	yes
B4Q7R7	+	+	+	yes	+	+	+	yes	+	+	+	yes
B4Q7S8	+	+	+	yes	+	+	+	yes	+	+	+	yes
B4Q7T9	+	+	+	yes	+	+	+	yes	+	+	+	yes
B4Q7U5	+	+	+	yes	+	+	+	yes	+	+	+	yes
B4Q7V2	+	+	+	yes	+	+	+	yes	+	+	+	yes
B4Q7Y5	+	+	+	yes	-	-	+	no	-	-	-	no
B4Q7Y9	+	+	+	yes	+	+	+	yes	-	-	-	no
B4Q7Z2	+	-	-	no	+	-	-	no	-	-	-	no
B4Q805	+	+	+	yes	-	+	-	no	-	-	-	no
B4Q831	+	+	+	yes	+	-	+	no	-	-	-	no
B4Q872	+	+	+	yes	+	+	+	yes	-	+	+	yes
B4Q884	+	+	+	yes	+	+	+	yes	+	+	+	yes
B4Q889	+	+	+	yes	+	+	+	yes	+	+	+	yes
B4Q894	+	+	+	yes	+	+	+	yes	-	-	-	no
B4Q895	+	+	+	yes	-	+	-	no	-	-	-	no
B4Q896;B4Q897	+	+	+	yes	+	+	+	yes	-	-	-	no
B4Q8B3	+	+	+	yes	+	+	+	yes	+	+	+	yes
B4Q8G2	+	+	+	yes	+	+	+	yes	+	-	-	no
B4Q8G4	+	+	+	yes	+	-	-	no	-	-	-	no
B4Q8J5	+	+	+	yes	+	+	+	yes	+	+	+	yes
B4Q8K4;A0AQ09	+	+	+	yes	+	-	-	no	-	-	-	no
B4Q8K5	+	+	+	yes	+	-	-	no	-	-	-	no
B4Q8M1	+	+	+	yes	-	+	+	no	-	-	-	no
B4Q8N5	+	+	+	yes	+	+	+	yes	-	+	+	no
B4Q8Q1	+	+	+	yes	+	+	+	yes	+	+	+	yes
B4Q8Q8	+	+	+	yes	+	+	+	yes	+	+	+	yes
B4Q8R2	+	+	+	yes	+	+	+	yes	+	+	+	yes
B4Q8R4	+	+	+	yes	+	+	+	yes	+	+	+	yes
B4Q8R9	+	+	+	yes	+	+	+	yes	-	-	-	no
B4Q8T6	+	+	+	yes	+	+	+	yes	-	+	-	no
B4Q8T7	+	-	+	no	+	-	-	no	-	-	-	no

B4Q8U0	+	+	+	yes	+	-	-	no	-	-	-	no
B4Q8Z5	+	+	+	yes	-	+	+	no	-	-	-	no
B4Q903	+	+	+	yes	+	-	-	no	-	-	-	no
B4Q922	+	+	+	yes	+	+	+	yes	-	-	-	no
B4Q930;B4NUW8	+	+	+	yes	+	-	-	no	-	-	-	no
B4Q938	+	+	+	yes	+	-	-	no	-	-	-	no
B4Q956	+	+	+	yes	-	+	-	no	-	-	-	no
B4Q975	+	+	-	no	-	+	-	no	-	-	-	no
B4Q980	+	+	+	yes	+	+	+	yes	-	-	-	no
B4Q988	+	+	+	yes	+	+	+	yes	+	+	+	yes
B4Q995	+	+	+	yes	+	+	+	yes	+	+	-	yes
B4Q9A0	+	+	+	yes	-	+	-	no	-	-	-	no
B4Q9E1	+	+	+	yes	+	+	+	yes	+	+	+	yes
B4Q9F4;B1Q013	+	+	+	yes	+	+	+	yes	-	-	-	no
B4Q9G9	+	+	+	yes	+	+	+	yes	+	+	+	yes
B4Q9H2	+	+	+	yes	+	+	+	yes	-	-	-	no
B4Q9L5	+	+	+	yes	-	+	-	no	-	-	-	no
B4Q9U9	+	+	+	yes	+	+	+	yes	+	+	+	yes
B4Q9W3	+	+	+	yes	-	+	-	no	-	-	-	no
B4Q9W8	+	+	+	yes	+	+	+	yes	+	+	-	yes
B4Q9X4;K7XHS0;K7WPY8	+	+	+	yes	+	-	-	no	-	-	-	no
B4Q9Y2	+	+	+	yes	-	+	-	no	-	-	-	no
B4Q9Y6	+	+	+	yes	+	+	+	yes	-	+	+	yes
B4Q9Y7	+	+	+	yes	+	+	+	yes	-	-	-	no
B4QA80	+	+	+	yes	+	+	+	yes	+	+	+	yes
B4QA88	+	+	+	yes	+	+	+	yes	+	+	+	yes
B4QA91	+	+	+	yes	+	+	+	yes	+	+	+	yes
B4QAC2	+	+	+	yes	+	+	+	yes	+	+	+	yes
B4QAF2;Q7YZF1;Q7YZF0; A2TDT5	+	+	+	yes	+	+	+	yes	-	+	-	no
B4QAI9	+	+	+	yes	+	+	+	yes	+	-	-	no
B4QAJ9	+	+	+	yes	+	-	-	no	-	-	-	no
B4QAK0	+	+	+	yes	+	-	-	no	-	-	-	no
B4QAN0	+	+	+	yes	+	+	+	yes	-	-	-	no
B4QAN5	+	+	+	yes	+	+	+	yes	-	-	-	no
B4QAQ1	+	+	-	no	+	-	-	no	-	-	-	no
B4QAS8	+	+	+	yes	+	+	+	yes	+	+	-	yes
B4QAT1	+	+	+	yes	-	+	-	no	-	-	-	no
B4QB04	+	+	+	yes	+	-	+	no	-	-	-	no
B4QB11	+	+	+	yes	+	+	+	yes	+	+	+	yes
B4QB65	+	+	-	no	-	+	-	no	-	-	-	no
B4QB88	+	+	+	yes	+	+	+	yes	-	-	-	no
B4QB93	+	+	+	yes	+	+	+	yes	+	+	+	yes
B4QB99	+	+	+	yes	-	+	-	no	-	-	-	no
B4QBB4	+	+	+	yes	+	+	+	yes	+	-	-	no
B4QBB5	+	+	+	yes	-	+	-	no	-	-	-	no
B4QBB6	+	+	+	yes	+	+	+	yes	-	-	-	no
B4QBE3	+	+	+	yes	+	+	+	yes	+	+	+	yes
B4QBE5	+	+	-	no	+	-	-	no	-	-	-	no
B4QBE6	+	+	+	yes	+	+	+	yes	+	-	-	no
B4QBE8	+	+	+	yes	+	-	+	no	-	-	-	no
B4QBH5	+	+	+	yes	+	+	+	yes	+	+	+	yes
B4QBH8	+	+	+	yes	+	-	-	no	-	-	-	no
B4QBI3	+	+	+	yes	+	+	+	yes	+	+	+	yes
B4QBI4	+	+	+	yes	+	+	+	yes	+	+	+	yes
B4QBJ6	+	+	+	yes	+	+	+	yes	+	+	+	yes
B4QBK5	+	+	+	yes	+	+	+	yes	+	+	-	yes
B4QBK9	+	+	+	yes	-	-	+	no	-	-	-	no
B4QBL5	+	+	+	yes	+	+	+	yes	-	-	-	no
B4QBM6	+	+	+	yes	-	+	-	no	-	-	-	no
B4QBN6	+	+	+	yes	+	+	+	yes	-	-	+	no
B4QBQ8	+	+	+	yes	+	+	+	yes	-	-	-	no
B4QBS6	+	+	+	yes	+	+	-	no	-	-	-	no
B4QBV7	+	+	+	yes	+	+	+	yes	+	+	+	yes
B4QBV8	+	+	+	yes	+	+	+	yes	+	+	-	yes
B4QBY1	+	+	+	yes	+	+	+	yes	-	-	-	no
B4QC15	+	+	+	yes	+	+	+	yes	+	+	+	yes
B4QC45;Q3YMT9	+	+	+	yes	+	+	+	yes	+	+	+	yes
B4QC46;B4QT88	+	+	+	yes	+	+	+	yes	+	+	+	yes
B4QC59	-	+	+	no	-	-	+	no	-	-	-	no
B4QC96	+	+	+	yes	+	+	+	yes	+	+	+	yes
B4QCB9	+	+	+	yes	+	-	-	no	-	-	-	no
B4QCE6	+	+	+	yes	-	-	+	no	-	-	-	no
B4QCF3	+	+	+	yes	+	+	+	yes	+	+	+	yes
B4QCF4	+	+	+	yes	+	+	+	yes	+	-	+	yes
B4QCG5	+	+	+	yes	+	+	+	yes	-	+	-	no
B4QCN7	+	+	+	yes	+	+	+	yes	-	-	-	no
B4QCP1	+	+	+	yes	+	-	-	no	-	-	-	no
B4QCY3	+	+	+	yes	-	+	-	no	-	-	-	no
B4QD03	+	+	+	yes	+	+	+	yes	-	-	-	no
B4QD04;B4QD08	+	+	+	yes	+	+	+	yes	-	-	-	no
B4QD05	+	+	+	yes	-	+	-	no	-	-	-	no
B4QD27	+	+	-	no	-	+	-	no	-	-	-	no
B4QD28;B4NVD4	+	+	+	yes	+	+	+	yes	+	+	+	yes
B4QD32	+	+	+	yes	+	+	-	no	-	-	-	no
B4QD42	+	+	+	yes	-	+	-	no	-	-	-	no
B4QD51	+	+	+	yes	+	+	+	yes	+	+	+	yes
B4QD57;B4NSW6;B4NSJ7 ;B4NV18;B4NVK7	+	+	+	yes	+	+	+	yes	+	+	+	yes
B4QD96	+	+	+	yes	-	+	-	no	-	-	-	no
B4QD97	+	+	+	yes	-	-	+	no	-	-	-	no
B4QDC5	+	+	+	yes	+	-	-	no	-	-	-	no
B4QDG8	+	+	+	yes	-	+	-	no	-	-	-	no
B4QDH0	+	+	+	yes	+	+	+	yes	+	+	+	yes
B4QDI1	+	+	+	yes	+	+	+	yes	+	+	+	yes

B4QDI5	+	+	+	yes	+	+	+	yes	-	-	-	no
B4QDI8	+	+	-	no	+	-	-	no	-	-	-	no
B4QDN8	+	+	+	yes	+	+	+	yes	-	-	+	yes
B4QDP1	+	+	+	yes	+	+	+	yes	-	-	-	no
B4QDQ8;B4R795	+	-	+	no	+	-	-	no	-	-	-	no
B4QDR6	+	+	+	yes	-	-	+	no	-	-	-	no
B4QDY3	+	+	+	yes	+	+	+	yes	+	+	+	yes
B4QDZ1	+	+	+	yes	+	+	-	no	-	-	-	no
B4QE23	+	+	+	yes	+	+	+	yes	-	-	-	no
B4QE29	+	+	+	yes	+	+	+	yes	+	+	+	yes
B4QE30	+	+	-	no	+	-	-	no	-	-	-	no
B4QE88	+	+	+	yes	+	-	-	no	-	-	-	no
B4QEA9	+	+	+	yes	+	-	-	no	-	-	-	no
B4QEB0	+	+	+	yes	-	+	-	no	-	-	-	no
B4QEC2	+	+	+	yes	+	+	+	yes	-	-	-	no
B4QEE7	+	+	+	yes	+	+	+	yes	-	-	-	no
B4QEM6	+	+	+	yes	+	-	-	no	-	-	-	no
B4QET2	+	+	+	yes	+	+	+	yes	+	+	+	yes
B4QEU7	+	+	+	yes	+	+	+	yes	+	+	+	yes
B4QEW2	+	+	+	yes	+	+	+	yes	-	-	-	no
B4QEW3	+	+	+	yes	+	+	+	yes	+	+	+	yes
B4QEY1	+	+	+	yes	+	+	+	yes	+	+	+	yes
B4QF23	+	+	+	yes	-	+	-	no	-	-	-	no
B4QF43	+	+	+	yes	+	+	+	yes	+	+	-	yes
B4QF64	-	+	+	no	-	-	+	no	-	-	-	no
B4QF76	+	+	+	yes	-	-	+	no	-	-	-	no
B4QFD2	+	+	+	yes	+	+	+	yes	+	+	+	yes
B4QFD6	+	+	+	yes	+	+	-	no	-	-	-	no
B4QFE2	+	+	+	yes	+	+	+	yes	+	+	+	yes
B4QFE5	+	-	+	no	+	-	-	no	-	-	-	no
B4QFE9	+	+	+	yes	+	+	+	yes	+	+	+	yes
B4QFF3	+	+	+	yes	+	+	+	yes	-	-	-	no
B4QFI9	+	+	+	yes	+	+	+	yes	+	+	+	yes
B4QFK0;B4QSJ3	+	+	+	yes	+	+	+	yes	+	+	+	yes
B4QFL0	+	+	+	yes	+	-	-	no	-	-	-	no
B4QFL9	+	+	+	yes	+	+	+	yes	+	+	-	yes
B4QFV5	+	+	+	yes	-	+	-	no	-	-	-	no
B4QFW4	+	+	+	yes	+	+	+	yes	-	-	+	yes
B4QFY0	+	+	+	yes	+	+	+	yes	+	-	+	yes
B4QFY3	+	+	+	yes	-	+	-	no	-	-	-	no
B4QG12	+	+	+	yes	+	+	-	no	-	-	-	no
B4QG32	+	-	-	no	+	-	-	no	-	-	-	no
B4QG62	+	+	+	yes	+	+	+	yes	-	-	-	no
B4QG64	+	+	+	yes	+	-	-	no	-	-	-	no
B4QGA3	+	+	+	yes	+	+	+	yes	+	+	+	yes
B4QGJ1	+	+	+	yes	+	+	+	yes	-	-	-	no
B4QGJ9	+	+	+	yes	-	+	-	no	-	-	-	no
B4QGN1;Q2XY66	+	+	+	yes	-	+	-	no	-	-	-	no
B4QGR9	+	+	-	no	+	-	-	no	-	-	-	no
B4QGS8	+	+	+	yes	-	+	-	no	-	-	-	no
B4QGZ8	+	+	+	yes	+	+	+	yes	-	-	-	no
B4QH32	+	+	+	yes	-	-	+	no	-	-	-	no
B4QH85	+	+	+	yes	+	-	-	no	-	-	-	no
B4QHA9	+	+	+	yes	+	+	+	yes	+	+	+	yes
B4QHD4	+	+	+	yes	+	+	+	yes	-	-	-	no
B4QHF7	+	+	+	yes	+	-	-	no	-	-	-	no
B4QHG8	+	+	+	yes	-	+	-	no	-	-	-	no
B4QHG9	+	+	+	yes	-	+	-	no	-	-	-	no
B4QHJ0	+	+	+	yes	+	+	+	yes	+	+	+	yes
B4QHJ7	+	+	+	yes	+	+	+	yes	-	+	-	yes
B4QHJ9	+	+	+	yes	+	+	+	yes	+	+	+	yes
B4QHN0	+	+	+	yes	+	+	+	yes	+	+	+	yes
B4QHP5	+	+	+	yes	+	+	+	yes	+	+	+	yes
B4QHS1	+	+	+	yes	+	+	+	yes	+	+	+	yes
B4QHS4	+	-	+	no	+	-	-	no	-	-	-	no
B4QHT8	+	-	-	no	+	-	-	no	-	-	-	no
B4QHU5	+	+	+	yes	-	+	+	no	-	-	-	no
B4QHU8	+	+	+	yes	+	+	+	yes	+	+	+	yes
B4QHV8	+	+	+	yes	+	+	+	yes	-	-	-	no
B4QHX5	+	+	+	yes	-	+	-	no	-	-	-	no
B4QI06	+	+	+	yes	+	+	+	yes	+	+	+	yes
B4QI10	+	+	+	yes	+	+	+	yes	+	+	+	yes
B4QI17	+	+	+	yes	+	+	+	yes	+	+	+	yes
B4QI53	+	+	+	yes	+	+	+	yes	+	+	+	yes
B4QI87	+	+	+	yes	+	+	+	yes	-	-	-	no
B4QIA6	+	+	+	yes	-	-	+	no	-	-	-	no
B4QIE6	+	+	+	yes	+	-	-	no	-	-	-	no
B4QIF5	+	+	+	yes	-	+	-	no	-	-	-	no
B4QIG9	+	+	+	yes	+	+	+	yes	+	+	+	yes
B4QIJ1	+	+	+	yes	+	-	-	no	-	-	-	no
B4QIM2	+	-	+	no	+	-	-	no	-	-	-	no
B4QIN0	+	+	+	yes	+	-	+	no	-	-	-	no
B4QIR2	+	-	-	no	+	-	-	no	-	-	-	no
B4QIS0	+	+	+	yes	+	+	+	yes	+	+	+	yes
B4QIS1	+	+	+	yes	-	+	-	no	-	-	-	no
B4QIY2	+	+	+	yes	+	+	+	yes	+	+	+	yes
B4QIY6	+	+	+	yes	+	+	+	yes	-	-	-	no
B4QIZ0;B4NT34	+	+	+	yes	+	+	+	yes	+	+	+	yes
B4QJ12	+	+	+	yes	+	+	+	yes	-	-	-	no
B4QJ22	+	+	+	yes	+	-	-	no	-	-	-	no
B4QJ36	+	+	+	yes	+	-	-	no	-	-	-	no
B4QJ72	+	+	+	yes	+	+	+	yes	+	+	+	yes
B4QJC5	+	+	+	yes	+	+	+	yes	-	-	-	no
B4QJF3	+	+	+	yes	+	-	-	no	-	-	-	no
B4QJI3;P02826	+	+	+	yes	-	+	-	no	-	-	-	no

B4QJI8	+	+	+	yes	+	+	+	yes	+	+	+	yes
B4QJJ8	+	+	+	yes	+	+	+	yes	+	+	+	yes
B4QJM4	+	+	+	yes	+	+	+	yes	-	-	-	no
B4QJQ6	+	+	-	no	+	-	-	no	-	-	-	no
B4QJS7	+	-	-	no	+	-	-	no	-	-	-	no
B4QJU9	+	+	+	yes	-	+	-	no	-	-	-	no
B4QJV6	+	+	+	yes	+	+	+	yes	+	-	-	no
B4QK02;B4QEM0;Q5R285	+	+	+	yes	+	+	+	yes	+	+	+	yes
B4QK35	+	+	+	yes	-	+	-	no	-	-	-	no
B4QK48	+	+	+	yes	+	-	-	no	-	-	-	no
B4QK59	+	+	+	yes	+	-	-	no	-	-	-	no
B4QKA8	+	+	+	yes	+	+	+	yes	+	+	+	yes
B4QKC8	+	+	+	yes	+	+	+	yes	-	-	-	no
B4QKE0	+	+	+	yes	+	-	-	no	-	-	-	no
B4QKI2;B4R537	+	+	+	yes	+	+	+	yes	-	-	-	no
B4QKJ7	+	+	+	yes	+	+	+	yes	+	+	+	yes
B4QKK6	+	+	+	yes	+	+	+	yes	+	+	+	yes
B4QKN7	+	-	-	no	+	-	-	no	-	-	-	no
B4QKR0	+	+	+	yes	+	+	+	yes	+	+	+	yes
B4QKS0	+	+	+	yes	+	+	+	yes	-	-	+	no
B4QKT7	+	+	+	yes	-	+	-	no	-	-	-	no
B4QKX0	+	+	+	yes	+	+	+	yes	+	+	+	yes
B4QKZ3	+	+	+	yes	+	-	-	no	-	-	-	no
B4QL06	+	+	+	yes	-	+	+	no	-	-	-	no
B4QL41	+	+	+	yes	+	-	-	no	-	-	-	no
B4QL79	+	+	+	yes	-	+	-	no	-	-	-	no
B4QLB5;B4R185	+	+	+	yes	+	+	+	yes	-	-	+	no
B4QLF9	+	+	+	yes	-	+	-	no	-	-	-	no
B4QLI3	+	+	+	yes	+	-	-	no	-	-	-	no
B4QLJ0	+	+	+	yes	+	-	+	no	-	-	-	no
B4QLJ7	+	+	+	yes	-	-	+	no	-	-	-	no
B4QLL8	+	+	+	yes	+	+	+	yes	-	-	-	no
B4QLN4	+	+	+	yes	+	+	+	yes	+	+	+	yes
B4QLU5	+	+	+	yes	+	+	+	yes	-	-	-	no
B4QLV8	+	+	+	yes	+	+	+	yes	+	+	+	yes
B4QLW5	+	+	+	yes	+	+	+	yes	-	-	-	no
B4QM42	+	+	+	yes	-	-	+	no	-	-	-	no
B4QM50	+	+	+	yes	+	+	+	yes	+	+	+	yes
B4QM70;A4ULV7	+	+	+	yes	+	+	+	yes	-	-	-	no
B4QM71	+	+	+	yes	+	+	+	yes	-	+	+	yes
B4QM73	+	+	+	yes	+	+	+	yes	-	-	-	no
B4QMF5	+	+	+	yes	+	+	+	yes	-	-	-	no
B4QMI6	+	+	+	yes	+	+	+	yes	+	+	+	yes
B4QMK8	+	+	+	yes	-	-	+	no	-	-	-	no
B4QMN4	+	+	+	yes	+	+	-	no	-	-	-	no
B4QMN5	+	+	+	yes	+	+	+	yes	-	-	-	no
B4QMQ3	+	+	+	yes	+	-	-	no	-	-	-	no
B4QMT6	+	+	+	yes	+	+	+	yes	-	-	-	no
B4QMU8	+	+	+	yes	+	-	-	no	-	-	-	no
B4QMV1	+	+	+	yes	+	+	+	yes	-	-	-	no
B4QMV2	+	+	+	yes	+	+	+	yes	+	+	+	yes
B4QMV8	+	+	+	yes	+	+	-	no	-	-	-	no
B4QMV9	+	+	+	yes	+	-	-	no	-	-	-	no
B4QMW1	+	+	+	yes	+	+	+	yes	-	+	-	no
B4QMW8	-	+	+	no	-	+	-	no	-	-	-	no
B4QMY7	+	+	+	yes	+	-	+	no	-	-	-	no
B4QMY8	+	+	+	yes	+	-	-	no	-	-	-	no
B4QMY9	+	+	+	yes	+	+	+	yes	+	-	-	no
B4QN01	+	+	+	yes	+	+	-	no	-	-	-	no
B4QN37	+	+	+	yes	-	+	-	no	-	-	-	no
B4QN49	+	+	+	yes	+	+	+	yes	+	-	+	yes
B4QN50	+	+	+	yes	+	+	+	yes	+	+	+	yes
B4QN53	+	+	+	yes	+	+	+	yes	+	+	+	yes
B4QN56	+	+	+	yes	+	+	+	yes	+	+	-	no
B4QN59	+	+	+	yes	-	+	-	no	-	-	-	yes
B4QN63	+	+	+	yes	+	+	+	yes	+	+	+	yes
B4QN74	+	+	+	yes	+	-	+	no	-	-	-	no
B4QN79	+	+	+	yes	+	-	-	no	-	-	-	no
B4QN82;B4NST5	+	+	+	yes	+	+	+	yes	+	+	+	yes
B4QN95;Q1WKX0	+	+	+	yes	+	+	-	no	-	-	-	no
B4QNA6	+	+	+	yes	+	+	+	yes	+	+	+	yes
B4QNA7	+	+	+	yes	+	+	+	yes	-	-	-	no
B4QND2	+	+	+	yes	-	+	-	no	-	-	-	no
B4QNE8;B4QB59	+	+	+	yes	-	+	-	no	-	-	-	no
B4QNF5	+	+	+	yes	-	+	-	no	-	-	-	no
B4QNG9	+	+	+	yes	+	+	-	no	-	-	-	no
B4QNH5	+	+	+	yes	+	-	-	no	-	-	-	no
B4QNJ4	+	+	+	yes	+	+	+	yes	+	+	+	yes
B4QNN6	+	+	+	yes	+	+	+	yes	+	+	+	yes
B4QNU5	+	+	+	yes	-	+	-	no	-	-	-	no
B4QNX7	+	+	+	yes	+	+	+	yes	-	-	-	no
B4QP23	+	+	+	yes	+	+	-	no	-	-	-	no
B4QP26	+	+	+	yes	+	+	+	yes	-	-	-	no
B4QP43;P04810	+	+	+	yes	+	+	+	yes	+	+	+	yes
B4QP55	+	+	+	yes	+	+	+	yes	+	+	+	yes
B4QP86	-	+	+	no	-	+	-	no	-	-	-	no
B4QPB7	+	+	+	yes	+	+	+	yes	+	-	+	yes
B4QPH9	+	+	+	yes	-	+	-	no	-	-	-	no
B4QPJ1	+	+	+	yes	-	+	+	no	-	-	-	no
B4QPN8	+	+	+	yes	+	-	-	no	-	-	-	no
B4QPU5	+	+	+	yes	+	+	+	yes	+	+	+	yes
B4QPW5	+	+	+	yes	+	-	-	no	-	-	-	no
B4QPX4	+	+	+	yes	+	-	-	no	-	-	-	no
B4QPX7	+	+	+	yes	+	+	+	yes	+	+	+	yes
B4QPX8;B4QE73	+	+	+	yes	+	+	+	yes	+	+	+	yes

B4QPY9	+	+	+	yes	+	+	+	yes	-	+	-	no
B4QPZ8	+	+	+	yes	-	+	-	no	-	-	-	no
B4QQ01	+	+	+	yes	+	+	+	yes	-	-	-	no
B4QQ07	+	+	+	yes	-	+	-	no	-	-	-	no
B4QQ43	+	+	+	yes	+	+	+	yes	+	+	+	yes
B4QQ85	+	+	+	yes	+	+	+	yes	+	+	+	yes
B4QQD8	+	+	+	yes	+	+	+	yes	+	-	-	no
B4QQN5	+	+	+	yes	+	+	+	yes	+	+	+	yes
B4QR27	+	+	-	no	-	+	-	no	-	-	-	no
B4QR52	+	+	+	yes	+	+	+	yes	+	+	+	yes
B4QR56	+	-	+	no	-	-	+	no	-	-	-	no
B4QR61	+	+	+	yes	-	+	+	no	-	-	-	no
B4QR64	+	+	+	yes	+	+	+	yes	+	+	+	yes
B4QR91	+	+	-	no	-	+	-	no	-	-	-	no
B4QRB4	+	+	+	yes	-	+	+	no	-	-	-	no
B4QRD0	+	+	+	yes	+	+	+	yes	+	+	+	yes
B4QRD5	+	+	+	yes	-	+	-	no	-	-	-	no
B4QRE7	+	+	+	yes	+	+	+	yes	+	+	+	yes
B4QRE8	+	+	+	yes	-	+	-	no	-	-	-	no
B4QRI0	+	+	+	yes	-	+	-	no	-	-	-	no
B4QRJ7	+	+	+	yes	+	+	+	yes	+	-	+	yes
B4QRJ8	+	+	+	yes	+	+	+	yes	-	-	-	no
B4QRL3	+	+	+	yes	-	+	-	no	-	-	-	no
B4QRN6	+	+	+	yes	+	+	+	yes	+	+	+	yes
B4QRN9	+	+	+	yes	+	-	+	no	-	-	-	no
B4QRQ5	+	+	+	yes	-	-	+	no	-	-	-	no
B4QRV4	+	+	+	yes	-	+	-	no	-	-	-	no
B4QRW6;B6UXN7;B6UXN8	+	+	+	yes	+	+	+	yes	-	-	-	no
B4QRX2	+	+	+	yes	-	+	-	no	-	-	-	no
B4QS14	+	+	+	yes	+	-	-	no	-	-	-	no
B4QS21	+	+	+	yes	-	+	-	no	-	-	-	no
B4QS70	+	+	+	yes	+	-	-	no	-	-	-	no
B4QS73	+	+	+	yes	-	-	+	no	-	-	-	no
B4QS89	+	+	+	yes	+	+	+	yes	+	+	+	yes
B4QSA7	+	+	+	yes	+	-	-	no	-	-	-	no
B4QSA8;A0ANW4	+	+	+	yes	+	+	+	yes	-	-	-	no
B4QSA9	+	+	+	yes	+	+	+	yes	-	-	-	no
B4QSB3;B4F4Y1;B4QT73;B4NVV6	+	+	+	yes	+	-	+	no	-	-	-	no
B4QSD0	+	+	+	yes	+	+	+	yes	+	+	+	yes
B4QSD4	+	+	+	yes	+	+	+	yes	+	+	+	yes
B4QSD6;Q9NGJ5	+	+	+	yes	+	+	+	yes	-	-	-	no
B4QSD7	+	+	+	yes	+	-	-	no	-	-	-	no
B4QSI7	+	+	+	yes	-	+	-	no	-	-	-	no
B4QST7	+	+	+	yes	-	+	-	no	-	-	-	no
B4QSV7	+	+	+	yes	+	-	-	no	-	-	-	no
B4QSX3	+	+	+	yes	+	+	+	yes	-	-	-	no
B4QSX7	+	+	+	yes	+	-	-	no	-	-	-	no
B4QT28	-	+	+	no	-	+	-	no	-	-	-	no
B4QT31	+	+	+	yes	+	-	-	no	-	-	-	no
B4QT92	+	+	+	yes	+	+	+	yes	+	+	+	yes
B4QT94	+	+	+	yes	-	+	-	no	-	-	-	no
B4QTB5	+	+	+	yes	+	+	+	yes	+	+	+	yes
B4QTB6	+	+	+	yes	+	+	+	yes	-	-	-	no
B4QTB7	+	+	+	yes	+	+	+	yes	-	-	-	no
B4QTF8	+	+	+	yes	+	-	-	no	-	-	-	no
B4QTH4;Q2XYL0;Q2XYL1	+	+	+	yes	+	+	+	yes	+	+	+	yes
B4QTI4	+	+	-	no	+	-	-	no	-	-	-	no
B4QTK7	+	+	-	no	+	-	-	no	-	-	-	no
B4QTN3	+	+	+	yes	+	-	-	no	-	-	-	no
B4QTV0	+	+	+	yes	+	+	+	yes	-	-	-	no
B4QU25	+	+	+	yes	+	+	+	yes	+	-	-	no
B4QU42	+	+	+	yes	-	-	+	no	-	-	-	no
B4QU44	+	+	+	yes	+	-	-	no	-	-	-	no
B4QU54;U6SXS5;Q73HL2;C0R2M4	+	+	+	yes	+	+	+	yes	-	-	-	no
B4QU67	+	+	+	yes	+	+	+	yes	+	+	+	yes
B4QU71	+	+	+	yes	+	+	+	yes	+	+	+	yes
B4QU96	+	+	-	no	-	+	-	no	-	-	-	no
B4QUB9	+	+	+	yes	+	+	+	yes	+	+	-	yes
B4QUF0	+	+	+	yes	+	+	+	yes	+	+	+	yes
B4QUF1	+	+	+	yes	+	+	+	yes	-	-	-	no
B4QUG7	+	+	+	yes	-	+	+	no	-	-	-	no
B4QUH7	+	+	+	yes	+	+	+	yes	+	+	+	yes
B4QUH9	+	+	+	yes	+	-	-	no	-	-	-	no
B4QUJ7;B4NTK7;B4NW17	+	+	+	yes	+	+	+	yes	+	+	+	yes
B4QUK7	+	-	+	no	+	-	-	no	-	-	-	no
B4QUM6	+	+	+	yes	+	+	+	yes	+	+	+	yes
B4QUP5	+	+	+	yes	+	+	-	no	-	-	-	no
B4QUP7	+	+	+	yes	-	+	-	no	-	-	-	no
B4QUR4	+	+	+	yes	+	+	+	yes	+	+	+	yes
B4QUR6	+	+	+	yes	+	+	+	yes	-	-	-	no
B4QUS0	+	+	+	yes	+	+	+	yes	-	-	-	no
B4QUX1	+	+	-	no	+	-	-	no	-	-	-	no
B4QV16	+	+	+	yes	+	+	+	yes	-	+	+	no
B4QV50	+	+	+	yes	+	+	+	yes	+	+	+	yes
B4QV53	+	+	+	yes	+	+	+	yes	+	-	-	no
B4QV81;B4R0Q1	+	+	+	yes	+	+	-	no	-	-	-	no
B4QV95	+	+	+	yes	-	+	+	no	-	-	-	no
B4QVB0	+	+	+	yes	+	-	+	no	-	-	-	no
B4QVD4	+	+	+	yes	+	-	+	no	-	-	-	no
B4QVD5	+	+	+	yes	+	+	+	yes	+	+	+	yes
B4QVH6	+	+	+	yes	+	+	+	yes	+	+	+	yes
B4QVI2	+	+	+	yes	+	+	+	yes	+	+	+	yes
B4QVJ0	+	-	-	no	+	-	-	no	-	-	-	no

B4QVK9	+	+	+	yes	+	+	+	yes	+	+	+	yes
B4QVL3	+	+	+	yes	+	+	+	yes	+	+	+	yes
B4QVP4	+	+	+	yes	+	+	+	yes	+	+	+	yes
B4QVU1	+	+	+	yes	+	+	+	yes	-	-	-	no
B4QVY0	+	+	+	yes	+	+	+	yes	+	+	-	yes
B4QVY6	+	+	+	yes	+	+	-	no	-	-	-	no
B4QVZ0	+	+	+	yes	-	+	-	no	-	-	-	no
B4QW23	+	+	+	yes	-	+	-	no	-	-	-	no
B4QW40;A5XD00;A5XCZ4	+	+	+	yes	+	+	+	yes	+	+	+	yes
B4QWA7	+	+	+	yes	+	+	+	yes	+	-	+	yes
B4QWB7	+	+	+	yes	+	+	+	yes	-	-	-	no
B4QWD1	+	+	+	yes	-	-	+	no	-	-	-	no
B4QWD5	+	+	+	yes	+	+	+	yes	-	-	-	no
B4QWG0	+	+	+	yes	+	+	+	yes	+	+	+	yes
B4QWH9	+	+	+	yes	-	+	-	no	-	-	-	no
B4QWI2	+	+	+	yes	+	+	+	yes	-	+	+	yes
B4QWI8	+	+	+	yes	+	+	+	yes	-	-	-	no
B4QWI9	+	+	+	yes	+	+	+	yes	-	+	-	no
B4QWJ9	+	+	+	yes	+	-	-	no	-	-	-	no
B4QWL8	-	+	+	no	-	-	+	no	-	-	-	no
B4QWM7	+	+	+	yes	-	-	+	no	-	-	-	no
B4QWN3;B4QW27	+	+	+	yes	+	+	+	yes	+	+	+	yes
B4QWS7;B4NVS1;B4NV60;B4Q4E2	+	+	+	yes	+	+	+	yes	+	+	+	yes
B4QWU4	+	+	+	yes	+	+	+	yes	-	-	-	no
B4QWV7	+	+	+	yes	+	+	-	no	-	-	-	no
B4QWV8	+	+	+	yes	-	+	-	no	-	-	-	no
B4QWX8	+	+	+	yes	-	+	-	no	-	-	-	no
B4QWY3	+	+	+	yes	+	+	+	yes	+	+	+	yes
B4QWY4	+	+	+	yes	-	-	+	no	-	-	-	no
B4QWY9	+	+	+	yes	+	+	+	yes	+	+	+	yes
B4QWZ3;B4QWZ5;B4QWZ4	+	+	+	yes	+	-	-	no	-	-	-	no
B4QWZ9	+	-	-	no	+	-	-	no	-	-	-	no
B4QX60	+	+	+	yes	-	+	-	no	-	-	-	no
B4QX63	+	+	+	yes	+	-	+	no	-	-	-	no
B4QXB7	+	-	+	no	+	-	-	no	-	-	-	no
B4QXC7	+	+	+	yes	+	+	+	yes	-	-	-	no
B4QXK2	+	+	+	yes	+	+	+	yes	-	-	-	no
B4QXM7	+	+	+	yes	+	-	-	no	-	-	-	no
B4QXQ9	+	-	+	no	+	-	-	no	-	-	-	no
B4QXR2	+	+	+	yes	+	+	+	yes	+	+	+	yes
B4QXU2	+	+	-	no	-	+	-	no	-	-	-	no
B4QXX1	+	+	+	yes	+	+	+	yes	+	+	+	yes
B4QXY2	+	+	+	yes	+	+	+	yes	-	-	-	no
B4QXZ8	+	+	+	yes	+	-	-	no	-	-	-	no
B4QXZ9;B4QPF3	+	+	+	yes	+	+	+	yes	+	+	+	yes
B4QY02	+	+	+	yes	+	+	+	yes	+	+	+	yes
B4QY05	+	+	-	no	+	-	-	no	-	-	-	no
B4QY28	+	+	+	yes	+	-	+	no	-	-	-	no
B4QY54	+	+	+	yes	+	+	+	yes	+	+	+	yes
B4QY60	+	+	+	yes	+	+	+	yes	+	+	+	yes
B4QY79	+	+	+	yes	-	-	+	no	-	-	-	no
B4QYA4	+	+	+	yes	+	+	+	yes	-	-	-	no
B4QYD7;Q3YMT3	+	+	+	yes	+	+	+	yes	-	-	-	no
B4QYH1	+	+	+	yes	+	-	-	no	-	-	-	no
B4QYI3	+	+	+	yes	+	+	-	no	-	-	-	no
B4QYJ3	+	+	+	yes	+	+	+	yes	+	+	+	yes
B4QYK2	+	+	+	yes	+	+	+	yes	+	+	-	no
B4QYL5;Q6BCY9	+	+	+	yes	+	+	+	yes	+	+	+	yes
B4QYL9	+	+	+	yes	+	+	+	yes	-	-	-	no
B4QYN5	+	+	+	yes	+	+	+	yes	+	-	-	no
B4QYQ4	+	+	+	yes	-	+	-	no	-	-	-	no
B4QYR3	+	+	+	yes	+	+	+	yes	-	-	-	no
B4QYR5	+	+	+	yes	-	-	+	no	-	-	-	no
B4QYZ5	+	+	+	yes	-	-	+	no	-	-	-	no
B4QZ26	+	+	+	yes	+	+	+	yes	+	+	+	yes
B4QZ28	+	+	+	yes	+	+	+	yes	+	+	+	yes
B4QZ33;Q9NGH4;Q9NGH3;Q9NGH2;B4QSF6	+	+	+	yes	+	+	+	yes	+	+	+	yes
B4QZ36	+	-	+	no	+	-	-	no	-	-	-	no
B4QZ38	+	+	+	yes	-	+	-	no	-	-	-	no
B4QZ72	+	+	+	yes	+	+	+	yes	+	+	+	yes
B4QZB3	+	+	+	yes	+	+	+	yes	+	+	+	yes
B4QZC5	+	+	+	yes	+	-	-	no	-	-	-	no
B4QZC9	+	-	-	no	+	-	-	no	-	-	-	no
B4QZD1	+	+	+	yes	-	+	-	no	-	-	-	no
B4QZG5	+	+	+	yes	-	+	-	no	-	-	-	no
B4QZH5	+	-	+	no	+	-	-	no	-	-	-	no
B4QZH7	+	+	+	yes	+	+	+	yes	-	-	-	no
B4QZI7	+	+	+	yes	+	+	+	yes	+	+	+	yes
B4QZI8	+	+	+	yes	+	+	+	yes	-	-	-	no
B4QZP1;B4QYZ8;K7XHV7;K7XHV1;K7X506;K7WS60;K7WS55;K7WQ13;K7WQ10;K7WQ04;K7WQ02;K7WKP8;K7WKN8;K7WKN4;K7WKP4;K7X518;K7X515;K7WS66;K7XHV5;K7X501;K7WS62;B4QVZ6	+	+	+	yes	+	+	+	yes	+	+	+	yes
B4QZS0	+	+	+	yes	-	+	-	no	-	-	-	no
B4QZS5;A0APB5	+	+	+	yes	-	-	+	no	-	-	-	no
B4QZZ3	+	+	+	yes	+	+	+	yes	-	-	-	no
B4QZZ9	+	+	+	yes	+	+	+	yes	+	+	+	yes
B4R013	+	+	+	yes	+	+	+	yes	-	-	-	no
B4R021	+	+	+	yes	+	+	+	yes	-	+	+	yes
B4R028	+	+	+	yes	+	+	+	yes	-	-	-	no

B4R030;Q9N6M6;Q9NGA6	+	+	+	yes	+	+	+	yes	+	+	+	yes
B4R062	+	+	+	yes	-	-	+	no	-	-	-	no
B4R084	+	+	+	yes	+	+	+	yes	+	+	+	yes
B4R0B0	+	+	+	yes	+	-	-	no	-	-	-	no
B4R0J4	+	+	+	yes	+	+	+	yes	-	-	-	no
B4R0K9	+	+	+	yes	+	+	+	yes	+	+	+	yes
B4R0L0	+	+	+	yes	+	+	-	no	-	-	-	no
B4R0M6	+	+	+	yes	-	+	-	no	-	-	-	no
B4R0M8	+	+	+	yes	-	+	-	no	-	-	-	no
B4R0S9	+	+	+	yes	+	+	+	yes	+	+	+	yes
B4R0U6	+	+	+	yes	+	+	+	yes	+	+	+	yes
B4R0X8	+	+	+	yes	+	+	+	yes	+	+	-	yes
B4R0X9	+	+	+	yes	+	+	+	yes	+	+	+	yes
B4R0Y1	+	+	+	yes	+	+	+	yes	+	-	+	yes
B4R123;B4QRC2	+	+	+	yes	+	+	+	yes	+	+	-	yes
B4R145	+	+	+	yes	+	+	+	yes	-	-	-	no
B4R153	+	+	+	yes	+	+	+	yes	-	-	-	no
B4R154	+	+	+	yes	+	+	+	yes	-	+	+	yes
B4R169	+	+	+	yes	+	-	-	no	-	-	-	no
B4R170	+	+	+	yes	+	-	+	no	-	-	-	no
B4R173	+	+	+	yes	+	+	+	yes	+	+	+	yes
B4R174	+	+	+	yes	+	+	+	yes	+	+	+	yes
B4R189;P83968;B4QKQ2; B4QIF6	-	+	+	no	-	+	-	no	-	-	-	no
B4R194	+	+	+	yes	+	-	+	no	-	-	-	no
B4R199	+	+	+	yes	+	+	+	yes	-	-	+	no
B4R1B2	+	+	+	yes	+	-	-	no	-	-	-	no
B4R1G1	+	+	+	yes	+	+	+	yes	+	+	-	no
B4R1G2	+	+	+	yes	-	+	-	no	-	-	-	no
B4R1G3	+	+	+	yes	+	+	+	yes	+	+	+	yes
B4R1J4	+	+	+	yes	+	+	+	yes	-	-	-	no
B4R1K1	+	+	+	yes	+	+	+	yes	+	+	+	yes
B4R1L1	+	+	+	yes	+	+	+	yes	-	-	-	no
B4R1L2	+	+	+	yes	+	+	+	yes	+	-	-	yes
B4R1M4	+	+	+	yes	-	+	-	no	-	-	-	no
B4R1P5;B4QUZ5	+	+	+	yes	+	+	+	yes	+	+	+	yes
B4R1Q9	+	+	+	yes	+	+	+	yes	-	-	-	no
B4R1R4	+	+	+	yes	+	+	+	yes	-	-	-	no
B4R1R7	+	+	+	yes	+	+	+	yes	+	+	+	yes
B4R1S2	+	+	+	yes	-	+	-	no	-	-	-	no
B4R1W6	+	+	+	yes	+	-	-	no	-	-	-	no
B4R1W9	-	+	+	no	-	+	-	no	-	-	-	no
B4R1X7	+	+	+	yes	+	-	-	no	-	-	-	no
B4R1Z4	+	+	+	yes	+	+	+	yes	-	-	+	no
B4R222;B4QT07	+	+	+	yes	-	+	+	no	-	-	-	no
B4R239	+	+	+	yes	+	+	+	yes	+	+	-	yes
B4R242	+	+	+	yes	+	+	+	yes	+	-	+	yes
B4R2A1	+	+	+	yes	+	-	+	no	-	-	-	no
B4R2I2;Q6W4K6;U6SYW6; Q73IG3;C0R5I6	+	+	+	yes	+	+	+	yes	+	+	+	yes
B4R2I4	+	+	+	yes	+	+	+	yes	+	+	+	yes
B4R2I5	+	+	+	yes	+	+	+	yes	+	+	+	yes
B4R2K4	+	+	+	yes	-	+	-	no	-	-	-	no
B4R2R2;B4Q411	+	+	+	yes	+	+	+	yes	+	+	+	yes
B4R2Y3	+	+	+	yes	+	+	+	yes	-	+	-	no
B4R2Z8	+	+	+	yes	+	+	+	yes	-	-	-	no
B4R345	+	-	+	no	+	-	-	no	-	-	-	no
B4R356	+	+	+	yes	-	+	-	no	-	-	-	no
B4R365	+	+	+	yes	+	-	+	no	-	-	-	no
B4R380;F6J5U6;F6JM47	+	+	+	yes	+	+	+	yes	+	+	+	yes
B4R3C5	+	+	+	yes	-	+	+	no	-	-	-	no
B4R3C8;Q6WAS1;B4Q8G5	+	+	+	yes	-	+	-	no	-	-	-	no
B4R3D0	+	+	+	yes	+	-	+	no	-	-	-	no
B4R3F1	+	+	-	no	+	-	-	no	-	-	-	no
B4R3H2	+	+	+	yes	+	-	-	no	-	-	-	no
B4R3H4	+	+	+	yes	+	+	+	yes	-	-	-	no
B4R3I9	+	+	+	yes	+	+	+	yes	+	+	+	yes
B4R3Q8;B4R279	+	+	+	yes	+	+	+	yes	-	-	+	no
B4R3R1	+	+	+	yes	+	-	-	no	-	-	-	no
B4R3R2	+	+	+	yes	+	-	-	no	-	-	-	no
B4R3Z3;B4Q3U0	+	+	+	yes	+	-	-	no	-	-	-	no
B4R407	+	+	+	yes	-	-	+	no	-	-	-	no
B4R472;Q86DS5;Q86DS4	+	+	+	yes	+	+	+	yes	+	+	+	yes
B4R488;A9YI34	+	+	+	yes	+	+	+	yes	+	+	+	yes
B4R496	+	+	+	yes	+	+	+	yes	+	+	+	yes
B4R497	+	+	+	yes	+	-	+	no	-	-	-	no
B4R4A2	+	+	+	yes	+	+	-	no	-	-	-	no
B4R4B3;F6J6A5	+	+	+	yes	+	+	+	yes	+	+	+	yes
B4R4B5	+	+	+	yes	+	+	+	yes	+	+	+	yes
B4R4B9;B4NTK2	+	+	+	yes	+	-	-	no	-	-	-	no
B4R4D8	+	+	+	yes	+	+	+	yes	-	-	-	no
B4R4F7	+	+	+	yes	-	+	-	no	-	-	-	no
B4R4F9	+	+	+	yes	+	-	-	no	-	-	-	no
B4R4H1	+	+	+	yes	+	-	-	no	-	-	-	no
B4R4I4	+	+	+	yes	+	+	+	yes	-	-	-	no
B4R4J0	+	+	+	yes	-	-	+	no	-	-	-	no
B4R4N1	+	+	+	yes	+	-	-	no	-	-	-	no
B4R4P7;F6JIP4;F6JIP8	+	+	+	yes	+	+	+	yes	+	-	-	no
B4R4Y2	+	+	+	yes	+	+	+	yes	+	+	+	yes
B4R4Z9	+	+	+	yes	+	+	+	yes	+	-	+	yes
B4R508	+	+	+	yes	-	+	-	no	-	-	-	no
B4R576	+	+	+	yes	+	+	+	yes	+	+	+	yes
B4R5A1;B4QCQ5	+	+	+	yes	+	+	+	yes	+	+	+	yes
B4R5A4	+	+	+	yes	+	+	+	yes	-	+	-	no

B4R5B8	+	+	+	yes	-	-	+	no	-	-	-	no
B4R5E7;B4NRW4;B4Q994;B4QPZ7;B4QPZ6	+	+	+	yes	+	+	+	yes	+	+	+	yes
B4R5G2	+	+	+	yes	+	+	+	yes	-	-	-	no
B4R5H2	+	+	+	yes	+	+	+	yes	-	-	-	no
B4R5H3	+	-	+	no	+	-	-	no	-	-	-	no
B4R5I8	+	+	+	yes	+	+	-	no	-	-	-	no
B4R5J2	+	+	+	yes	+	-	-	no	-	-	-	no
B4R5K8	+	+	-	no	-	+	-	no	-	-	-	no
B4R5M2	+	+	+	yes	+	+	+	yes	+	+	+	yes
B4R5N1	+	+	+	yes	+	+	+	yes	-	-	-	no
B4R5R9	+	+	+	yes	-	+	-	no	-	-	-	no
B4R5S2	+	+	+	yes	-	+	-	no	-	-	-	no
B4R5U1	+	+	+	yes	+	+	+	yes	+	+	+	yes
B4R5U3	+	+	+	yes	-	+	-	no	-	-	-	no
B4R5U9	+	+	-	no	+	-	-	no	-	-	-	no
B4R5V4	+	+	+	yes	+	+	+	yes	+	+	+	yes
B4R5V5	+	+	+	yes	+	+	-	no	-	-	-	no
B4R5W4	+	+	+	yes	-	-	+	no	-	-	-	no
B4R614	+	+	+	yes	+	+	+	yes	-	-	-	no
B4R649	+	+	+	yes	-	+	-	no	-	-	-	no
B4R670;F6J911	+	+	+	yes	+	-	-	no	-	-	-	no
B4R671;B4QD81	+	+	+	yes	+	-	-	no	-	-	-	no
B4R680	+	+	+	yes	+	+	+	yes	+	+	+	yes
B4R691	+	+	+	yes	-	-	+	no	-	-	-	no
B4R6B4	+	+	+	yes	-	-	+	no	-	-	-	no
B4R6D1;B4QZ66	+	+	+	yes	+	+	+	yes	-	-	-	no
B4R6F7	+	+	+	yes	+	-	-	no	-	-	-	no
B4R6J1	+	+	+	yes	+	-	-	no	-	-	-	no
B4R6J4;A4ULY1;A8E012	+	+	+	yes	+	+	+	yes	+	-	-	no
B4R6M1	+	+	+	yes	-	+	-	no	-	-	-	no
B4R6S1	+	+	+	yes	+	+	+	yes	+	+	+	yes
B4R6U5	+	+	+	yes	+	+	+	yes	-	-	-	no
B4R6X0	+	+	+	yes	+	+	+	yes	+	+	+	yes
B4R6Y8	+	+	+	yes	+	-	-	no	-	-	-	no
B4R707	+	+	+	yes	-	+	-	no	-	-	-	no
B4R752	+	+	+	yes	+	+	+	yes	+	-	+	yes
B4R757	+	+	+	yes	+	+	+	yes	+	+	+	yes
B4R7E7	+	+	+	yes	+	+	+	yes	+	+	+	yes
B4R7G1	+	+	+	yes	+	+	+	yes	+	+	+	yes
B4R7I4	+	+	+	yes	+	+	+	yes	+	+	+	yes
B4R7I5	+	+	+	yes	+	+	+	yes	+	+	+	yes
B4R7M3;A9YKX4;F6JLD5;F6JLB4	+	+	+	yes	+	+	+	yes	+	-	-	no
B4R7M9;B4R7M8	+	+	+	yes	+	+	+	yes	+	+	+	yes
B4R7Q4	+	+	+	yes	-	+	-	no	-	-	-	no
B4R7Q8	+	+	+	yes	+	+	+	yes	+	+	+	yes
B4R7R9	+	+	+	yes	+	+	+	yes	+	+	+	yes
B4R7T3;Q7YSQ6;A2TE06	+	+	+	yes	-	+	-	no	-	-	-	no
B8Q5E0;P61852;B8Q5D9;B8Q5D8	+	+	+	yes	+	+	+	yes	-	+	+	yes
C0R3A1;Q73GZ8;U6SX48	+	+	+	yes	-	+	-	no	-	-	-	no
C0R3L0	+	+	-	no	-	+	-	no	-	-	-	no
C0R4H0	+	+	-	no	-	+	-	no	-	-	-	no
F6J7A2;B4NVC9	+	+	+	yes	+	+	+	yes	+	+	+	yes
F6J835	+	+	+	yes	-	+	+	no	-	-	-	no
F6J856	+	+	+	yes	-	-	+	no	-	-	-	no
F6JHH8;F6JHH7;F6JHH6;A9YHD1;B5LFV8;F6JHH0;B5LFW6	+	+	+	yes	-	+	-	no	-	-	-	no
F6JNM1;F6JNJ8;F6JJ07;F6JIZ3;F6JIY6;F6JIY5;A9YJ05	+	+	-	no	-	+	-	no	-	-	-	no
L7X3S7;H9EAT8;C8CB15	+	+	+	yes	-	+	-	no	-	-	-	no
O52076;Q09TN6;Q7BVI0;O52077;B0ZSE1;B7TX60;B7TX59;U6SUJ5;Q73G97;A0EZP4;Q5EFK7;Q5EFK9;Q5EFK8	+	+	+	yes	+	+	+	yes	-	-	+	no
P41573;B4R3E6;Q9N6B3	+	+	+	yes	+	+	-	no	-	-	-	no
P50253;H9EAV1	+	+	+	yes	+	+	+	yes	+	-	-	no
P52030	+	+	+	yes	+	-	-	no	-	-	-	no
P59782;B4NVQ8;B4NV27	+	+	+	yes	+	+	+	yes	-	+	+	no
P61128;G8E4T2	+	+	+	yes	+	+	+	yes	+	+	+	yes
P67805	+	+	+	yes	+	+	+	yes	+	+	+	yes
P84043;B4NVU5;B4Q4E3	+	+	+	yes	+	+	+	yes	+	+	+	yes
Q0H6L1;B4R2Z5	+	+	+	yes	+	+	+	yes	+	+	+	yes
Q1WKW0;Q8MTT3;Q8MTT2;Q8MTT1;Q8MM95	+	+	+	yes	+	+	+	yes	-	-	-	no
Q24641;A0A077HBL2;C7FGZ3	+	+	+	yes	+	+	+	yes	+	+	+	yes
Q24654	+	+	+	yes	+	+	+	yes	-	-	-	no
Q27IR9;Q27IR8;B4Q4T0	+	+	-	no	+	-	-	no	-	-	-	no
Q2QBI9;B4QS66	+	+	+	yes	-	-	+	no	-	-	-	no
Q2XYB5;Q71TY0	+	-	+	no	+	-	-	no	-	-	-	no
Q2XYG9;B2XZV3;B4QMM8;B2XZV5	+	+	+	yes	+	+	+	yes	+	+	+	yes
Q3YMT5	+	+	+	yes	+	+	+	yes	-	-	-	no
Q3YMU1;B4Q596	+	+	+	yes	+	+	+	yes	+	+	+	yes
Q3YMV5;B4Q452	+	+	+	yes	+	+	+	yes	-	-	-	no
Q49BM3;B6UVR2;B6UVR1;B6UVQ8	+	+	+	yes	-	+	-	no	-	-	-	no

Q58SJ6;Q58SJ0;Q58SI9;Q58SK1;Q58SK0;Q58SJ4;Q58SI4;Q58SI3;Q58SL1;Q58SL0;Q58SK8;Q58SK7;Q58SK6;Q58SK4;Q58SK3;Q58SK2;Q58SJ7;Q58SJ5;Q58SJ3;Q58SI8;Q58SI5;Q58SI1;Q6V6N6;Q6V6N4;Q6V6N2;Q58SJ9;Q58SJ2;Q58SI2;Q58SJ1;Q58SI7;Q58SI6	+	+	+	yes	-	+	+	no	-	-	-	no
Q5R288	+	+	+	yes	+	+	+	yes	+	+	+	yes
Q5R290;Q868J7	+	+	+	yes	+	+	+	yes	+	-	-	no
Q5R297;B4QEK6	+	+	+	yes	+	-	+	no	-	-	-	no
Q6J8J6;Q8T6S5;Q6J8J9;Q6J8J8;Q6J8J1	+	+	+	yes	+	+	+	yes	+	+	+	yes
Q6QH37	+	+	+	yes	+	+	+	yes	+	+	-	yes
Q73HS8;U6SYI9	-	-	+	no	-	-	+	no	-	-	-	no
Q73IX6;Q73H85;C0R544;C0R311;U6SZG4;U6SW27;U6SWZ6	+	+	+	yes	-	+	-	no	-	-	-	no
Q7JNS1;B4R1I7	+	+	+	yes	+	+	+	yes	+	+	+	yes
Q7YST2	+	+	+	yes	+	+	+	yes	-	-	-	no
Q7YZE8;Q7YZE7;A2TDX1;A2TDW5	+	+	+	yes	+	+	+	yes	-	-	-	no
Q7YZE9;Q6W758	+	+	+	yes	+	+	+	yes	-	-	-	no
Q8I1N2;B4NVR7;B4R3B4;B4NVE1;B4Q4E4;B4NVU6;B4NVQ2	+	+	+	yes	+	+	+	yes	+	+	-	yes
Q9GSU4;Q9GSU3;Q9GSU2;Q9GSU0;B4QS96;Q9GSV1;Q9GSV0;Q9GSU8;B4QTJ5;B4QS95;Q6UNZ7;Q9GSU1;Q9GSU7;Q9GSU9	+	+	+	yes	+	+	+	yes	+	+	+	yes
Q9MDZ9;H9EAW0;H9EAU7	+	+	+	yes	-	+	-	no	-	-	-	no
Q9N618;F6JJ14;B4R5D9;F6J1B9	+	+	+	yes	+	-	-	no	-	-	-	no
Q9N6D2;B4Q844;B4R6E4;B4QRX5	+	+	+	yes	+	-	-	no	-	-	-	no
Q9N6E0;Q9NGD2	+	+	+	yes	+	+	+	yes	+	+	+	yes
Q9NGE4;Q9NGE0	+	+	+	yes	+	-	-	no	-	-	-	no
Q9NGJ2;Q9NGJ0;Q9N6R2;Q9NGJ1;Q9N6E6;B4R683	+	+	+	yes	+	+	+	yes	+	+	+	yes
Q9RBN2;B0ZSE2;O52082	+	+	+	yes	-	+	-	no	-	+	-	no
U6SV82;Q73H30;C0R3I9	+	+	+	yes	-	-	+	no	-	-	-	no
U6SW00;Q73I43	-	+	-	no	-	+	-	no	-	-	-	no
U6SW43;Q73HB2;C0R2Y2	+	+	+	yes	+	+	+	yes	+	+	+	yes
U6SW99;Q73I70;C0R2V4	-	+	+	no	-	+	-	no	-	-	-	no
U6SWA5;Q73HC4	+	+	+	yes	-	+	-	no	-	-	-	no
U6SWV5;Q73IX0;C0R550	+	+	+	yes	-	-	+	no	-	-	-	no
U6SX25;Q73I71;C0R2V3;Q2MZ27	+	+	+	yes	+	+	+	yes	-	-	+	no
U6SXX3;Q73H52	-	+	+	no	-	-	+	no	-	-	+	no

Table S3

Functional Classification	Protein name	UniProt IDs	P-value	Average LFQ intensity values			Relative abundance		
				Dmel Uninf	Dmel *w*Mel	Dmel *w*MelCS	wMel/Uninf	*w*MelCS/Uninf	*w*Mel/*w*MelCS
Amino acid transport and metabolism	Eip55E	Q7JXZ2;O97121	0.043	2541	3229	4031	1.271	1.587	1.248
Carbohydrate transport and metabolism	Aldolase	P07764;C8VV14;F3YDE2;F3YDB5;A4V3G1;F3YDA0;Q86NZ4	0.009	13457	12373	8480	0.919	0.630	0.685
	Glycogen phosphorylase	Q9XTL9;A4UZZ4	0.003	11608	9744	16801	0.839	1.447	1.724
	Succinyl coenzyme A synthetase α subunit	Q94522;M9MRQ9	0.047	n/a	4819	2846	n/a	n/a	0.591
Chromatin structure and dynamics	Decondensation factor 31	O16043	0.032	24175	22073	31509	0.913	1.303	1.427
	Protein on ecdysone puffs	M9NG39;P41073;D5AEL0;C0PTV1	0.012	2573	3669	n/a	1.426	n/a	n/a
	Vig2	Q960D3;Q9VBX3;B5RIY3;Q6NLK4	0.038	5086	4414	6766	0.868	1.330	1.533
Cytoskeleton and cell motility	Ciboulot	O97428;D5AEL7;Q8IRS7	0.013	18055	15487	24910	0.858	1.380	1.608
Detoxification	Glutathione S-transferase D1	P20432	0.007	10799	11103	17559	1.028	1.626	1.581
	Peroxinectin-like	Q9VEG6	0.032	7373	6556	12565	0.889	1.704	1.917
	Transferrin 1	Q9VWV6;O97355;A9UNH0;F6J873;F6J859;F6J843;F6J836;F6J874;F6J842;F6J831;F6J817	0.029	15457	30916	n/a	2.000	n/a	n/a
Energy production and conversion	ATP synthase	Q9VKM3	0.043	3495	5197	3628	1.487	1.038	0.698
	ATP synthase, δ subunit	Q9W2X6	0.000	6258	5554	8553	0.888	1.367	1.540
	Isocitrate dehydrogenase	Q7KUB0;Q8IQA7;Q9VSI6;Q7KUB1;B7Z0E0;C8VV61	0.030	7062	5481	9280	0.776	1.314	1.693
Intracellular trafficking, secretion, and vesicular transport	Pendulin	P52295	0.023	9405	9127	12626	0.970	1.342	1.383
	Trailer hitch	M9PF14;M9PF20;M9PFF9;M9PFG3	0.031	33664	34336	43635	1.020	1.296	1.271
Lipid transport and metabolism	Jabba	B7YZK6;B7YZK4;B7YZK5	0.030	11988	9599	15563	0.801	1.298	1.621
	Retinoid- and fatty acid-binding glycoprotein	Q9V496;L0MPS3;C7LAA3;Q1RKX2	0.047	49356	63617	70704	1.289	1.433	1.111
Protein modification, folding and turnover	Cathepsin K	Q9V3U6	0.019	22437	20186	27932	0.900	1.245	1.384
	CG2852 (peptidyl-prolyl cis-trans isomerase activity)	Q9W227;E4NKJ7	0.019	21665	20863	29600	0.963	1.366	1.419
	Calreticulin	Q9U916;Q53YH3;P29413	0.032	3067	n/a	4266	n/a	1.391	n/a
	Cysteine proteinase-1	Q95029;C6SV44	0.028	5135	5348	8231	1.042	1.603	1.539
	Heat shock protein 26	X2JGG6;P02517	0.035	30615	29112	47709	0.951	1.558	1.639
	Heat shock protein 27	X2JC82;P02518	0.047	20436	22212	31162	1.087	1.525	1.403
	Hsc/Hsp70-interacting protein related	Q86DS1;E2QD63;C4NYP8;Q8MSG4	0.011	6698	5549	8482	0.828	1.266	1.529
	Regulatory particle non-ATPase 6	Q7KLV9	0.043	4228	4644	6671	1.098	1.578	1.437
RNA binding, processing and modification	Cup	X2J9P9;Q9VMA3;Q4V6K0	0.028	8097	8656	10667	1.069	1.317	1.232
	Hoi-polloi	Q9U3Z7;D2NUK9	0.005	11849	14386	9425	1.214	0.795	0.655
	Modulo	P13469;Q8IGX8	0.003	26872	27825	18460	1.035	0.687	0.663
	Rm62	E1JJ68;C7LAE4;P19109;I0B1P2	0.018	7583	7579	4883	0.999	0.644	0.644
	Squid	Q08473	0.001	12919	15763	11482	1.220	0.889	0.728
Signal transduction	14-3-3ζ	P29310;Q6LEH5	0.030	14772	9020	8203	0.611	0.555	0.909
	Terribly reduced optic lobes	M9NGK3;X2JDK9;M9NDM1;Q8IRV8;M9NET2;M9NGL3;Q8IRV9;Q9W4Y4;M9NE61;X2JCE8;M9NFS1;E1JJC0;M9NDL5;M9NES6;Q9W4Y3;M9NFR6;X2JE09;A0A023GRW4;Q8IRV7;X2JAC7;M9NE56;Q8MPN3;Q9NEF9;Q9NEG0;Q8MSR5;Q9NEF8; Q9NEG1;Q9NFS9;D5AEP5	0.037	22421	25876	14510	1.154	0.647	0.561
	EIF3-S10	Q9VN25	0.014	11660	12311	9863	1.056	0.846	0.801
	Eukaryotic initiation factor 2γ	Q24208;Q53XD3;P45975;I0DHL3	0.003	12599	12664	10647	1.005	0.845	0.841
	Eukaryotic initiation factor 4E	P48598;M9PBZ9;Q683T0	0.025	4429	6245	6543	1.410	1.478	1.048
	Ribosomal protein L10Ab	Q9VTP4;Q8IGE1;Q8IGJ3	0.023	40363	44072	35150	1.092	0.871	0.798
	Ribosomal protein L11	P46222;Q4V5R3	0.029	30692	30175	21043	0.983	0.686	0.697
	Ribosomal protein L13	P41126;M9PFF0	0.006	56428	57610	48172	1.021	0.854	0.836
	Ribosomal protein L18a	P41093;Q4V4E7	0.048	37637	38784	31183	1.030	0.829	0.804
	Ribosomal protein L19	P36241	0.048	17020	14915	13888	0.876	0.816	0.931
	Ribosomal protein L27	Q9VBN5	0.002	31757	32575	25258	1.026	0.795	0.775
	Ribosomal protein L28	Q9VZS5;D1Z3A1;Q8MZI5	0.008	44350	45705	37938	1.031	0.855	0.830
	Ribosomal protein L6	Q9V9W2;Q9V9W3	0.009	37125	36926	28661	0.995	0.772	0.776
	Ribosomal protein L7	X2J5G6;P32100	0.011	68713	69719	58811	1.015	0.856	0.844
	Ribosomal protein L7a	X2JCS6;P46223	0.008	54795	54948	44325	1.003	0.809	0.807
	Ribosomal protein L9	X2JDU0;P50882	0.048	27003	27094	20575	1.003	0.762	0.759

Translation, ribosomal structure and biogenesis	Ribosomal protein LP0	P19889;M9PG76	0.018	65271	64798	56376	0.993	0.864	0.870
	Ribosomal protein S10b	Q9VWG3;M9NEQ9;D4G7H1;C0H6Z8; B7ZWP7;Q9VB14	0.003	34453	37529	29419	1.089	0.854	0.784
	Ribosomal protein S11	L0CRF8;L0CRF4;L0CR49;L0CR44;L0CQR2; L0CQQ8;L0CPM0;L0CPL5;L0CPK9;L0CPE3; L0CPD9;Q0E9B6;A1Z8U9;C4JD65	0.034	16446	19467	14080	1.184	0.856	0.723
	Ribosomal protein S13	X2J950;Q03334	0.015	36509	37621	31640	1.030	0.867	0.841
	Ribosomal protein S14b	X2JCX8;P14130	0.007	46567	49103	38358	1.054	0.824	0.781
	Ribosomal protein S16	Q9W237	0.034	61826	61179	52429	0.990	0.848	0.857
	Ribosomal protein S19a	P39018;E2QD65	0.007	43794	47070	37766	1.075	0.862	0.802
	Ribosomal protein S2	P31009;M9PB84	0.024	51976	59187	46645	1.139	0.897	0.788
	Ribosomal protein S27	Q9VBU9	0.011	25357	25066	16355	0.989	0.645	0.652
	Ribosomal protein S4	X2JGM9;P41042	0.000	44813	50282	40796	1.122	0.910	0.811
	Ribosomal protein S6	P29327;H1ZYF1;D2NUH9;A8E700;Q95TP9	0.014	37947	40555	28134	1.069	0.741	0.694
	Ribosomal protein S7	Q9VA91;Q8IMI7	0.014	55141	59156	47788	1.073	0.867	0.808
	Ribosomal protein S8	Q8MLY8	0.006	82797	83872	69685	1.013	0.842	0.831
	Seryl-tRNA synthetase	Q9VQL1;Q95TC0	0.001	4164	2571	n/a	0.617	n/a	n/a
Wolbachia protein	Wolbachia surface protein	O52076;Q09TN6;Q7BVI0;O52077;B0ZSE1;B7TX60;B7TX59;U6SUJ5;Q73G97;A0EZP4;Q5EFK7;Q5EFK9;Q5EFK8	0.042	n/a	11471	7245	n/a	n/a	0.632

Table S4

Functional Classification	Protein name	UniProt IDs	P-value	Average LFQ intensity values			Relative abundance		
				Dsim Cured	Dsim *w*Ri	Dsim *w*Mel	*w*Ri/Cured	*w*Mel/Cured	*w*Mel/*w*Ri
Carbohydrate transport and metabolism	Glycogen phosphorylase	B4Q7M5	0.028	3936	4318	7416	1.097	1.884	1.717
Chromatin structure and dynamics	Histone H4	P84043;B4NVU5;B4Q4E3	0.030	65922	60198	42489	0.913	0.645	0.706
	Nucleophosmin	B4R0X8	0.017	3242	4681	n/a	1.444	n/a	n/a
Coenzyme transport and metabolism	Lost	B4QVH6	0.000	28160	25667	19790	0.911	0.703	0.771
Cytoskeleton and cell motility	Tropomyosin 2	B4NTC0	0.019	3122	3377	5312	1.081	1.701	1.573
Energy production and conversion	ATP synthase, subunit B	B4QNN6	0.047	5777	4385	4741	0.759	0.821	1.081
	ATP synthase, δ subunit	B4NTR2	0.030	2936	4954	3289	1.687	1.120	0.664
	Vacuolar H[+]-ATPase 55kD subunit	B4QS89	0.017	4932	4993	5721	1.012	1.160	1.146
Function unknown	Female-specific independent of transformer	B4QZB3	0.003	3920	3079	13166	0.785	3.359	4.277
Lipid transport and metabolism	CG3902 (Acyl-CoA dehydrogenase/oxidase activity)	B4QPX7	0.023	7409	5981	5826	0.807	0.786	0.974
	Retinoid- and fatty acid-binding glycoprotein	B4R2I4, B4R2I5	0.005	8203	13544	13649	1.651	1.664	1.008
	Yolk protein 1	B4R7I5	0.000	269154	378786	535574	1.407	1.990	1.414
	Yolk protein 2	B4R7I4	0.007	342466	479537	579664	1.400	1.693	1.209
	Yolk protein 3	Q9N6E0;Q9NGD2	0.005	295946	292350	429476	0.988	1.451	1.469
Protein modification, folding and turnover	Heat shock protein 26	B4QN50	0.005	20510	24471	35945	1.193	1.753	1.469
	Heat shock protein 27	B4QN53	0.039	15891	10028	20642	0.631	1.299	2.058
	Proteasome α6 subunit	B4Q8Q8	0.032	4301	4131	5607	0.960	1.304	1.357
	Proteasome β7 subunit	B4QWA7	0.025	1788	n/a	2360	n/a	1.320	n/a
	Rripeptidyl-peptidase II	B4QDY3	0.003	3790	2657	4663	0.701	1.230	1.755
Protein modification, folding and turnover * Translation, ribosomal structure and biogenesis *	Ribosomal protein L40	B4R5A1;B4QCQ5	0.045	152215	154390	173408	1.014	1.139	1.123
RNA binding, processing and modification	Fibrillarin	B4QI53	0.009	6841	7446	3335	1.089	0.488	0.448
	Poly-a-binding protein	B4QBV7	0.001	17908	16708	12776	0.933	0.713	0.765
	Squid	B4R174	0.023	8718	4959	5905	0.569	0.677	1.191
Signal transduction	Receptor of activated protein kinase C 1	B4Q5U2;F6JMF8;F6JGS8;A9YGS6;B5LFR9; B5LFR8;B4R3L2;REV__B4QEY7	0.008	36393	34459	30338	0.947	0.834	0.880
Transcription	CG10576 (DNA binding activity)	B4QIS0	0.032	16667	16384	13178	0.983	0.791	0.804
Translation, ribosomal structure and biogenesis	Ribosomal protein L15	B4NTI4	0.005	23888	21396	17209	0.896	0.720	0.804
	Ribosomal protein L18	B4QKA8	0.030	17629	17476	12949	0.991	0.735	0.741
	Ribosomal protein L23A	B4QMI6	0.012	43012	42206	31778	0.981	0.739	0.753
	Ribosomal protein L28	B4QP55	0.013	33704	32566	26086	0.966	0.774	0.801
	Ribosomal protein L3	B4QU67	0.006	16310	15178	12720	0.931	0.780	0.838
	Ribosomal protein L32	P61128;G8E4T2	0.005	9627	9265	6060	0.962	0.629	0.654
	Ribosomal protein L34b	B4QWN3;B4QW27	0.017	14205	9295	8424	0.654	0.593	0.906
	Ribosomal protein L4	B4QY54	0.022	86811	85209	68446	0.982	0.788	0.803
	Ribosomal protein L7	B4Q8Q1	0.002	47341	45085	35545	0.952	0.751	0.788
	Ribosomal protein L7A	B4R5M2	0.019	38010	35461	29357	0.933	0.772	0.828
	Ribosomal protein L8	B4QNJ4	0.008	17851	17554	14679	0.983	0.822	0.836
	Ribosomal protein L9	B4QA88	0.025	20498	21827	16750	1.065	0.817	0.767
	Ribosomal protein LPO	B4QKR0	0.016	43554	43127	35953	0.990	0.825	0.834
	Ribosomal protein S14a	B4NU46	0.020	17020	17579	13280	1.033	0.780	0.755
	Ribosomal protein S15	B4QI06	0.038	13937	14884	10478	1.068	0.752	0.704
	Ribosomal protein s16	B4QHJ0	0.015	49676	47179	37758	0.950	0.760	0.800
	Ribosomal protein S18	Q5R288	0.022	39475	38472	31255	0.975	0.792	0.812
	Ribosomal protein S25	B4QU71	0.006	27697	26492	18027	0.957	0.651	0.680
	Ribosomal protein S27	B4QUR4	0.042	18338	19108	13732	1.042	0.749	0.719
	Ribosomal protein S3	B4R1G3	0.019	53474	51763	44074	0.968	0.824	0.851
	Ribosomal protein S6	B4R680	0.008	26527	26296	21186	0.991	0.799	0.806
	Ribosomal protein S8	B4R0U6	0.018	58446	55065	44484	0.942	0.761	0.808
	Ribosomal protein S9	B4QN82;B4NST5	0.012	55263	53490	44221	0.968	0.800	0.827
	Stubarista	Q0H6L1;B4R2Z5	0.033	37674	38399	30337	1.019	0.805	0.790

Table S5

Dmel Functional Classifications	Protein names	Current study, relative abundance			Brennan et al., 2008	Chevalier et al., 2012	Dangi et al., 2009	Hughes et al., 2011	Kremer et al., 2012	Pan et al., 2012	Rao et al., 2012	Sun and Kline, 2009	Yuan et al., 2015	Xi et al., 2008	Zhang et al., 2015	Zheng et al., 2011
		*w*Mel/Uninf	*w*MelCS/Uninf	*w*MelCS/*w*Mel												
Amino acid transport and metabolism	Eip55E	1.271	**1.587**	1.248	-	-	-	-	-	-	-	-	-	-	**Down**	**Up**
Carbohydrate transport and metabolism	Aldolase	0.919	**0.630**	0.685	-	-	-	**Down**	-	-	-	-	-	-	-	-
	Glycogen phosphorylase	0.839	1.447	**1.724**	-	-	-	**Down**	-	-	-	-	-	-	-	-
	Succinyl coenzyme A synthetase α subunit	n/a	n/a	**0.591**	-	-	-	-	-	-	-	-	-	-	-	**Up**
Chromatin structure and dynamics	Vig2	0.868	1.330	**1.533**	-	-	-	-	-	-	-	-	-	-	-	-
Cytoskeleton and cell motility	Ciboulot	0.858	1.380	**1.608**	-	-	-	-	-	-	-	-	-	-	-	-
Detoxification	Glutathione S-transferase D1	1.028	**1.626**	**1.581**	-	-	-	**Mixed**	**Down**	**Mixed**	-	-	-	-	**Down**	**Mixed**
	Peroxinectin-like	0.889	**1.704**	**1.917**	**Up**	**Mixed**	-	-	-	**Up**	-	-	-	-	-	-
	Transferrin 1	**2.000**	n/a	n/a	-	-	-	-	**Down**	-	-	-	-	-	-	**Up**
Energy production and conversion	ATP synthase, δ subunit	0.888	1.367	**1.540**	-	-	-	**Mixed**	-	-	-	-	-	-	-	-
	Isocitrate dehydrogenase	0.776	1.314	**1.693**	-	-	-	-	-	-	-	-	-	-	**Up**	-
Lipid transport and metabolism	Jabba	0.801	1.298	**1.621**	-	-	-	-	-	-	-	-	-	-	-	-
Protein modification, folding and turnover	Cysteine proteinase-1	1.042	**1.603**	**1.539**	-	-	-	**Down**	-	-	**Down**	-	**Down**	-	**Down**	-
	Heat shock protein 26	0.951	**1.558**	**1.639**	-	-	-	-	-	**Up**	-	-	-	-	-	-
	Heat shock protein 27	1.087	**1.525**	1.403	-	-	**Up**	-	-	-	-	-	-	**Down**	-	-
	Hsc/Hsp70-interacting protein related	0.828	1.266	**1.529**	-	-	-	-	-	-	-	-	-	-	-	-
	Regulatory particle non-ATPase 6	1.098	**1.578**	1.437	-	-	-	-	-	-	-	-	**Down**	-	-	-
RNA binding, processing and modification	Hoi-polloi	1.214	0.795	**0.655**	-	-	-	-	-	-	-	-	-	-	-	-
	Modulo	1.035	0.687	**0.663**	-	-	-	-	-	-	-	-	-	-	-	-
	Rm62	0.999	**0.644**	**0.644**	-	-	-	-	-	-	-	-	-	-	-	-
Signal transduction	14-3-3ζ	**0.611**	**0.555**	0.909	-	-	-	-	-	-	-	-	-	-	-	-
	Terribly reduced optic lobes	1.154	**0.647**	**0.561**	-	-	-	-	-	-	-	-	-	-	-	-
Translation, ribosomal structure and biogenesis	Ribosomal protein S27	0.989	**0.645**	**0.652**	-	-	-	-	-	-	-	-	-	-	-	-
	Seryl-tRNA synthetase	**0.618**	n/a	n/a	-	-	-	**Mixed**	-	-	-	-	-	-	-	-

Dsim Functional Classifications	Protein names	Relative abundance			Brennan et al., 2008	Chevalier et al., 2012	Dangi et al., 2009	Hughes et al., 2011	Kremer et al., 2012	Pan et al., 2012	Rao et al., 2012	Sun and Kline, 2009	Yuan et al., 2015	Xi et al., 2008	Zhang et al., 2015	Zheng et al., 2011
		*w*Ri/Cured	*w*Mel/Cured	*w*Mel/*w*Ri												
Carbohydrate transport and metabolism	Glycogen phosphorylase	1.097	**1.884**	**1.717**	-	-	-	**Down**	-	-	-	-	-	-	-	-
Chromatin structure and dynamics	Histone H4	0.913	**0.645**	0.706	-	-	-	-	-	**Up**	-	-	-	-	-	-
Cytoskeleton and cell motility	Tropomyosin 2	1.081	**1.701**	**1.573**	-	-	**Up**	**Down**	-	-	-	-	-	-	-	-
Energy production and conversion	ATP synthase, δ subunit	**1.687**	1.120	**0.664**	-	-	-	**Mixed**	-	-	-	-	-	-	-	-
Function unknown	Female-specific independent of transformer	0.785	**3.359**	**4.277**	-	-	-	-	-	-	-	-	-	-	-	-
Lipid transport and metabolism	CG3902 (Acyl-CoA dehydrogenase activity)	**1.651**	**1.664**	0.974	-	-	-	**Down**	-	**Up**	-	-	-	-	-	-
	Retinoid- and fatty acid-binding glycoprotein	**2.175**	**2.385**	1.008	-	-	-	-	-	-	**Up**	-	-	-	-	-
	Yolk protein 1	1.407	**1.990**	1.414	-	-	-	-	-	**Up**	-	**Down**	-	-	**Up**	-
	Yolk protein 2	1.400	**1.693**	1.209	-	-	-	-	-	**Up**	-	**Down**	-	-	**Up**	-
Protein modification, folding and turnover	Heat shock protein 26	1.193	**1.753**	1.469	-	-	-	-	-	**Up**	-	-	-	**Down**	-	-
	Heat shock protein 27	**0.631**	1.299	**2.058**	-	-	**Up**	-	-	-	-	-	-	-	-	-
	Tripeptidyl-peptidase II	0.701	1.230	**1.755**	-	-	-	-	-	-	-	-	-	-	-	-
RNA binding, processing and modification	Fibrillarin	1.089	**0.488**	**0.448**	-	-	-	-	-	**Up**	-	-	-	-	-	-
	Squid	**0.569**	*0.677*	1.191	-	-	-	-	-	-	-	-	-	-	-	-
Translation, ribosomal structure and biogenesis	Ribosomal protein L32	0.957	**0.651**	**0.654**	-	-	-	-	-	-	**Up**	-	-	-	-	-
	Ribosomal protein L34b	**0.654**	**0.593**	0.906	-	-	-	-	-	-	-	-	-	-	-	-
	Ribosomal protein S25	0.962	**0.629**	0.680	-	-	-	-	-	-	-	-	-	-	-	-

SUPPLEMENTAL REFERENCES

1. **Kremer N, Charif D, Henri H, Gavory F, Wincker P, Mavingui P, Vavre F.** 2012. Influence of Wolbachia on host gene expression in an obligatory symbiosis. BMC microbiology **12:**1.
2. **Zheng Y, Wang J-L, Liu C, Wang C-P, Walker T, Wang Y-F.** 2011. Differentially expressed profiles in the larval testes of Wolbachia infected and uninfected Drosophila. BMC genomics **12:**1.
3. **Chevalier F, Herbiniere-Gaboreau J, Charif D, Mitta G, Gavory F, Wincker P, Greve P, Braquart-Varnier C, Bouchon D.** 2012. Feminizing Wolbachia: a transcriptomics approach with insights on the immune response genes in Armadillidium vulgare. BMC Microbiol **12 Suppl 1:**S1.
4. **Hughes GL, Ren X, Ramirez JL, Sakamoto JM, Bailey JA, Jedlicka AE, Rasgon JL.** 2011. Wolbachia infections in Anopheles gambiae cells: transcriptomic characterization of a novel host-symbiont interaction. PLoS Pathog **7:**e1001296.
5. **Rao RU, Huang Y, Abubucker S, Heinz M, Crosby SD, Mitreva M, Weil GJ.** 2012. Effects of doxycycline on gene expression in Wolbachia and Brugia malayi adult female worms in vivo. J Biomed Sci **19:**21.
6. **Pan X, Zhou G, Wu J, Bian G, Lu P, Raikhel AS, Xi Z.** 2012. Wolbachia induces reactive oxygen species (ROS)-dependent activation of the Toll pathway to control dengue virus in the mosquito Aedes aegypti. Proc Natl Acad Sci U S A **109:**E23-31.
7. **Xi Z, Gavotte L, Xie Y, Dobson SL.** 2008. Genome-wide analysis of the interaction between the endosymbiotic bacterium Wolbachia and its Drosophila host. BMC Genomics **9:**1.
8. **Yuan LL, Chen X, Zong Q, Zhao T, Wang JL, Zheng Y, Zhang M, Wang Z, Brownlie JC, Yang F, Wang YF.** 2015. Quantitative Proteomic Analyses of Molecular

Mechanisms Associated with Cytoplasmic Incompatibility in Drosophila melanogaster Induced by Wolbachia. J Proteome Res **14:**3835-3847.

9. **Zhang YK, Ding XL, Rong X, Hong XY.** 2015. How do hosts react to endosymbionts? A new insight into the molecular mechanisms underlying the Wolbachia-host association. Insect Mol Biol **24:**1-12.

10. **Brennan LJ, Keddie BA, Braig HR, Harris HL.** 2008. The endosymbiont Wolbachia pipientis induces the expression of host antioxidant proteins in an Aedes albopictus cell line. PLoS One **3:**e2083.

11. **Dangi A, Vedi S, Nag JK, Paithankar S, Singh MP, Kar SK, Dube A, Misra-Bhattacharya S.** 2009. Tetracycline treatment targeting Wolbachia affects expression of an array of proteins in Brugia malayi parasite. Proteomics **9:**4192-4208.

12. **Sun S, Cline TW.** 2009. Effects of Wolbachia infection and ovarian tumor mutations on Sex-lethal germline functioning in Drosophila. Genetics **181:**1291-1301.
